# 2‐ and 2,7‐Substituted *para*‐*N*‐Methylpyridinium Pyrenes: Syntheses, Molecular and Electronic Structures, Photophysical, Electrochemical, and Spectroelectrochemical Properties and Binding to Double‐Stranded (ds) DNA

**DOI:** 10.1002/chem.202004748

**Published:** 2021-01-07

**Authors:** Goutam Kumar Kole, Julia Merz, Anissa Amar, Bruno Fontaine, Abdou Boucekkine, Jörn Nitsch, Sabine Lorenzen, Alexandra Friedrich, Ivo Krummenacher, Marta Košćak, Holger Braunschweig, Ivo Piantanida, Jean‐François Halet, Klaus Müller‐Buschbaum, Todd B. Marder

**Affiliations:** ^1^ Institut für Anorganische Chemie and Institute for Sustainable Chemistry & Catalysis with Boron Julius-Maximilians-Universität Würzburg Am Hubland 97074 Würzburg Germany; ^2^ Department of Chemistry College of Engineering and Technology SRM Institute of Science and Technology SRM Nagar Kattankulathur Tamil Nadu 603203 India; ^3^ Département de Chimie Faculté des Sciences Université Mouloud Mammeri 15000 Tizi-Ouzou Algeria; ^4^ Univ Rennes Ecole Nationale Supérieure de Chimie de Rennes CNRS Institut des Sciences Chimiques de Rennes UMR 6226 35000 Rennes France; ^5^ Division of Organic Chemistry and Biochemistry Ruđer Bošković Institute 10000 Zagreb Croatia; ^6^ Institut für Anorganische und Analytische Chemie Justus-Liebig-Universität Gießen Heinrich-Buff-Ring 17 35392 Gießen Germany

**Keywords:** chromophores, luminescent, pyrenes, pyridinium, viologens

## Abstract

Two *N*‐methylpyridinium compounds and analogous *N*‐protonated salts of 2‐ and 2,7‐substituted 4‐pyridyl‐pyrene compounds were synthesised and their crystal structures, photophysical properties both in solution and in the solid state, electrochemical and spectroelectrochemical properties were studied. Upon methylation or protonation, the emission maxima are significantly bathochromically shifted compared to the neutral compounds, although the absorption maxima remain almost unchanged. As a result, the cationic compounds show very large apparent Stokes shifts of up to 7200 cm^−1^. The *N*‐methylpyridinium compounds have a single reduction at ca. −1.5 V vs. Fc/Fc^+^ in MeCN. While the reduction process was reversible for the 2,7‐disubstituted compound, it was irreversible for the mono‐substituted one. Experimental findings are complemented by DFT and TD‐DFT calculations. Furthermore, the *N*‐methylpyridinium compounds show strong interactions with calf thymus (ct)‐DNA, presumably by intercalation, which paves the way for further applications of these multi‐functional compounds as potential DNA‐bioactive agents.

## Graphical abstract

**Multi‐functional pyrene‐viologens**: The synthesis, molecular and electronic structures, photophysical, electrochemical, and spectroelectrochemical properties of 2‐ and 2,7‐substituted *para*‐*N*‐methylpyridinium pyrene compounds are described along with detailed theoretical calculations. These compounds show strong interactions with calf thymus (ct)‐DNA, presumably by intercalation.

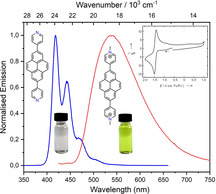

## Introduction

Pyrene, a polycyclic aromatic hydrocarbon (PAH), is one of the most widely investigated fluorophores, as it possesses some unique properties, such as intense blue emission, a high fluorescence quantum yield, a long‐lived singlet state, and propensity for excimer and exciplex formation.^[1]^ In addition to its rich photophysical properties, pyrene is chemically stable and can enhance intermolecular charge mobility.^[1]^ Many pyrene derivatives have, therefore, been developed for a wide range of applications in modern scientific fields which include organic optoelectronic devices, such as organic light emitting diodes (OLEDs), organic field‐effect transistors (OFETs), and organic photovoltaic cells (OPVs).^[2]^ The fluorescence of pyrene derivatives has been employed to investigate interactions in macromolecules and lipids,[^3^, ^4^] and also in probes for signalling of biomolecules, such as oligonucleotides and nucleic acids (e.g., DNA).^[5]^ In addition, their long fluorescence lifetimes and the possibility of excimer formation enable the exploration of advanced applications, such as the detection of DNA/RNA interactions both as a single label,^[6]^ as well as excimer‐forming pairs or as multi‐pyrene probes.^[7]^ The flat aromatic structure of pyrene derivatives facilitates its stacking with nucleobases and consequently intercalation into DNA/RNA.^[8]^ However, intercalative binding to DNA/RNA has limited selectivity and, therefore, some interesting applications of those pyrene derivatives rely on their interactions with DNA or RNA grooves^[9]^ or in combination with their turn‐on and ‐off excimers.^[10]^ Pyrene derivatives have further been employed for applications including the determination of cellular oxygen concentrations or reactive oxygen species (ROS) in biological systems,^[11]^ and the detection of various metal ions in solutions.^[12]^ In addition, as the fluorescence of pyrene‐based compounds can be influenced by external stimuli, this has been exploited for various applications such as sensing temperature,^[13]^ pressure,^[14]^ pH,^[15]^ and detection of small molecules.^[16]^ The ability of pyrene to exhibit strong π⋅⋅⋅π interactions makes it a suitable probe to examine arene‐perfluoroarene interactions and associated electrostatic quadrupole‐quadrupole interactions, even in dilute solutions, e.g., with hexafluorobenzene and octafluoronaphthalene.^[17]^ Furthermore, metal organic frameworks (MOFs)^[18]^ and covalent organic frameworks (COFs)^[19]^ have been reported with linkers containing a pyrene moiety.

Although pyrene is a symmetric molecule, its 10 peripheral reactive sites (C−H bonds) can be divided into three chemically inequivalent classes, depending on different electronic and steric features that correspond to different chemical reactivities.[^20^, ^21^] The 1‐, 3‐, 6‐, and 8‐positions of pyrene, having maximum contributions of the highest occupied molecular orbital (HOMO), are favourable for electrophilic aromatic substitution.^[22]^ Thus, the π‐orbitals of substituents at these positions participate very efficiently with pyrene's HOMO and LUMO. The 2‐ and 7‐positions of pyrene lie on a nodal plane in both the HOMO and LUMO, but are the least hindered sites. Therefore, sterically controlled reactions such as Ir‐catalysed direct borylation, are favourable at these sites.^[23]^ Moreover, the substituents at the 2‐ or 2,7‐positions do not interact with the HOMO/LUMO, but can interact strongly with the HOMO‐1 and LUMO+1 of pyrene that have non‐zero contributions at these positions.[^21^, ^24^, ^25^, ^26^] Consequently, the photophysical properties of pyrene derivatives with substituents at the 2‐ or 2,7‐ positions differ significantly from those with substituents at the 1‐, 3‐, 6‐, and 8‐positions. This is termed the “site‐effect”.[^20^, ^24^] It has also been demonstrated by us and others that judicious incorporation of a combination of strong π‐donors/acceptors at the 2‐ or 2,7‐positions of pyrene switches the order of its HOMO/HOMO‐1 and LUMO/LUMO+1, respectively, which in turn greatly influences the photophysical and electrochemical properties.[^21^, ^25^, ^26^] The localised double bond (C=C) character at the 4‐, 5‐, 9‐, and 10‐positions can be exploited to derivatise pyrene at its K‐region.[^27^, ^28^] Thus, various compounds with substituents at the 4‐, 5‐, 9‐, and 10‐positions with interesting photophysical and electrochemical properties have recently been reported.[^20^, ^29^, ^30^]

Methylviologens, or viologens in general, are di‐*N*,*N’*‐functionalised 4,4′‐bipyridyl salts, which have been extensively investigated in the past, and have attracted more attention recently.[^31^, ^32^] The possibility of having three stable redox states, efficient electron accepting capabilities (oxidizing agent), and tunability of the substituents at the nitrogen make them an important class of compounds. The most common viologen compound, *N*,*N’*‐dimethyl‐4,4′‐dipyridyl dichloride (MV^2+^), commonly known as „paraquat“, can undergo two reversible reductions, forming a radical‐cation (MV^C+^) and a neutral species (MV). Consequently, the colour changes from colourless (MV^2+^) to blue‐violet (MV^C+^), and finally to yellow‐brown (MV) during its redox processes.^[31]^ Such compounds have wide applications in electrochromic[^32^, ^33^] and photochromic materials.^[34]^ Moreover, viologen‐based materials have been explored over the years for their application in molecular shuttles and switches or machines.^[35]^ Recently, viologen‐based compounds have gained much importance for their use in organic „green batteries“.^[36]^ It should be noted that paraquat was one of the most widely used herbicides for decades; however, it has been banned in several countries due to its acute toxicity.[^37^, ^38^]

Depending on the substituents attached at the nitrogen atoms, and the counteranions, the colour of viologen compounds can change dramatically. Various counteranions have been utilised, halides being the most common and, recently, non‐coordinating anions such as triflate and hexafluorophosphate have been employed. The counteranions also play an important role in the stability and solubility of viologens.^[39]^ Significant efforts have been devoted to fine tune the electronic and photophysical properties of viologens by introducing further functionalisation such as additional bridging or incorporating a hetero‐atom in the molecule. A few examples are sulfide‐bridged viologens,^[40]^ phosphole‐bridged “phosphaviologen”,^[41]^ germanium‐bridged viologen,^[42]^ thiophene‐based,^[43]^ and π‐extended viologen compounds.^[44]^

**Scheme 1:**
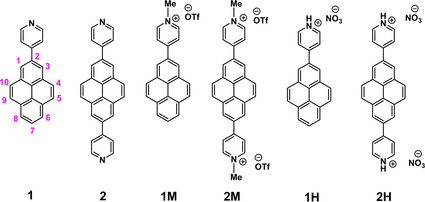
Compounds discussed in this study. Atom numbering of pyrene is also shown (left).

Recently, we have reported the syntheses and photophysical properties of several mono‐ and bis‐pyridyl‐pyrene compounds substituted at various positions of pyrene.^[20]^ It has been demonstrated that the protonation of pyridyl‐pyrene compounds shifts the emission bathochromically.^[45]^ The protonation of other fluorophores containing a conjugated pyridyl moiety has also been demonstrated to exhibit bathochromically shifted emissions.^[46]^ Thus, polyaromatic hydrocarbon (PAH)‐based viologen compounds or compounds containing pyridinium moieties have attracted attention recently due to their interesting photophysical properties which have various potential applications.^[47]^ Konishi and co‐workers reported a pyrene‐based viologen compound where the extended π‐conjugation was elongated by incorporating an ethenyl group between the pyrene and pyridinium moieties. This acceptor‐π‐acceptor type dye exhibited an emission in the ‘tissue transparent window’ (650–1100 nm) and a two‐photon absorption band around 1000 nm with a large cross‐section, and thus performed better than analogous PAH dyes (e.g., with naphthalene and anthracene) in three‐dimensional (3D) imaging of mitochondria in living cells.^[48]^ The photophysical properties of analogous 1,6‐bis(*N*‐methyl‐3’‐pyridinium)pyrene, 1,6‐bis(*N*‐methyl‐4’‐pyridinium)pyrene and 1,3,6,8‐tetrakis(*N*‐methyl‐4’‐pyridinium)pyrene have been reported along with their energy transfer processes on a clay surface in aqueous media.^[49]^ However, detailed investigation of the photophysical properties along with electrochemical and spectroelectrochemical studies, and the interactions with biorelevant targets (e.g., DNA) of pyridinium‐pyrene compounds have not been documented so far. Herein, we report the structures, photophysical and electrochemical properties of mono‐ and bis‐(*N*‐methylpyridinium)‐pyrene and analogous protonated compounds (Scheme 1), as well as preliminary studies on DNA binding of cationic compounds **1M** and **2M**.

## Results and Discussion

### Synthesis

The neutral compounds **1** and **2** were synthesised via the Suzuki–Miyaura coupling reaction of 2‐(Bpin)pyrene and 2,7‐bis(Bpin)pyrene, respectively, with 4‐bromopyridine, as we have reported previously;^[20]^ however, [Pd(dppf)Cl_2_] was used as the catalyst precursor instead of [Pd(PPh_3_)_4_]. The methylation reactions yielding compounds **1M** and **2M** were carried out with MeOTf in dry toluene under an argon atmosphere, and single crystals of **1M** and **2M** were obtained by slow evaporation of concentrated solutions of the compounds in MeCN. The salts **1H** and **2H** were obtained by reacting **1** and **2**, respectively, with HNO_3_ in MeCN, and their single crystals were obtained by slow evaporation from MeCN and DMF solutions, respectively. All compounds were characterised by various spectroscopic methods. In the ^1^H NMR spectrum, the signals for the protons of both the pyridyl and pyrene moieties were found to be down‐field shifted, as expected, upon protonation or methylation. For example, the signals for the pyridyl protons appear at 8.75 and 8.57 ppm for **2M** (Figure S7, Supporting Information), and at 9.04 and 8.57 ppm for **2H** (Figure S11, Supporting Information), and the signals for the pyrene protons appear at 8.85 and 8.40 ppm for **2M**, and at 9.0 and 8.43 for **2 H**, which are significantly down‐field shifted compared to the signals observed for **2** at 8.79 and 7.81 ppm (pyridyl protons), and 8.45 and 8.19 ppm (pyrene protons) (Figure S3, Supporting Information). Similar down‐field shifts were also observed for the signals of **1M** and **1H** compared to that of **1** (Figures S1, S5, S9, Supporting Information).

### Description of the crystal structures

**Figure 1:**
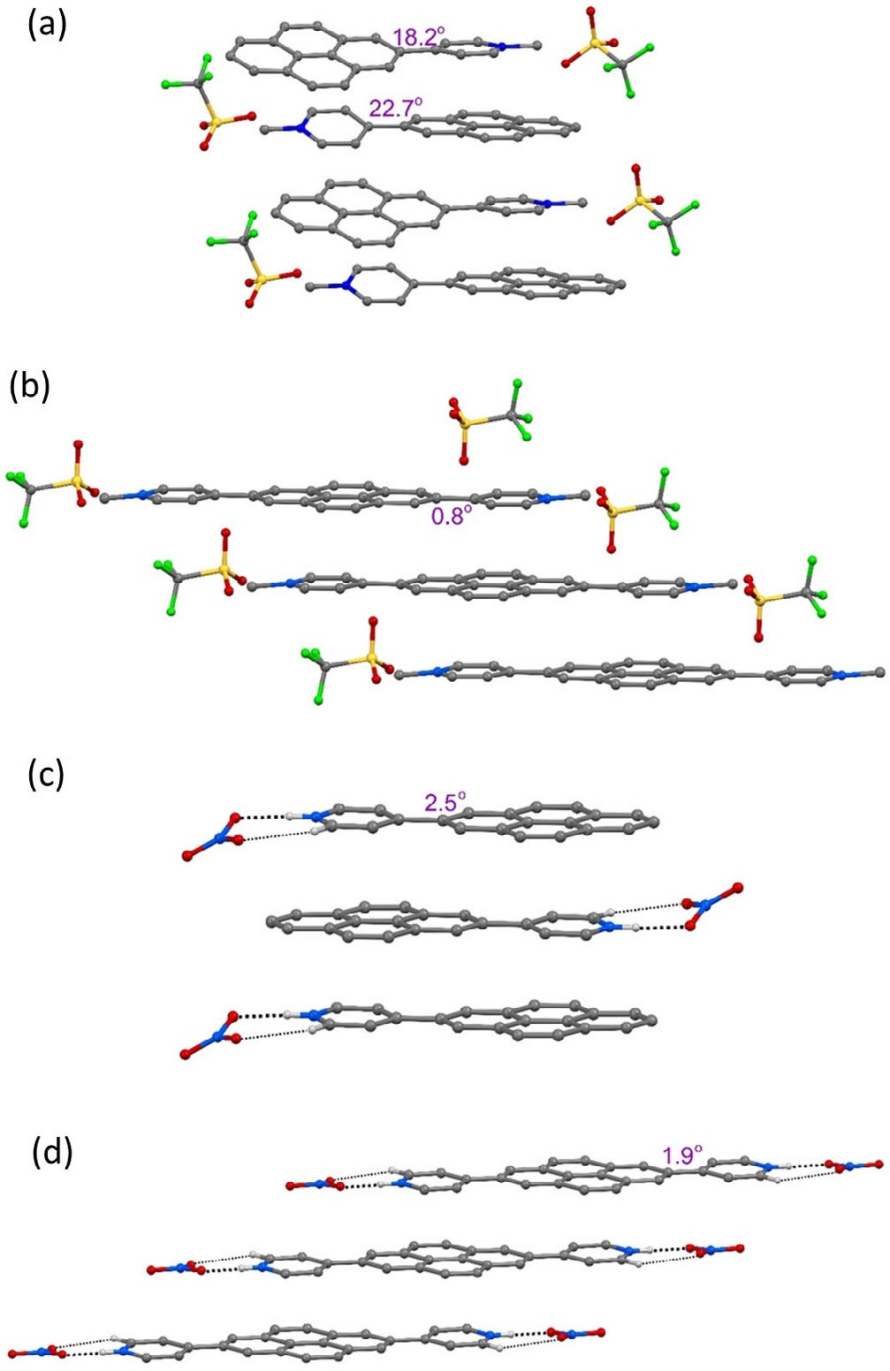
Molecular packing of (a) **1M**, (b) **2M**, (c) **1H**, and (d) **2H** in the solid state at 100 K. Only selected hydrogen atoms are shown for clarity. The numbers indicate the torsion angles between pyridyl and pyrene planes.

The crystal structures of compounds **1M**, **2M**, **1H** and **2H** were analysed by single‐crystal X‐ray diffraction. Among these, only the structure of **1M** is non‐centrosymmetric, and it was solved in the monoclinic space group *P*2_1_. The unit cell of **1M** contains two symmetry‐independent cations and two symmetry‐independent anions. The structures of the two cations differ mainly in the torsion angles between the planes of the pyridinium cations and the pyrene moieties, which are 18.2° and 22.7°, respectively (Figure 1 (a)). The cations stack in a parallel *head‐to‐tail* (anti) fashion and show π‐stacking (cation‐π) interactions between the pyridinium cation and pyrene moieties (interplanar separations (*d*) are in the range 3.34–3.41 Å). In addition, C−H⋅⋅⋅π interactions between these moieties with the methyl‐H atom are observed. The triflate anions exert various weak interactions including C−H⋅⋅⋅O and C−H⋅⋅⋅F interactions with the cations (Figure S13, Supporting Information).

The salt **1H** crystallizes in the monoclinic space group *P*2_1_/*c* and, in contrast to **1M**, only one symmetry‐independent cation (**1**‐H^+^) and nitrate ion are present in the unit cell. However, the molecular packing in **1H** is similar to that in **1M**, with the cations displaying parallel stacking in a *head‐to‐tail* fashion. The cation‐π interactions between the pyridinium cation and pyrene moieties (*d=*3.30–3.43 Å) are in a similar range to those in **1M**. However, in contrast to **1M**, the cation remains almost planar in **1H**, as the torsion angle between pyridinium and pyrene moieties is only 2.5°. N−H⋅⋅⋅O and weak C−H⋅⋅⋅O hydrogen bonding interactions are observed between the nitrate anion and the pyridinium cation (Figure 1 (c)).

The crystal structure of **2M** was refined in the monoclinic, centrosymmetric space group *C*2/*m*. The cation has 2/*m* (*C*_2*h*_) symmetry, while the triflate anion is disordered and has mirror‐symmetry (C_*s*_). The dications arrange in infinite, offset parallel stacks with strong π‐stacking (cation‐π) interactions between the pyridinium cation and pyrene moieties (*d=*3.35–3.37 Å) (Figure 1 (b)). Similar to the monocation **1H**, the dication of **2M** is almost planar with a torsion angle between the pyridinium and pyrene planes of 0.8°. The triflate anions interact with the dication via various weak C−F⋅⋅⋅π and C−H⋅⋅⋅O interactions (Figure S15, Supporting Information).

The salt **2H** crystallizes in the monoclinic space group *P*2_1_/*c* and the dication has inversion symmetry. The dications form offset parallel stacks with π‐stacking interactions between the pyridinium cation and pyrene moieties (*d=*3.37 and 3.39 Å) (Figure 1 (d)), similar to the arrangement in **2M**. As in **1H** and **2M**, the molecule is almost planar, as the torsion angle between the pyridinium and pyrene planes is 1.9°. The nitrate ions are involved in N‐H⋅⋅⋅O and weak C−H⋅⋅⋅O hydrogen bonding interactions with the dication (Figure S23, Supporting Information).

In summary, the mono‐ or dications are almost planar in all compounds in the solid state (torsion angles: 0.8–2.5°), except for **1M** (torsion angles: 18.2 and 22.7°). It is important to note that the observed torsion angles in the corresponding neutral compounds **1** (38.3°) and **2** (12.1° for α‐form and 38.6° for β‐form)^[20]^ are larger than those in the cationic compounds, which are strongly diminished by protonation or methylation (except for **1M**). The smaller torsion angles may also be a result of the large degree of π‐stacking interactions in the near‐planar cationic compounds. These show strong π‐stacking interactions between pyridinium and pyrene moieties as a result of protonation, which determine the parallel *head‐to‐tail* stacking in **1M** and **1H** and the offset parallel stacking in **2M** and **2H**. A similar π‐stacking interaction between pyridyl and pyrene moieties is not observed in the neutral compounds **1** and **2**, in which π‐stacking is only present between the pyrene moieties (**1**) or both the pyrene and the pyridyl moieties (**2**).^[20]^ In **1M**, **1H**, **2M** and **2H**, cation stacks weakly interact with the anions, which are located in between the stacks. A Hirshfeld surface analysis reveals the area and relative strengths of the intermolecular interactions and is presented in the Supporting Information (Figures S14 (**1M**), S18 (**2M**), S21 (**1H)**, and S24 (**2H)**). The powder diffraction patterns of all samples were measured in order to examine the phase purity of the bulk samples. The observed powder diffraction patterns of all compounds agree very well with the respective simulated ones (Figures S25 and S26 in the Supporting Information). Small systematic shifts of the reflection positions can only be observed in **1M** and **2M**, which may be due to the sample preparation.

### Photophysical properties

**Figure 2:**
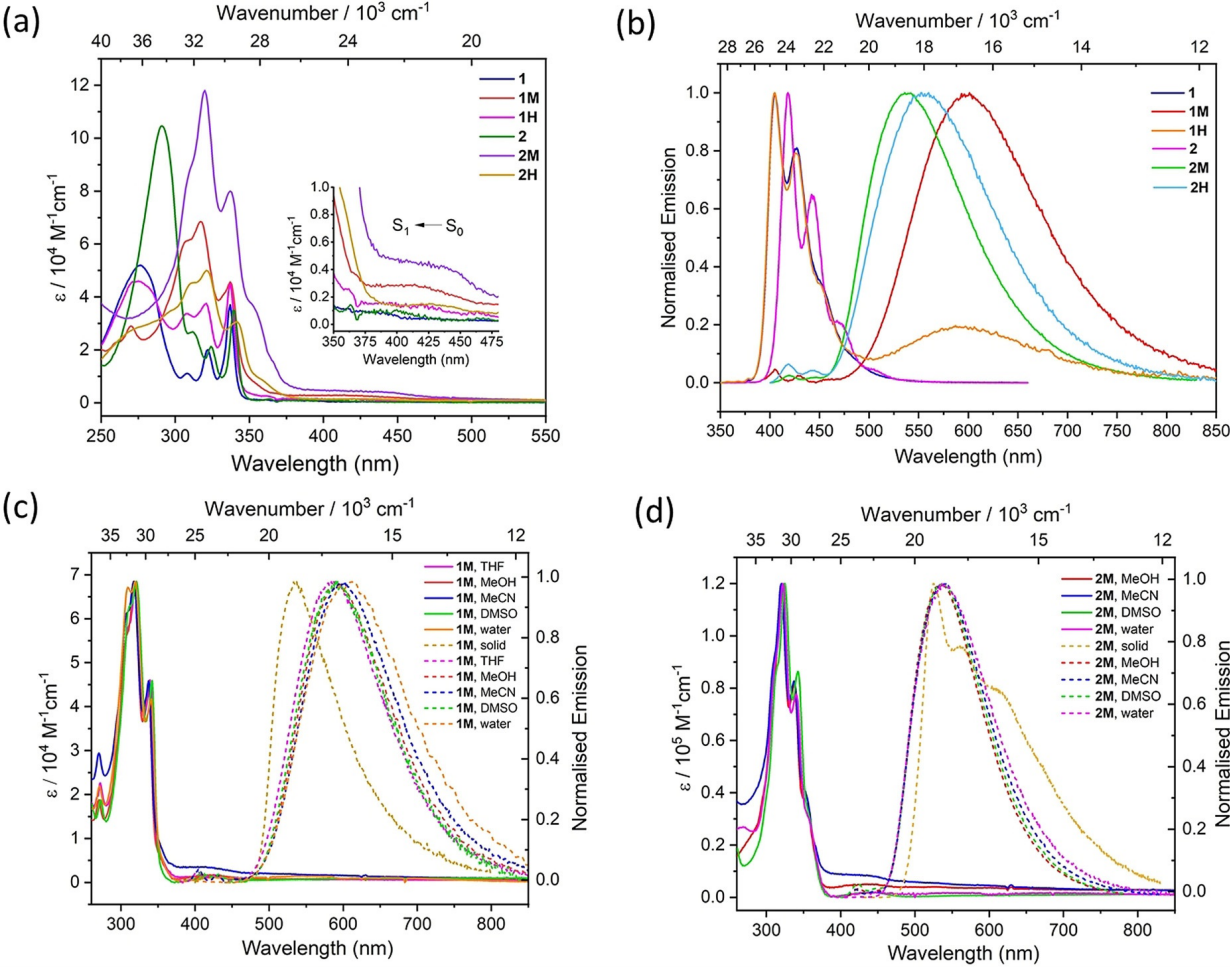
(a) Absorption, and (b) emission spectra of **1**, **2**, **1M**, **2M**, **1H**, and **2H** in MeCN (*λ*_ex_=337 nm); (c) absorption (solid lines), and emission (dashed lines) spectra of **1M** in THF, MeOH, MeCN, DMSO, water and in the solid state; and (d) absorption (solid lines) and emission (dashed lines) spectra of **2M** in MeOH, MeCN, DMSO, water and in the solid state. Very weak emission for **1M** at 405–430 nm and for **2M** at 420–440 nm might be due to the presence of traces of neutral compounds **1** and **2**, respectively. All measurements were carried out in deoxygenated solvents.

Detailed photophysical properties of all compounds are depicted in Figure 2. Absorption and emission were measured for **1M** in THF, MeOH, MeCN, DMSO, and water, for **2M** in MeOH, MeCN, DMSO, and water, as well as in the solid state for both compounds. For **1H** and **2H**, the absorption and emission spectra in MeCN are presented. The key photophysical data are summarised in Table 1. The neutral compounds **1** and **2** were measured in MeCN and in the solid state for comparison with the cations.

**Table 1:** Photophysical data for **1**, **2**, **1M**, **2M**, **1H**, and **2H** both in solution and in the solid state.

Compound	Solvent or Solid	*λ*_abs_ [nm]	*ϵ* [m ^−1^ cm^−1^]	*λ*_em_ [nm]	Apparent Stokes shift^[a]^ [cm^−1^]	*Φ*	*τ* [ns]	*τ*_avg_^[b]^ [ns]	*τ*_0_^[c]^ [ns]	*k*_r_ [10^6^ s^−1^]	*k*_nr_ [10^6^ s^−1^]
**1**	MeCN	362 337 322 308	1100 37 000 21 000	451 427 405	2900	0.22	36.1	–	164	6.1	22
	solid	–	–	452 430		0.05	3.4 (32.6) 8.9 (37.6) 23.5 (29.8)	6.9			
**1M**	THF	418 337 319		586	6800	0.08	16	–	200	5	58
	MeOH	418 337 318		593	7000	0.05	12.6	–	252	4	75
	MeCN	418 337 317	2900 45 000 68 500	599	7200	0.10	25	–	250	4	36
	DMSO	416 342 321		592	7100	0.11	22.6	–	205	5	39
	water	425 340 318 309		608	7080	0.01	3	–	300	3.3	330
	solid	–	–	535	–	0.08	18.6 (60.7) 59.6 (39.3)	25.5			
**1H**	MeCN	421 337 319	^[d]^	590 426 404	6800	–	–	–	–	–	–
	solid	–	–	545		0.05	8.4 (69.6) 23.2 (30.4)	10.3			
**2**	MeCN	363 339 324 312	1400 35 000	469 442 418	3600	0.41	56.6	–	138	7.2	10
	solid	–	–	482 453	–	0.02	1.3 (24.8) 5.6 (34.3) 13.8 (40.9)	3.5			
**2M**	MeOH	438 337 320		536	4100	0.09	15	–	167	6	61
	MeCN	437 337 320	4300 80 000 12 0000	538	4300	0.19	27	–	142	7	30
	DMSO	440 342 325		537	4100	0.22	22.2	–	101	10	35
	water	443 339 321		540	4050	0.12	15.5	–	129	7.7	57
	solid	–	–	612 560 525	–	0.03	3.3 (79.5) 13.4 (20.5)	3.9			
**2H**	MeCN	428 341 321	1530 30 500 50 000	555	5300	0.07	15.6	–	223	6.4	60
	solid	–	–	642	–	0.0	1.2 (69) 4.8 (19.9) 19.9 (11.1)	4	–	–	–

[a] The Stokes shift is defined as the energy difference between the 0‐0 transitions of the absorption and the emission. However, as the S_1_←S_0_ transitions in substituted pyrene derivatives are usually broad with an undefined vibrational pattern, the band maxima of the absorption and emission have been used to determine the Stokes shift; consequently, we use the term „apparent Stokes shift“. [b] For multi‐exponential decays, the experimental average lifetime is given by *τ*_avg_=∑*τ*_n_ *B*_n_/ ∑*B*_n_, where *B*_n_ are pre‐exponential factors of the respective lifetime *τ*_n_. [c] The pure radiative lifetime, *τ*_0_=*τ*/*Φ*; *k*_r_=1/*τ*_0_; *k*_nr_=(1−*Φ*)/*τ*. [d] Partially dissociates in solution, see text.

The absorption spectra of **1** and **2** in MeCN are very similar and demonstrate „pyrene‐like“ absorptions in that the S_1_←S_0_ absorption (370–420 nm) is very weak with extinction coefficients *ϵ* below 1400 m ^−1^ cm^−1^ (Figure 2 (a)). In general, the S_1_←S_0_ absorption of pyrene and 2‐ and 2,7‐substituted pyrene derivatives is attributed to a short‐axis‐polarised and transition‐dipole‐forbidden transition.^[24]^ The S_2_←S_0_ transition was observed at 337 nm with vibrational progressions of 1382 and 1411 cm^−1^ for **1**, and at 339 nm with vibrational progressions of 1365 and 1187 cm^−1^ for **2**, which are much more intense (*ϵ*=37 000 and 35 000 m ^−1^ cm^−1^) than the S_1_←S_0_ absorption, as also observed for unsubstituted pyrene (*λ*_abs_=334 nm for S_2_←S_0_ absorption, *ϵ*=55 000 m ^−1^ cm^−1^).^[50]^ The S_1_←S_0_ absorption of **2** is slightly bathochromically shifted compared to **1**, by 175 cm^−1^. The absorption spectra of the cationic compounds **1M** and **2M** in MeCN are also very similar to that of pyrene, but show a stronger absorbance compared to **1** and **2** (Figure 2 (a)). Thus, their S_1_←S_0_ absorption is still a weak band with *ϵ*=3000–4300 m ^−1^ cm^−1^; however, this band is bathochromically shifted and broad, covering ca. 100 nm (375–475 nm). The corresponding S_2_←S_0_ transitions of both **1M** and **2M** are less influenced as they are also observed at 337 nm in MeCN with vibrational progressions of 1872 and 1576 cm^−1^, respectively. The absorption of **1H** appears to be the combination of the absorbance of **1** and **1M**. All of the absorption bands in **1** and **1M** are present in **1H** with proportionate intensity. It seems that **1H** undergoes dissociation in MeCN solution which is even more evident in the emission spectrum (Figure 2 (b)). The absorption of **2H** resembles that of **2M** in that its S_1_←S_0_ absorption (*λ*_abs_=428 nm, *ϵ*=1530 m ^−1^ cm^−1^) is also transition‐dipole forbidden and broad. However, this band in **2M** is further bathochromically shifted and more allowed compared to that in **2H**. The S_2_←S_0_ transition of **2H** occurs at 341 nm (*ϵ*=30 500 m ^−1^ cm^−1^) with a vibrational progression of 1827 cm^−1^.

Furthermore, the absorption spectra of **1M** and **2M** were measured in various solvents to investigate possible solvent effects (see Table 1 and Figure 2 (c and d)). A very small shift (from 337 nm to 340 nm) was observed for **2M** in DMSO, which shows a bathochromic shift of only 430 cm^−1^ compared to its absorption in MeOH or MeCN. Thus, no significant solvatochromism was observed in the absorption spectra, which was further confirmed by theoretical calculations (vide infra).

Although these compounds exhibited similar absorption characteristics, their emission characteristics are quite different and display interesting features. The emission of the neutral compounds **1** and **2** are pyrene‐like, which agrees with their absorption properties. Thus, the emission of **1** at *λ*_em_=405 nm in MeCN shows a vibrational progression of 1250 cm^−1^. The emission of **2** at *λ*_em_=418 nm is slightly bathochromically shifted compared to that of **1** and also possesses a vibrational progression of 1300 cm^−1^. On the other hand, the emissions of the cationic compounds **1M** and **2M** are different than that of pyrene. Their emission consists of a single broad band, which is strongly bathochromically shifted compared to **1** and **2**, respectively. The emission of **1M** occurs at *λ*_em_=599 nm in MeCN with an apparent Stokes shift of 7200 cm^−1^, which is the most bathochromically shifted in this series. It can be noted that this apparent Stokes shift is very large for 2‐ or 2,7‐substituted pyrenes,[^20^, ^21^, ^24^] which indicates a large change of the dipole moment in the excited state. Considering the energy of maximum emission intensity of **1** (*λ*_em_=405 nm) and **1M** (*λ*_em_=599 nm), the emission of **1M** is bathochromically shifted by 8000 cm^−1^, which is a large effect that is introduced by one methyl group. The emission of **2M** occurs at *λ*_em_=538 nm with an apparent Stokes shift of 4300 cm^−1^. Considering the energy of maximum emission intensity of **2** (*λ*_em_=418 nm) and **2M**, (*λ*_em_=538 nm), the emission of **2M** is bathochromically shifted by 5300 cm^−1^. Interestingly, the emission of **1M** is even more bathochromically shifted than **2M**, which is an effect of a larger stabilization of the excited state in **1M** due to the larger dipole moment compared to **2M**, which has a symmetrical charge distribution. It can be noted that the emission of **2M** is hypsochromically shifted compared to analogous 1,6‐bis(*N*‐methyl‐3’‐pyridinium)pyrene dichloride (*λ*_em_=591 nm)^[49a]^ and bathochromically shifted compared to [1,3,6,8‐tetrakis(*N*‐methyl‐4‐pyridinium)pyrene][PF_6_]_4_ (*λ*_em_=485 nm).^[49b]^

To investigate the possible charge‐transfer (CT) nature in derivatives **1M** and **2M**, we conducted emission measurements in various solvents (see Table 1 and Figures 2 (c) and (d)). Indeed, the solvatochromic effect is very small. Very little change of *λ*_em_ with the polarity of the solvent was observed for **1M**. The difference of *λ*_em_ between THF (586 nm) and water (608 nm) is 22 nm, which is only 617 cm^−1^. However, it can be concluded that **1M** has greater CT nature than **2M**, which is also in line with the lower extinction coefficients of the first transition. Interestingly, the solid‐state emission of **1M** (*λ*_em(solid)_=535 nm) is considerably hypsochromically shifted compared to its solution emission (Figure 2(c)). On the other hand, the solid‐state emission of **2M** (*λ*_em(solid)_=612 nm, 560 nm, 525 nm) is comparable to its solution‐phase features (Figure 2(d)).

The emission of **1H** possesses two bands, one band at *λ*_em_=405 nm that shows a fine structure, which is identical to compound **1**, and also a broad band at 590 nm, which is similar to that of **1M**. Thus, it is evident that **1H** undergoes dissociation and maintains an equilibrium between the neutral and protonated forms. The emission of **2H** occurs at *λ*_em_=555 nm with an apparent Stokes shift of 5300 cm^−1^. Considering the energy of maximum emission intensity of **2** (*λ*_em_=418 nm) and **2M** (*λ*_em_=538 nm), the emission of **2H** is bathochromically shifted by 5900 cm^−1^ compared to that of **2**, and by 570 cm^−1^ compared to that of **2M**. Furthermore, we again observe that the mono‐substituted derivative **1H** has stronger CT character than its disubstituted analogue **2H**, as its emission is bathochromically shifted by 1070 cm^−1^.

The lifetime and quantum yields were measured in deoxygenated solutions. Monoexponential decays were obtained for the neutral and cationic compounds, and it is found that the neutral compounds possess a longer‐lived excited state compared to their cationic analogues (see Table 1). Furthermore, the non‐radiative decay rates of the latter ones are significantly increased (*k*_nr_=5.8–6.1×10^7^ s^−1^), which is a result of their larger apparent Stokes shifts, fully in line with the energy gap law.^[51]^ Similarly, the quantum yields of the neutral compounds are much higher compared to their cationic analogues. All of the compounds possess very low quantum yields in the solid state, which might be due to the absence of an effective suppression of the nonradiative decay, in the presence of strong π‐stacking interactions. This would also explain their shorter lifetimes in the solid state.

We found in our previous work that the solid state emissions of **1** and **2** strongly depend on crystallinity and particle size.^[20]^ In addition, two polymorphic phases of **2 (α** and **β**) have also been reported. While **2β** displays a large π‐π overlap area, these interactions are much weaker in **2α**. It is feasible to assume that the solid‐state emission is strongly influenced by the local environment and by the proportion of this local environment to the overall sample (Figures S29 and S30 in the Supporting Information). The overall solid‐state emission spectrum is therefore caused by an overlap of the emission spectra of the different polymorphs. However, these differences disappear when the samples are dissolved in solution.

### Electrochemistry

**Figure 3:**
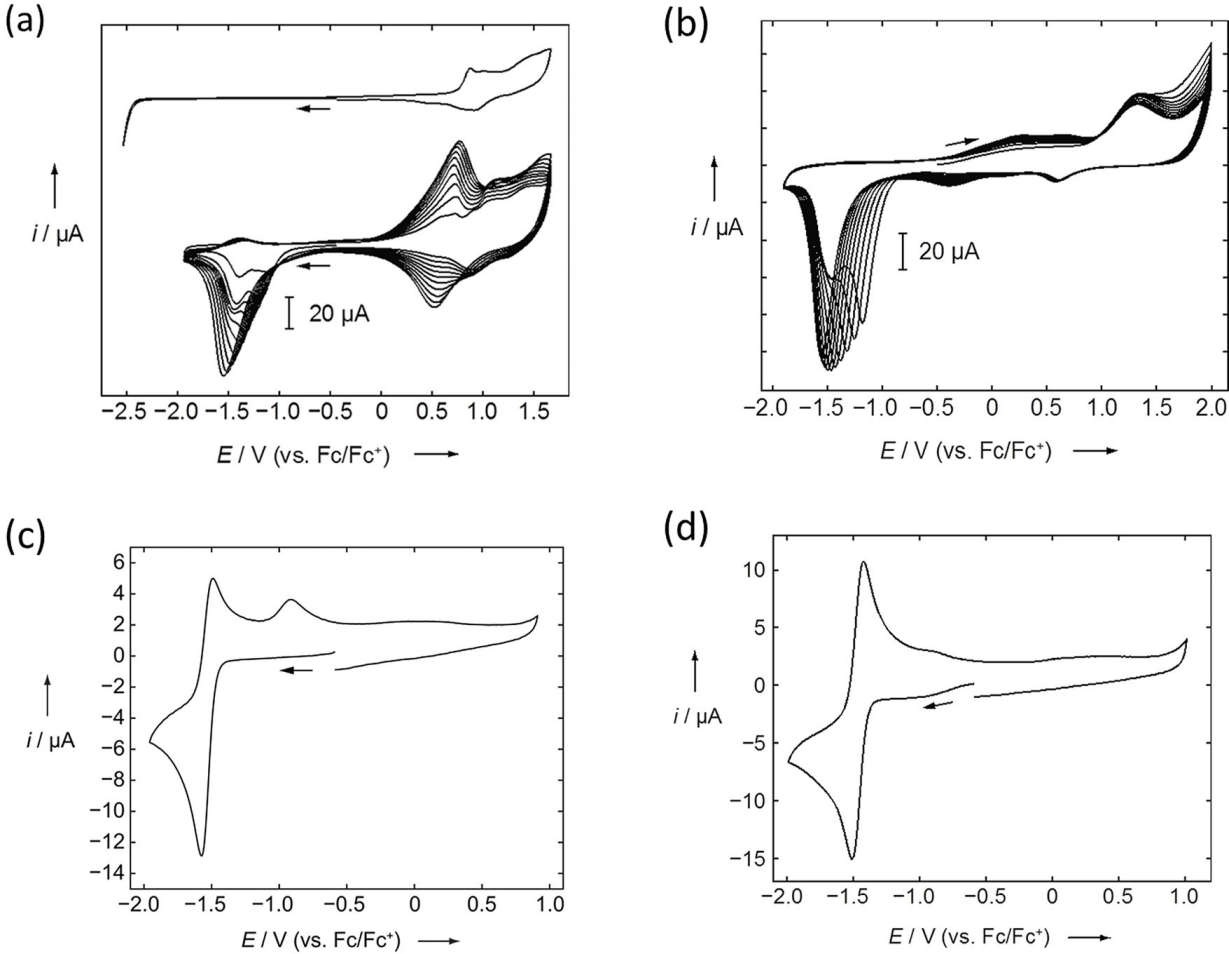
Cyclic voltammograms measured at 250 mV s^−1^ with 0.1 m [*n*Bu_4_N][PF_6_] relative to the Fc/Fc^+^ couple: (a) the first scan (*top*) and ten consecutive potential sweeps (*bottom*) of **1** in CH_2_Cl_2_; (b) ten consecutive potential sweeps of **2** in CH_2_Cl_2_; (c) **1M** in MeCN; (d) **2M** in MeCN.

Cyclic voltammetry studies were performed on the neutral and methylated compounds to investigate their electrochemical properties. The voltammograms were measured at a scan rate of 250 mV s^−1^ with 0.1 m [*n*Bu_4_N][PF_6_] relative to the Fc/Fc^+^ couple. The first scan and then ten consecutive potential sweeps were measured for **1** in CH_2_Cl_2_ as presented in Figure 3 (a). For the very first scan, the formal potentials are *E*_pa_=+0.87 and +1.02 V, in the cathodic direction; however, no reduction event was observed. The peak currents for the redox processes increased steadily with each successive scan. This was an indication that some chemical reaction (e.g., dimerisation or polymerisation) might take place and there was deposition on the electrode surface. It should be noted that electrochemical polymerisation of pyrene compounds has been reported previously.^[52]^ Electropolymerisation via anodic oxidation of unprotected bis(triarylamine)s leading to polymers with benzidine units is also known.^[53]^ In the case of **2**, ten consecutive potential sweeps are shown in Figure 3 (b). The formal potential *E*_pa_=+1.30 V was observed in the anodic direction; no reduction event was observed on the first scan. However, a reduction event can be observed only from the second scan. The reduction potential for the successive scans shifts to more negative values with a steady increase in the peak current (*E*_pc_=−1.60 V after 10 scans). This also indicates some chemical reaction or deposition on the electrode surface, as was observed for **1**.[^52^, ^53^] On the other hand, **1M** shows an irreversible reduction (Figure 3 (c)), which takes place at *E*_pc_=−1.53 V, and the corresponding oxidation process completes at −0.94 V. In the case of **2M**, a reversible reduction was observed with *E*_1/2_=−1.47 V, which can be attributed either to a two‐electron reduction or two very closely spaced consecutive one‐electron reductions leading to the formation of the neutral species. No further reduction processes for either **1M** or **2M** were observed within the accessible potential window for the solvent (even in MeCN). The observed formal potentials for these two compounds are comparable; however, only **2M** shows a reversible reduction. It can be noted that these observed reduction potentials for **1M** and **2M** occur at more negative values compared to the first one‐electron reduction of paraquat (MV^2+^), which occurs at −1.076 V vs. Fc^+^/Fc couple (the second one‐electron reduction of paraquat occurs at −1.51 V vs. Fc^+^/Fc couple in MeCN/[Et_4_N][PF_6_]).[^31c^, ^31d^, ^41a^, ^54^] Moreover, it can be noted that the extended methyl viologen 1,4‐bis(4'‐*N*‐methylpyridinium)benzene with [BF_4_]^−^ counteranions, in which the pyridinium groups are separated by a phenylene moiety, showed a single two‐electron reduction signal at −0.91 V vs. SCE (i.e., −1.29 V vs. Fc^+^/Fc couple)^[54]^ with 0.1 m MeCN/[Et_4_N][ClO_4_].^[31e]^ Another π‐extended viologen, i.e., 4,4’‐bis(4‐pyridinium‐*n*‐octyl)biphenyl with [PF_6_]^−^ counteranions, showed one reversible two‐electron reduction at −1.48 V vs. Fc/Fc^+^ in THF, in a range comparable to that of **2M**.^[44a]^ It can be further noted that the reduction of **2M** occurs at a much lower potential compared to that of our previously reported 2,7‐bis(BMes_2_)pyrene, an analogous acceptor‐pyrene‐acceptor system, which exhibits two reversible reduction events at −2.17 and −2.45 V vs. Fc/Fc^+^ in THF, indicating that the *N*‐methylpyridinium group is a stronger acceptor than ‐BMes_2_.^[25b]^

### Spectroelectrochemistry

**Figure 4:**
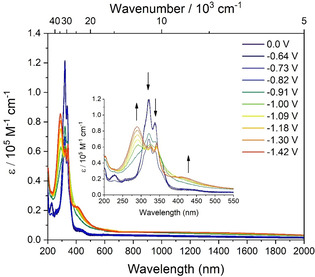
Spectroelectrochemical measurements of the stepwise reduction process of **2M** with 0.1 m [*n*Bu_4_N][PF_6_] in MeCN.

UV/Vis/NIR spectroelectrochemical measurements were carried out in CH_2_Cl_2_ for the neutral compounds **1** and **2**, and in MeCN for the methylated compounds **1M** and **2M**, with 0.1 m [*n*Bu_4_N][PF_6_] as the supporting electrolyte. Since reversible voltammograms were not observed for **1**, **2**_,_ and **1M** in the cyclic voltammetry, we have presented their spectra in the Supporting Information (Figures S31–S33). Upon stepwise reduction of **2M**, no absorption in the NIR region was observed; however, some changes in their UV/Vis absorption patterns were noticed (Figure 4). The absorption of reduced‐**2M** is very broad in the UV region with an extended tail in the visible region. The weaker broad peak at 400–450 nm increased, while the peak at 320 nm decreased upon reduction, and an intense peak at 295 nm appeared. Such an observation is attributed to the fact that **2M** undergoes either a two‐electron reduction or two very closely spaced consecutive one‐electron reductions, leading to the formation of the neutral species, without detecting the radical monocation.

### Theoretical calculations

Density functional theory (DFT) and time‐dependent density functional theory (TD‐DFT) calculations were carried out to rationalise the observed spectroscopic and electrochemical properties. The structures of these compounds were optimised at the PBE0/LANL2DZP level in THF including GD3BJ empirical dispersion (see Theoretical Computations in the Experimental Section). Selected experimentally determined and theoretically optimised structural parameters are listed in Tables S2 and S3 (Supporting Information). Overall, the optimised structural parameters for **1**, **2**, **1M**, **2M**, **1H**, and **2H** match well with the crystallographically determined parameters, except for the torsion angles for **1H** and **2H**, which deviate (ca. 25–30°) probably due to crystal packing effects in the solid state. Despite rather strong π‐stacking interactions observed between molecules in the cationic compounds (vide supra), periodic calculations on compound **2M**, for example, (see Theoretical Computations in the Experimental Section) seem to indicate that this stacking hardly alters the electronic properties of each molecule. Indeed, the density of states (DOS) of **2M** (Figure S34 in the Supporting Information) shows sharp peaks indicating weak electronic interactions between the dications in the solid.

HOMO–LUMO energy gaps are substantially larger for compounds **1** and **2** than for the cationic species (4.11 eV vs. ca. 3.15 eV, respectively), mainly due to some stabilization of the LUMO in the case of the latter ones. This suggests that the cationic compounds should absorb light at lower energies, as observed experimentally. This is supported by the maximum absorption and emission wavelengths which were also calculated at the TD‐DFT PBE0‐GD3BJ/LANL2DZP level in both MeCN and THF. No solvatochromic effect was observed in the calculations (see the comparison in Table S5 in the Supporting Information), which is in line with our experimental results. The main computed absorption wavelengths obtained in MeCN are given in Table 2 and compared with the experimentally observed values (see Table S5, in the Supporting Information, for values computed in THF). The corresponding simulated UV/Vis absorption spectra are shown in Figure S35 (Supporting Information). It can be noted that the calculated results agree better for the neutral compounds, with only a small deviation of a few nm, than for the methylated compounds, for which computed values are somewhat bathochromically shifted (ca. 850–4500 cm^−1^). Good agreement is also observed between computed and observed emission bands, although some blue shift is seen for the former ones with respect to the latter ones, especially for **1M** in MeCN (Table S6, Supporting Information). The predominant frontier molecular orbitals which are involved in the main electronic transitions are reported in Table 2. Our calculations show that the pyrene‐like nature of the transitions is generally maintained. In compounds **1** and **2**, pyrene's S_1_←S_0_ transition mostly involves a mixture of HOMO→LUMO+1 and HOMO‐1→LUMO electronic transitions and is transition dipole forbidden, and therefore has a very small oscillator strength, in agreement with the very small extinction coefficients observed experimentally (Table 2). In the cationic derivatives, the S_1_←S_0_ transitions remain forbidden as well with weak oscillator strengths, which is in agreement with the small extinction coefficients *ϵ* that we observed experimentally (1500–4300 m ^−1^ cm^−1^).

**Table 2:** Absorption wavelengths (*λ*_calc_, nm), oscillator strengths (*f*), and main electronic transitions calculated at the PBE0‐GD3BJ/LANL2DZP level in MeCN for compounds **1**, **2**, **1M**, **2M**, **1 H**, and **2 H**. Corresponding excited state numbers (S_*n*_) are given in brackets. Experimental wavelengths (*λ*_exp_, nm) and their extinction coefficients (*ϵ*, M^‐1^  cm^‐1^) measured in MeCN are given for comparison.

Compound	*λ*_exp_	*ϵ* [m ^−1^ cm^−1^]	*λ*_calc_	*f* (S_n_)	Main electronic transitions [weight %]^[a]^
**1**	362 337 276	1100 37 000 52 000	355 338 276	0.013 (S_1_) 0.331 (S_2_) 1.572 (S_5_)	H→L+1 (77 %); H‐1→L (21 %) H→L (90 %) H‐1→L+1 (91 %)
**1M**	418 337 269	2900 45 000 29 000	462 353 329 270	0.017 (S_1_) 0.031 (S_2_) 1.444 (S_3_) 0.142 (S_8_)	H→L (98 %) H‐1→L (58 %); H→L+1 (40 %) H→L+1 (53 %); H‐1→L (41 %) H→L+3 (36 %); H‐1→L+1 (26 %); H‐1→L+2 (20 %); H‐3→L (13 %)
**1H**	421 337 274	^[b]^	469 354 328 269	0.016 (S_1_) 0.034 (S_2_) 1.303 (S_3_) 0.146 (S_8_)	H→L (98 %) H‐1→L (61 %); H→L+1 (37 %) H→L+1 (55 %); H‐1→L (37 %) H→L+3 (34 %); H‐1→L+1 (26 %); H‐3→L (20 %); H‐1→L+2 (14 %)
**2**	363 339 291	1400 35 000 10 4000	371 342 296	0.017 (S_1_) 0.190 (S_2_) 2.553 (S_5_)	H→L+1 (79 %); H‐1→L (20 %) H→L (83 %); H‐1→L+1 (15 %) H‐1→L+1 (83 %); H→L (14 %)
**2M**	437 337 320	4300 80 000 12 0000	468 399 362 332	0.027 (S_1_) 0.000 (S_2_) 0.436 (S_3_) 2.126 (S_4_)	H→L (97 %) H→L+1 (99 %) H‐1→L (77 %); H→L+2 (22 %) H→L+2 (73 %); H‐1→L (21 %)
**2H**	428 341 321	1530 30 500 50 000	469 401 359 328	0.027 (S_1_) 0.000 (S_2_) 0.426 (S_3_) 1.887 (S_4_)	H→L (97 %) H→L+1 (99 %) H‐1→L (80 %); H→L+2 (19 %) H→L+2 (75 %); H‐1→L (19 %)

[a] H=HOMO and L=LUMO. [b] Partially dissociates in solution, see text.

**Figure 5:**
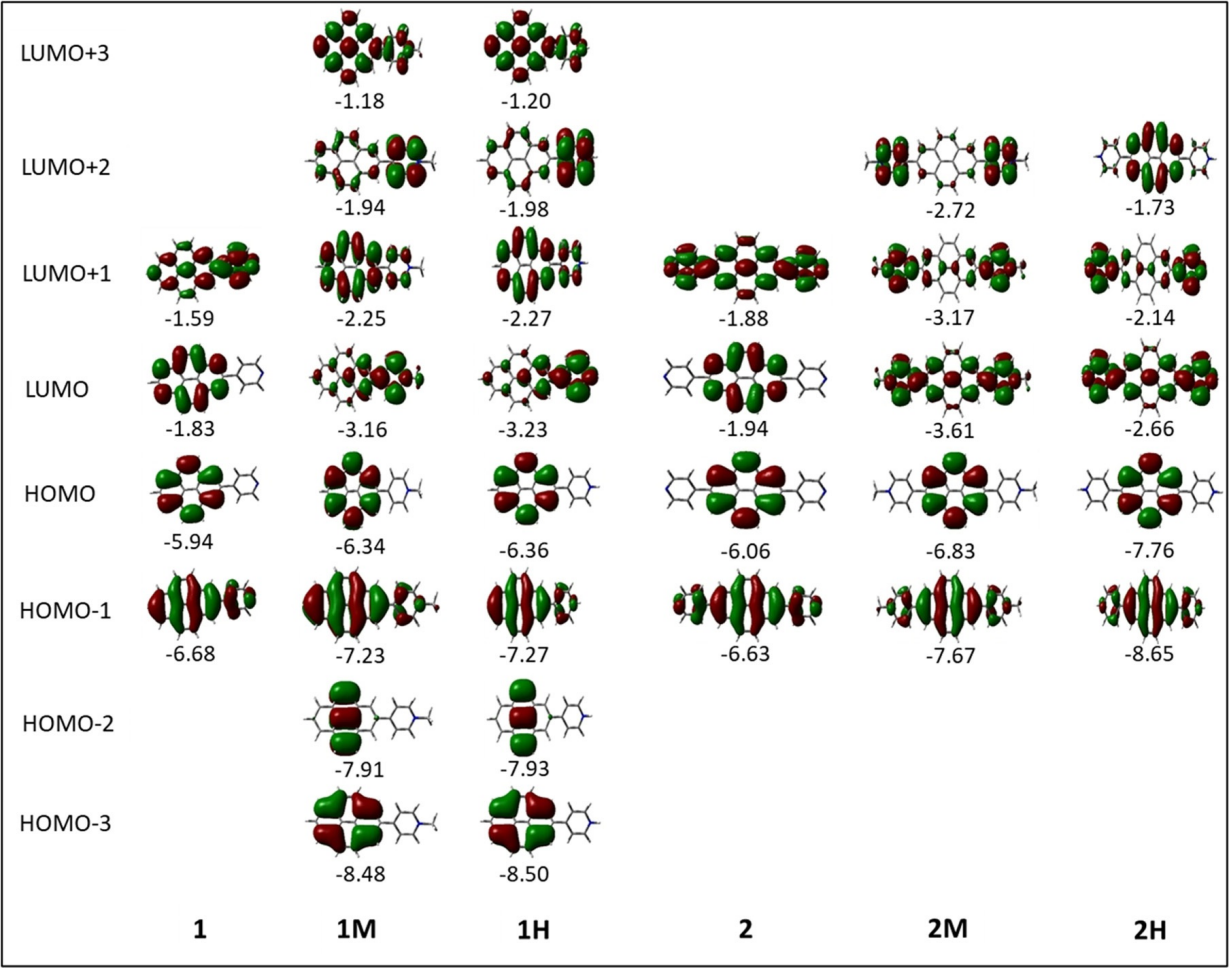
Representations of the frontier molecular orbitals and their energies (eV) calculated at the PBE0‐GD3BJ level in MeCN (isocontour value: ±0.03 au), involved in various electronic transitions.

As noted above, substituents at the 2‐ and 2,7‐positions do not contribute to pyrene's HOMO or LUMO.[^21^, ^24^, ^25a^, ^25b^, ^26a^] However, they can have a profound effect on pyrene's HOMO‐1 and LUMO+1. Indeed, orbitals of the pyridine moiety in the neutral compounds **1** and **2** mix with pyrene's HOMO‐1/LUMO+1, although not very efficiently. Consequently, the influence on the first transition is very small for **1** and **2** and, therefore, remains of HOMO‐1→LUMO and HOMO→LUMO+1 character. However, protonation (compounds **1H**/**2H)** or methylation (**1M**/**2M**) of the nitrogen, leads to a drastic stabilization of the LUMO+1, below the pyrene‐like LUMO (Figure 5). Hence, unlike pyrene, the LUMO of the cationic derivatives **1H**, **2H**, **1M**, and **2M** does not have a nodal plane through the long axis of the molecule, as observed previously with ‐BMes_2_ as the acceptor substituent.[^21^, ^24^, ^25b^] This explains the stronger bathochromic shift of the first transition and strongly altered emission in these cationic compounds compared to **1** and **2**. Our TD‐DFT calculations also show that the S_1_←S_0_ transitions in **1H**, **2H**, **1M**, and **2M** no longer involve a mixture of HOMO‐1→LUMO and HOMO→LUMO+1 electronic transitions as in pyrene or the neutral compounds **1** and **2**, but are nearly pure HOMO→LUMO electronic transitions. The CT character of the excitations is nicely illustrated by the difference density plots between the S_1_ and S_0_ states shown in Table 3. The transferred charges (*q*) for **1H** (*q* ^CT^=0.862 e) and **1M** (*q* ^CT^=0.878 e) are higher than for their disubstituted (symmetrical) analogues **2H** (*q* ^CT^=0.743 e) and **2M** (*q* ^CT^ **=**0.761 e), respectively. This is also fully in line with our observations which indicate that the S_1_←S_0_ transitions of **1H** and **1M** have smaller extinction coefficients, and their emissions are further bathochromically shifted compared to those of **2H** and **2M**, respectively, and that **1M** shows some degree of solvatochromism compared to non‐solvatochromic **2M** (Tables 1 and S5, Supporting Information).

**Table 3:** Density‐difference plots between relaxed excited state S_1_ and ground state S_0_ (with S_1_ geometry) (red=increase, blue=decrease of electron density) calculated at the PBE0‐GD3BJ/LANL2DZP level. Isocontour value: 0.03 au. *q* ^CT^ (e) and *d* ^CT^ (Å) correspond to the transferred charge and the distance between the positive (blue cross) and the negative (red cross) centroids of charge, respectively.

Compd	*q* ^CT^	*d* ^CT^		*ρ* ^S0^(r)−*ρ* ^S1^(r)
**1**	0.516	3.63	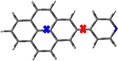	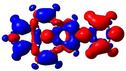
**1M**	0.878	4.551	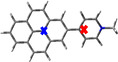	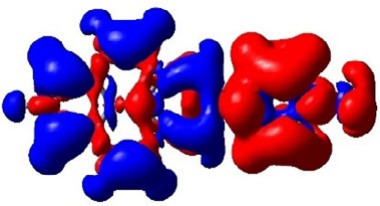
**1H**	0.862	4.482	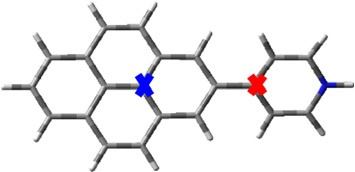	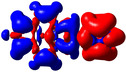
**2**	0.479	0.001	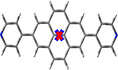	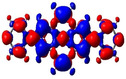
**2M**	0.761	0.004	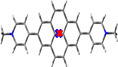	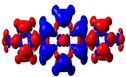
**2H**	0.743	0.002	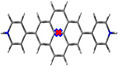	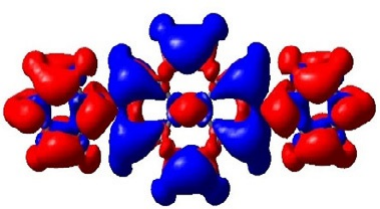

### Interactions with ds‐DNA

As many pyrene derivatives showed intriguing biorelevant interactions, acting as probes for various DNA/RNA sequences[^6^, ^7^, ^8^, ^9^, ^10^] and intracellular mitochondrial recognition,^[48]^ we performed preliminary studies on interactions of permanently charged *N*‐methylpyridinium derivatives **1M** and **2M** with the most commonly used ds‐DNA representative—*calf thymus* (ct)‐DNA. This DNA is characterised by a typical B‐helical secondary structure and almost equivalent number of GC‐(48 %) and AT‐(52 %) base‐pairs, thus representing a good target for both intercalative and minor groove binding mode. For the purpose of the study under biorelevant conditions, we prepared stock solutions of **1M** and **2M** in DMSO (1 mm), stored them at +4 ^o^C, and aliquots were used for dilution in buffer (sodium cacodylate buffer, *I*=50 mm, pH 7.0) prior to each measurement. The stability of the stock solutions was confirmed by checking the UV/Vis absorption or emission spectra of the freshly prepared solutions in buffer. The UV/Vis absorption properties in the buffer (Figure S37, Supporting Information) revealed very similar shape, as noted for other organic solvents (Figure 2). However, the change in ratio of absorbances at wavelengths 316 nm/306 nm (**1M**) and 336 nm/319 nm (**2M**) at higher concentrations (>5×10^−6^ m) suggests intermolecular aggregation (Figure S37, Supporting Information). Therefore, all further experiments were performed at concentrations <5×10^−6^ m.

As a preliminary screening of the interactions of our compounds with ds‐DNA, we performed thermal denaturation experiments. It is well known that, upon heating, the ds‐helices of polynucleotides dissociate into two single stranded polynucleotides at a well‐defined temperature (*T*_m_ value). Non‐covalent binding of small molecules to ds‐polynucleotides usually increases the thermal stability of the ds‐helices, thus resulting in an increased *T*_m_ value, and this increase (Δ*T*_m_) can (in corroboration with other methods) be related to the various binding modes.^[55]^ For example, if pyrene compounds intercalate into the ds‐DNA, Δ*T*_m_>2–5 °C can be expected, whereas the binding of pyrenes within the polynucleotide groove should have a negligible stabilising effect.^[56]^ We observed that the addition of **1M** monocation caused moderate stabilisation of the ct‐DNA against thermal denaturation (Δ*T_m_*=2 °C for r_[**1M**]/[ctDNA]_=0.2), whereas the dication **2M** yielded strong stabilisation (Δ*T_m_*>16 °C for r_[**2M**]/[ctDNA]_=0.2), and such stabilisation is indicative of an intercalative binding mode.

**Figure 6:**
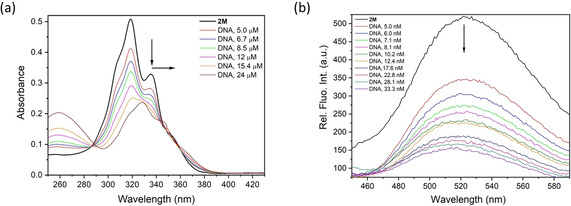
Fluorimetric titration of **2M** with ct‐DNA at pH 7.0, sodium cacodylate buffer, *I*=0.05 m: a) changes in UV/Vis absorption of **2M** (*c=*5×10^−6^ m); and b) changes in relative fluorescence intensity of **2M** (*c=*3×10^−8^ m).

The titration of **1M** or **2M** with ct‐DNA resulted in pronounced changes of their UV/Vis spectra (Figure 6(a) and Figures S40 and S41, Supporting Information), characterised by strong hypochromic and bathochromic effects typical of aromatic stacking interactions. Different responses were observed for **1M** and **2M** in the fluorimetric titrations with the ct‐DNA. For example, the emission of **1M** was primarily quenched up to ratio r_[**1M**]/[ctDNA]_=0.3 and, upon further additions of ct‐DNA, its emission intensity increased (Figure S42, Supporting Information). The quenching of emission occurred at an excess of **1M** over the ct‐DNA binding sites (r_[**1M**]/[ctDNA]_ >0.3) and, therefore, it was attributed to aggregation of **1M** along the ct‐DNA. The increased emission intensity at r_[**1M**]/[ctDNA]_ ≪0.3 indicates binding of single **1M** molecules to separate ct‐DNA binding sites. For the larger dicationic **2M**, the addition of ct‐DNA caused only quenching of emission (Figure 6 (b) and Figure S43, Supporting Information), which can be attributed mainly to the binding of single **2M** molecules to separate ct‐DNA binding sites. Analysis of the titration data by non‐linear fitting to Scatchard equation^[57]^ gave binding constants of log*Ks*=5.5 (for **1M**, data fitted only for r_[**1M**]/[ctDNA]_≪0.3) and log*Ks*=8.8 (for **2M**), respectively, for Scatchard ratio n_[bound **dye**]/[ctDNA]_=0.3. The difference in binding constants matched well with the observed difference in thermal denaturation (Δ*T_m_*) values. Thus, it can be inferred that the dicationic compound **2M** interacts more strongly with the ct‐DNA compared to the monocationic compound **1M**.

**Figure 7:**
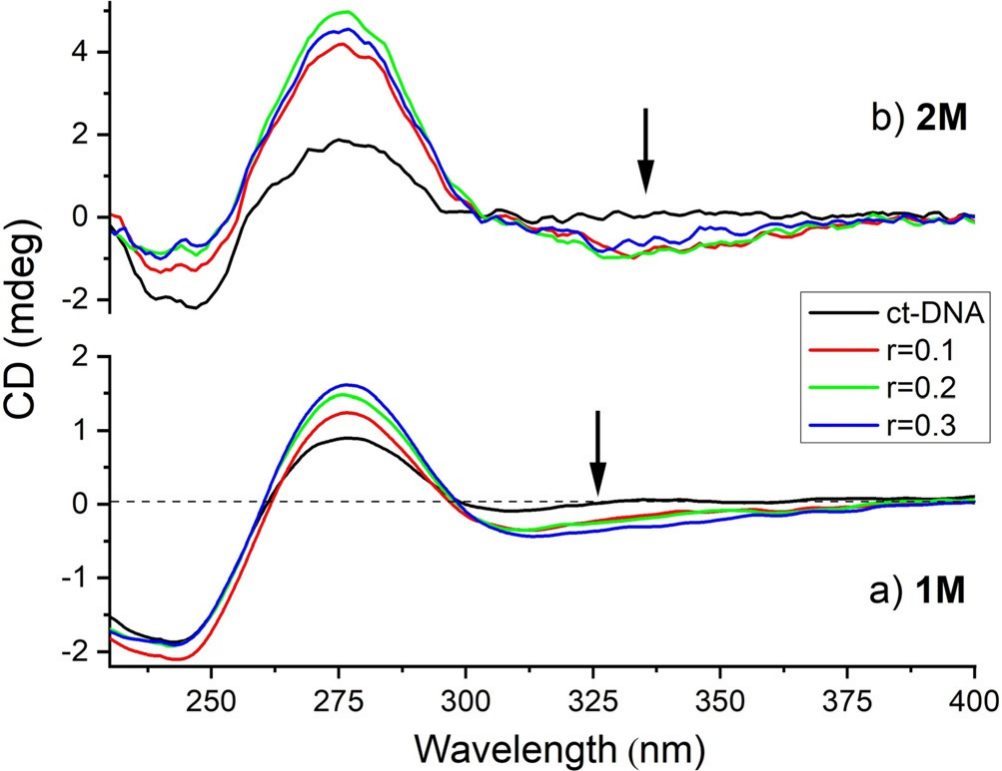
CD titration of ct‐DNA (*c*=2×10^−5^ m) with: a) **1M**; and b) **2M**; at molar ratios r=[compound]/[polynucleotide] and at pH 7.0, buffer sodium cacodylate, *I*=0.05 M.

In order to obtain additional insight into the structural properties of small molecule/polynucleotide complexes, we have chosen the circular dichroism (CD) spectroscopic method, which is highly sensitive to conformational changes in the secondary structure of polynucleotides. In addition, achiral small molecules, such as **1M** or **2M**, can acquire an induced CD spectrum (ICD) upon binding to polynucleotides, which can give useful information about the modes of interaction.^[58]^ Indeed, CD spectra (Figure 7) revealed weak negative ICD bands at *λ*=300–350 nm range for both, **1M** and **2M**. As pyrene absorbs in this range, such ICD bands are characteristic of the intercalative binding mode, which agrees well with the bathochromic and hypochromic changes in UV/Vis titrations, high binding constants and thermal denaturation results.

## Conclusions

We have presented structural, photophysical, and electrochemical studies on 2‐ and 2,7‐substituted pyridyl‐pyrenes and their *N*‐methylated compounds and analogous protonated salts, which are acceptor‐π and acceptor‐π‐acceptor systems, respectively. Their structure determination by X‐ray crystallography revealed that the torsion angle between the planes of pyrene and pyridyl moieties tends to become very small upon protonation or methylation, leading to almost planar structures, which agree well with the calculated optimised structures obtained at the PBE0‐GD3BJ/LANL2DZP level of theory. Although the absorption spectra show rather small changes upon protonation or methylation, their emission spectra were found to display large bathochromic shifts. The magnitude of such shifts is larger for the dipolar molecule **1M** than for the symmetric molecule **2M**. Thus, upon methylation of **1** (*λ*_em_=405 nm) and **2** (*λ*_em_=418 nm) to **1M** (*λ*_em_=599 nm) and **2M** (*λ*_em_=538 nm), respectively, their corresponding emission maxima shifted bathochromically by 8000 cm^−1^ and 5300 cm^−1^, respectively, in MeCN. Moreover, dipolar **1M** shows an apparent Stokes shift of 7200 cm^−1^, which is very large for 2‐ and 2,7‐substituted pyrenes, indicating a rather large change of the dipole moment in the excited state. Symmetric, dicationic **2M** shows an apparent Stokes shift of 4300 cm^−1^, which is smaller than that of **1M**, due to its lack of a dipole moment. Compound **1M** showed an irreversible reduction at −1.53 V, while **2M** showed a reversible reduction at −1.47 V vs. Fc^+^/Fc couple in MeCN. These observed formal potentials are in accordance with other similar π‐conjugated extended viologens reported in the literature. However, the reduction of **2M** occurs at a lower potential compared to 2,7‐bis(BMes_2_)pyrene, indicating that the *N*‐methylpyridinium group is a stronger acceptor than ‐BMes_2_. The reduction of **2M** can be attributed either to a two‐electron reduction or to two closely spaced consecutive one‐electron reductions, leading to the formation of the neutral species, without producing the intermediate radical‐cation, which was further established by investigating the step‐wise reduction processes by spectroelectrochemical measurements. Our experimental observations are further confirmed by theoretical calculations performed at both the PBE0‐GD3BJ and CAM‐B3LYP levels of theory. The HOMO of both the neutral and methylated/protonated compounds remain rather unaffected. However, upon methylation or protonation, the LUMOs of the neutral compounds are destabilised, while the LUMO+1 orbitals are greatly stabilised and become the LUMOs. Thus, the new LUMOs of the methylated/protonated compounds do not have a nodal plane passing through the long axis of the molecule. Furthermore, preliminary studies reveal that **1M** and **2M** interact strongly with ct‐DNA by intercalation into the double helix, which makes them very interesting compounds for further studies of their biological activity. Detailed investigation of these interactions and their significance in biological systems are ongoing. Our research findings are expected to have significant impact on further investigations on the photophysics and electrochemistry of other PAH‐derived π‐conjugated viologen compounds and their potential applications in biological systems.

## Experimental Section

### General

The catalyst precursor [Ir(OMe)(COD)]_2_ was prepared according to a literature procedure^[59]^ and B_2_pin_2_ was a gift from AllyChem Co. Ltd. The starting materials 2‐(Bpin)pyrene and 2,7‐bis(Bpin)pyrene were synthesised by following methods developed by Marder and co‐workers.^[23c]^ The syntheses of **1**, **2**, **1M**, and **2M** were carried out using standard Schlenk or glovebox (Innovative Technology Inc.) techniques under an argon atmosphere. All of the compounds are, in general, stable, and can be handled in air. All solvents used for reactions were HPLC grade, dried using an Innovative Technology Inc. Solvent Purification System, and were further deoxygenated and stored under argon. All other chemicals were purchased from common commercial sources and were used without further purification.

^1^H and ^13^C{^1^H} solution NMR spectroscopic data were obtained at ambient temperature using a Bruker Avance 300 NMR spectrometer, operating at 300 MHz for ^1^H and 75 MHz for ^13^C{^1^H}. Chemical shifts (δ) were referenced to solvent residual peaks. High resolution mass spectrometry (HRMS) was performed with a Thermo Fisher Scientific Exactive Plus Orbitrap MS System with either an Atmospheric Sample Analysis Probe (ASAP) or Atmospheric Pressure Chemical Ionization (APCI) or an electrospray ionization (ESI) probe. Elemental analyses were performed on an Elementar vario MICRO cube elemental analyser.

### Syntheses

Syntheses of compounds **1** and **2** were described previously.^[20]^

**Synthesis of 1M**: **1** (140 mg, 0.5 mmol) was stirred with methyl triflate (80 μl, 120 mg, 0.73 mmol) in dry toluene (10 mL) for 24 h under an argon atmosphere. The yellow precipitate obtained was isolated by filtration, washed with dry toluene and dried. Yield: 200 mg (90 %). Needle like single crystals were obtained by slowly evaporating an MeCN solution. ^**1**^ **H NMR** (300 MHz, CD_3_CN): *δ*=8.60 (d, *J=*7 Hz, 2 H), 8.59 (s, 2 H), 8.38 (d, *J=*7 Hz, 2 H), 8.30 (d, *J=*8 Hz, 2 H), 8.20 (AB multiplet, 4 H), 8.13 (t, *J=*8 Hz, 1 H), 4.27 ppm (s, 3 H). ^**13**^ **C{^1^H} NMR** (75 MHz, CD_3_CN): *δ*=157.1, 146.1, 132.7, 132.5, 131.6, 129.8, 128.5, 128.4, 127, 126.7, 126.1, 124.8, 124.6, 124.3, 120.1, 48.4 ppm. **HRMS** (ESI^+^): *M/Z* found: 294.1266; calculated *M/Z* for [M‐(OTf)]^+^ (C_22_H_16_N^+^)=294.1277. **Elem. Anal**. Calcd. (%) for C_23_H_16_NO_3_F_3_S: C 62.30, H 3.64, N 3.16, S 7.23; found: C 62.40, H 3.55, N 3.13, S 7.28.

**Synthesis of 2M**: **2** (180 mg, 0.5 mmol) was stirred with methyl triflate (180 μl, 270 mg, 1.64 mmol) in dry toluene (15 mL) over 24 h in an argon atmosphere. The yellow precipitate obtained was isolated by filtration, washed with dry toluene and dried under vacuum. Yield: 290 mg (85 %). Needle like single crystals were obtained by slowly evaporating a solution in MeCN. ^**1**^ **H NMR** (300 MHz, CD_3_CN): *δ*=8.85 (s, 4 H), 8.75 (d, *J=*7 Hz, 4 H), 8.57 (d, *J=*7 Hz, 4 H), 8.40 (s, 4 H), 4.36 ppm (s, 6 H). ^**13**^ **C{^1^H} NMR** (75 MHz, CD_3_CN): *δ*=133.5, 133.4, 129.9, 126.7, 126.2, 125.9, 123.5, 120.9, 48.7 ppm. **HRMS** (ESI^+^): *M/Z* found: 193.0883; calculated *M/Z* for [M‐2(OTf)]^2+^ (C_28_H_22_N_2_^2+^)=193.0886. **Elem. Anal**. Calcd. (%) for C_30_H_22_N_2_O_6_F_6_S_2_: C 52.63, H 3.24, N 4.09, S 9.37: found: C 52.62, H 3.28, N 4.16, S 9.12.

**Synthesis of 1H**: **1** (140 mg, 0.5 mmol) was placed in MeCN (20 mL) and a few drops of nitric acid were added. The mixture was heated to 60 °C until all compounds dissolved. The clear solution was allowed to evaporate slowly. Needle shaped single crystals were obtained after a few days. Yield: 150 mg (88 %). ^**1**^ **H NMR** (300 MHz, *d*_4_‐MeOD): *δ*=8.94 (m, 2 H), 8.81 (s, 2 H), 8.69 (m, 2 H), 8.32 (d, *J=*7 Hz, 2 H), 8.27 (AB multiplet, 4 H), 8.13 ppm (t, *J=*8 Hz, 1 H). **HRMS** (APCI^+^): *M/Z* found: 280.1116; calculated *M/Z* for [M‐(NO_3_)]^+^ (C_21_H_14_N^+^)=280.1121. **Elem. Anal**. Calcd. (%) for C_21_H_14_N_2_O_3_: C 73.68, H 4.12, N 8.18; found: C 73.53, H 4.13, N 8.12.

**Synthesis of 2H**: **2** (180 mg, 0.5 mmol) was placed in MeCN (20 mL) and a few drops of nitric acid were added. The mixture was stirred with heating at 60 °C for 24 h and then cooled to room temperature. The orange precipitate obtained was isolated by filtration. Yield: 210 mg (87 %). Needle shaped single crystals were obtained by recrystallization from DMF. ^**1**^ **H NMR** (300 MHz, [D_6_]DMSO): *δ*=9.04 (d, *J=*7 Hz, 4 H), 8.99 (s, 4 H), 8.58 (d, *J=*7 Hz, 4 H), 8.43 ppm (s, 4 H). **HRMS** (ESI^+^): *M/Z* found: 179.0722; calculated *M/Z* for [M‐2(NO_3_)]^2+^ (C_26_H_18_N_2_^2+^)=179.0730. **Elem. Anal**. Calcd. (%) for C_26_H_18_N_4_O_6_: C 64.73, H 3.76, N 11.61; found: C 63.94, H 3.69, N 10.92.

### Single crystal X‐ray diffraction

Single crystals suitable for X‐ray diffraction analysis were selected, coated in perfluoropolyether oil, and mounted on MiTeGen sample holders. Diffraction data were collected on a Bruker X8‐Apex II 4‐circle diffractometer with a CCD area detector using Mo‐*K*α radiation monochromated by graphite or multi‐layer focusing mirrors. Diffraction data were collected at 100 K. The mounted crystals were cooled by a stream of cold nitrogen using Oxford Cryostreams or Bruker Kryoflex II low‐temperature devices. The frames were processed and corrected for Lorentz‐polarisation and absorption effects by employing the Bruker software packages. The structures were solved using the intrinsic phasing method (SHELXT).^[60]^ All non‐hydrogen atoms were refined in anisotropic approximation, with hydrogen atoms ‘riding’ in idealised positions, by full‐matrix least squares against *F* ^2^ on all data, using SHELXL^[60]^ software. Hirshfeld surfaces were calculated and analysed using the Crystal Explorer program (version 17.5).^[61]^ Various structural information was extracted and graphics were produced using Mercury software.^[62]^ Crystal data and experimental details are listed in Table S1 in the Supporting Information. Deposition numbers 2016793 (**1H)**, 2016794 (**1M**), 2016795 (**2H)**, and 2016796 (**2M**) contain the supplementary crystallographic data for this paper. These data are provided free of charge by the joint Cambridge Crystallographic Data Centre and Fachinformationszentrum Karlsruhe Access Structures service.

### General photophysical measurements

All measurements were performed in standard quartz cuvettes (1 cm × 1 cm cross‐section) fitted with screw caps. UV/Visible absorption spectra were recorded using an Agilent 8453 diode array UV/Vis spectrophotometer. Emission and excitation spectra were recorded using an Edinburgh Instruments FLSP920 spectrometer equipped with a cooled red PMT detector from Hamamatsu (R13456‐P), and with double monochromators, operating in right‐angle geometry mode. A 450 W continuous xenon arc lamp (Xe900) was used as the excitation source and was focused on the sample. All solutions used in photophysical measurements had concentrations lower than 5×10^−6^ m to eliminate the chances of excimer formation during fluorescence measurements.

### Fluorescence quantum yield measurements

The fluorescence quantum yields were measured using a calibrated integrating sphere (150 mm inner diameter) from Edinburgh Instruments combined with the above FLSP920 spectrometer. The longest‐wavelength absorption maximum was chosen as the excitation wavelength, unless otherwise stated. The corresponding blank solvent was used as a reference for solution‐phase measurements and BaSO_4_ was used as reference for solid state measurements.

### Fluorescence lifetime measurements

Lifetimes were measured using a 315.8 nm emitting picosecond pulsed diode laser as the light source in the time‐correlated single‐photon counting (TCSPC) mode using the Edinburgh Instruments FLS920 spectrometer. Emission decay was detected with a 3 nm emission‐slit band‐width. A 5000 kHz (every 200 ns) pulse was generated. The time range was set to 200 ns, and 1024 channels were used. Instrument response functions (IRFs) were measured from the scatter of an aqueous suspension of Ludox at the same excitation wavelength. A reconvolution‐fit was performed using Software F900 version 7.2.1. The quality of the decay fits was assessed by the calculated values of the reduced χ^2^ and Durbin‐Watson parameters, and visual inspection of the weighted and autocorrelated residuals.

### Electrochemistry

Cyclic voltammetry measurements were carried out using a Gamry Instruments Reference 600 potentiostat. A standard arrangement of a three‐electrode cell configuration was set up using a platinum disk working electrode, a platinum wire counter electrode, and a silver wire, separated by a *Vycor* tip, as the reference electrode. Formal redox potentials are referenced to the ferrocene/ferrocenium ([Cp_2_Fe]^+/0^) redox couple by using ferrocene as an internal standard. Tetra‐*n*‐butylammonium hexafluorophosphate ([*n*Bu_4_N][PF_6_]) was employed as the supporting electrolyte. Compensation for resistive losses (*iR* drop) was employed for all measurements.

### Spectroelectrochemical measurements

Spectroelectrochemical experiments were carried out using an Agilent Cary 5000 spectrometer in combination with a designed sample compartment consisting of a cylindrical PTFE cell with an Infrasil® wedge window with an angle of 0.5°. An adjustable three‐in‐one electrode (6 mm platinum disc as working electrode, 1 mm platinum as counter electrode, and a pseudo reference electrode) was used. The potentials were adjusted with a Gamry 600 potentiostat. All experiments were measured at room temperature under an argon atmosphere.

### Theoretical computations

All DFT calculations were carried out using the Gaussian09‐D01 suite of programs.^[63]^ The geometries of all the compounds were optimised without symmetry constraints using the PBE0 functional[^64^, ^65^] including Grimme's dispersion correction with Becke‐Johnson damping (GD3BJ),[^66^, ^67^] and the LANL2DZ basis set augmented with polarisation functions for all atoms, except hydrogen (xyz coordinates of the optimised geometries are given in Table S4, Supporting Information). The nature of the stationary points after optimisation was checked by calculations of the harmonic vibrational frequencies to ensure that genuine minima were obtained. Time‐dependent density functional theory (TD‐DFT) calculations were performed at the same level of theory using the previously optimised geometries. For comparison, computations were also carried out using the CAM‐B3LYP functional^[68]^ (Tables S5 and S6, Supporting Information). A better agreement with the experimentally observed values was obtained with the PBE0 functional. Solvent effects were taken into account by means of the polarisable continuum model (PCM).^[69]^ The molecular orbitals and theoretical absorption spectra were drawn using the GaussView program.^[70]^

Periodic DFT calculations were performed on compound **2M** using the VASP code.^[71]^ The exchange‐correlation interaction was described within the generalised gradient approximation in the parametrisation of the Perdew‐Burke‐Ernzerhof functional.^[64]^ Projector‐augmented wave potentials were used for all atoms.^[72]^ Calculations were performed with a cut‐off energy of 530 eV. The electronic wave functions were sampled on dense densities in the irreducible BZ using the Monkhorst–Pack method.^[73]^ Geometry optimisation including cell parameters and atomic positions were carried out without any symmetry constraints (see 2M‐sol‐opt.mol file in the Supporting Information).

### Study of interactions with DNA

Stock solutions of **1M** and **2M** were prepared in DMSO (1 mm) and were diluted prior to use, whereby the DMSO content was always maintained at <0.1 %. The UV/Vis spectra were recorded on a Varian Cary 100 Bio spectrophotometer, fluorescence spectra were recorded on an Agilent Eclipse fluorimeter, and CD spectra were recorded on a JASCO J815 spectropolarimeter at 25.0 °C using appropriate quartz cuvettes of 1 cm path‐length. *Calf thymus* (ct)‐DNA was purchased from Aldrich, dissolved in sodium cacodylate buffer, *I*=0.05 m, pH 7.0, additionally sonicated and filtered through a 0.45 μm filter to obtain uniform rod‐like fragments of the B‐helical DNA.^[74]^ Polynucleotide concentration was determined spectroscopically, by measuring the absorbance at 260 nm for molar extinction coefficient 6600 m ^−1^ cm^−1^, whereby the concentration per nucleobase was obtained (traditionally referred as the concentration of phosphates).

For the study of interactions with DNA, the aqueous solutions of the compounds were buffered to pH 7.0 (sodium cacodylate buffer, *I*=0.05 m). Spectrophotometric titrations were performed by adding portions of DNA solution into the solution of the compound under investigation and CD experiments were performed by adding portions of stock solution of our compounds into the solution of DNA. In all titration experiments, dilution effects were taken into account.

Thermal melting curves for ds‐DNA and its complexes with **1M** and **2M** were determined as previously described,^[55]^ by following the absorption change at 260 nm as a function of temperature. The absorbance of the ligands was subtracted from every curve and the absorbance scale was normalised. The *T*_m_ values, the midpoints of the transition curves, were determined from the maximum of the first derivative and were checked graphically by the tangent method.[^55^, ^75^] The Δ*T*_m_ values were calculated by subtracting *T*_m_ values of the free nucleic acid from that of the complex. Every Δ*T*_m_ value reported was the average of at least two measurements. The error in Δ*T*_m_ can be ±0.5 °C.

## Conflict of interest

The authors declare no conflict of interest.

## Supporting information

As a service to our authors and readers, this journal provides supporting information supplied by the authors. Such materials are peer reviewed and may be re‐organized for online delivery, but are not copy‐edited or typeset. Technical support issues arising from supporting information (other than missing files) should be addressed to the authors.

Supplementary

## References

[1] T. M. Figueira-Duarte , K. Müllen , Chem. Rev. 2011, 111, 7260–7314. 21740071 10.1021/cr100428a

[2a] K. Kalyanasundaram , J. K. Thomas , J. Am. Chem. Soc. 1977, 99, 2039–2044;

[2b] A. Mateo-Alonso , Chem. Soc. Rev. 2014, 43, 6311–6324; 24841033 10.1039/c4cs00119b

[2c] W.-L. Jia , T. McCormick , Q.-D. Liu , H. Fukutani , M. Motala , R.-Y. Wang , Y. Tao , S. Wang , J. Mater. Chem. 2004, 14, 3344–3350;

[2d] M. M. Islam , Z. Hu , Q. Wang , C. Redshaw , X. Feng , Mater. Chem. Front. 2019, 3, 762–781;

[2e] Y. Gong , X. Zhan , Q. Li , Z. Li , Sci. China Chem. 2016, 59, 1623–1631;

[2f] H. Ju , K. Wang , J. Zhang , H. Geng , Z. Liu , G. Zhang , Y. Zhao , D. Zhang , Chem. Mater. 2017, 29, 3580–3588;

[2g] M. Liu , X. Gong , C. Zheng , D. Gao , Asian J. Org. Chem. 2019, 8, 722–730;

[2h] S. Zhang , X. Qiao , Y. Chen , Y. Wang , R. M. Edkins , Z. Liu , H. Li , Q. Fang , Org. Lett. 2014, 16, 342–345; 24392998 10.1021/ol402971n

[2i] D. Chercka , S.-J. Yoo , M. Baumgarten , J.-J. Kim , K. Müllen , J. Mater. Chem. C 2014, 2, 9083–9086;

[2j] H. Jung , S. Kang , H. Lee , Y.-J. Yu , J. Ho Jeong , J. Song , Y. Jeon , J. Park , ACS Appl. Mater. Interfaces 2018, 10, 30022–30028; 30145879 10.1021/acsami.8b09013

[2k] T. P. D. De Silva , S. G. Youm , G. G. Tamas , B. Yang , C.-H. Wang , F. R. Fronczek , G. Sahasrabudhe , S. Sterling , R. D. Quarels , P. K. Chhotaray , E. E. Nesterov , I. M. Warner , ACS Omega 2019, 4, 16867–16877; 31646233 10.1021/acsomega.9b01948 PMC6796915

[2l] J. Jayabharathi , S. Panimozhi , V. Thanikachalam , Sci. Rep. 2019, 9, 17555; 31772249 10.1038/s41598-019-54125-x PMC6879643

[2m] K. Oniwa , H. Kikuchi , H. Shimotani , S. Ikeda , N. Asao , Y. Yamamoto , K. Tanigaki , T. Jin , Chem. Commun. 2016, 52, 4800–4803. 10.1039/c6cc00948d 26960411

[3a] F. M. Winnik , Chem. Rev. 1993, 93, 587–614;

[3b] F. M. Winnik , M. A. Winnik , S. Tazuke , J. Phys. Chem. 1987, 91, 594–597;

[3c] J. Duhamel , Langmuir 2012, 28, 6527–6538; 22423596 10.1021/la2047646

[3d] T. Zhang , S. D. Taylor , M. Palmer , J. Duhamel , Biophys. J. 2016, 111, 1267–1277; 27653485 10.1016/j.bpj.2016.07.018 PMC5034303

[3e] M. Fowler , J. Duhamel , X. P. Qiu , E. Korchagina , F. M. Winnik , J. Polym. Sci. Part B 2018, 56, 308–318;

[3f] D. Kim , R. Amos , M. Gauthier , J. Duhamel , Langmuir 2018, 34, 8611–8621. 29936845 10.1021/acs.langmuir.8b01591

[4a] D. Sahoo , V. Narayanaswami , C. M. Kay , R. O. Ryan , Biochemistry 2000, 39, 6594–6601; 10828977 10.1021/bi992609m

[4b] P. Somerharju , Chem. Phys. Lipids 2002, 116, 57–74; 12093535 10.1016/s0009-3084(02)00020-8

[4c] N. I. Zahid , L. Ji , M. F. Khyasudeen , A. Friedrich , R. Hashim , T. B. Marder , O. K. Abou-Zied , Langmuir 2019, 35, 9584–9592. 31287700 10.1021/acs.langmuir.9b01767

[5a] M. E. Østergaard , P. J. Hrdlicka , Chem. Soc. Rev. 2011, 40, 5771–5788; 21487621 10.1039/c1cs15014f PMC3644995

[5b] C. Wu , C. Wang , L. Yan , C. J. Yang , J. Biomed. Nanotechnol. 2009, 5, 495–504. 20201423 10.1166/jbn.2009.1074

[6a] K. Yamana , H. Zako , K. Asazuma , R. Iwase , H. Nakano , A. Murakami , Angew. Chem. Int. Ed. 2001, 40, 1104–1106; 10.1002/1521-3773(20010316)40:6<1104::aid-anie11040>3.0.co;2-2 11268089

[6b] V. A. Korshun , D. A. Stetsenko , M. J. Gait , J. Chem. Soc. Perkin Trans. 1 2002, 1092–1104.

[7a] E. Kostenko , M. Dobrikov , D. Pyshnyi , V. Petyuk , N. Komarova , V. Vlassov , M. Zenkova , Nucleic Acids Res. 2001, 29, 3611–3620; 11522831 10.1093/nar/29.17.3611 PMC55892

[7b] A. Mahara , R. Iwase , T. Sakamoto , K. Yamana , T. Yamaoka , A. Murakami , Angew. Chem. Int. Ed. 2002, 41, 3648–3650; 10.1002/1521-3773(20021004)41:19<3648::AID-ANIE3648>3.0.CO;2-Y 12370918

[7c] I. V. Astakhova , D. Lindegaard , V. A. Korshun , J. Wengel , Chem. Commun. 2010, 46, 8362–8364. 10.1039/c0cc03026k 20922231

[8a] U. B. Christensen , E. B. Pedersen , Nucleic Acids Res. 2002, 30, 4918–4925; 12433995 10.1093/nar/gkf624 PMC137172

[8b] V. V. Filichev , E. B. Pedersen , Org. Biomol. Chem. 2003, 1, 100–103. 12929395 10.1039/b210335b

[9a] K. Gröger , D. Baretić , I. Piantanida , M. Marjanović , M. Kralj , M. Grabar , S. Tomić , C. Schmuck , Org. Biomol. Chem. 2011, 9, 198–209; 21076779 10.1039/c0ob00103a

[9b] I. V. Astakhova , A. D. Malakhov , I. A. Stepanova , A. V. Ustinov , S. L. Bondarev , A. S. Paramonov , V. A. Korshun , Bioconjugate Chem. 2007, 18, 1972–1980. 10.1021/bc700280h 17896811

[10a] L. Hernandez-Folgado , C. Schmuck , S. Tomić , I. Piantanida , Bioorg. Med. Chem. Lett. 2008, 18, 2977–2981; 18395444 10.1016/j.bmcl.2008.03.060

[10b] L. Hernandez-Folgado , D. Baretić , I. Piantanida , M. Marjanović , M. Kralj , T. Rehm , C. Schmuck , Chem. Eur. J. 2010, 16, 3036–3056; 20119980 10.1002/chem.200901999

[10c] J. Wu , Y. Zou , C. Li , W. Sicking , I. Piantanida , T. Yi , C. Schmuck , J. Am. Chem. Soc. 2012, 134, 1958–1961; 22242714 10.1021/ja2103845

[10d] F. Ma , W.-J. Liu , Z. Qianyi , C.-Y. Zhang , Chem. Commun. 2017, 53, 10596–10599. 10.1039/c7cc06290g 28900650

[11a] W. M. Vaughn , G. Weber , Biochemistry 1970, 9, 464–473; 5461215 10.1021/bi00805a003

[11b] O. Oter , A.-C. Ribou , J. Fluoresc. 2009, 19, 389–397. 18931890 10.1007/s10895-008-0425-z

[12a] B. Bodenant , F. Fages , M.-H. Delville , J. Am. Chem. Soc. 1998, 120, 7511–7519;

[12b] B. Valeur , Coord. Chem. Rev. 2000, 205, 3–40;

[12c] J.-S. Yang , C.-S. Lin , C.-Y. Hwang , Org. Lett. 2001, 3, 889–892; 11263908 10.1021/ol015524y

[12d] R.-H. Yang , W.-H. Chan , A. W. M. Lee , P.-F. Xia , H.-K. Zhang , K.’A. Li , J. Am. Chem. Soc. 2003, 125, 2884–2885; 12617649 10.1021/ja029253d

[12e] N. Kumari , N. Dey , S. Jha , S. Bhattacharya , ACS Appl. Mater. Interfaces 2013, 5, 2438–2445. 23427918 10.1021/am400063k

[13] J. B. Birks , M. D. Lumb , I. H. Munro , Proc. R. Soc. London Ser. A 1964, 280, 289–297.

[14] R. H. Templer , S. J. Castle , A. R. Curran , G. Rumbles , D. R. Klug , Faraday Discuss. 1999, 111, 41–53. 10.1039/a806472e 10822599

[15] M. R. Pokhrel , S. H. Bossmann , J. Phys. Chem. B 2000, 104, 2215–2223.

[16a] A. Ueno , I. Suzuki , T. Osa , Anal. Chem. 1990, 62, 2461–2466;

[16b] H. Ikeda , M. Nakamura , N. Ise , N. Oguma , A. Nakamura , T. Ikeda , F. Toda , A. Ueno , J. Am. Chem. Soc. 1996, 118, 10980–10988;

[16c] Y. Fujiwara , Y. Amao , Sens. Actuators B 2003, 89, 58–61;

[16d] T. Aoyagi , H. Ikeda , A. Ueno , Bull. Chem. Soc. Jpn. 2001, 74, 157–164.

[17a] T. Iwasaki , S. Murakami , Y. Takeda , N. Tohnai , N. Kambe , Chem. Asian J. 2020, 15, 1349–1354; 32103620 10.1002/asia.202000138

[17b] M. Perry , C. Carra , M. N. Chrétien , J. C. Scaiano , J. Phys. Chem. A 2007, 111, 4884–4889; 17516633 10.1021/jp0702797

[17c] J. C. Collings , K. P. Roscoe , R. Ll. Thomas , A. S. Batsanov , L. M. Stimson , J. A. K. Howard , T. B. Marder , New J. Chem. 2001, 25, 1410–1417;

[17d] J. C. Collings , K. P. Roscoe , E. G. Robins , A. S. Batsanov , L. M. Stimson , J. A. K. Howard , S. J. Clark , T. B. Marder , New J. Chem. 2002, 26, 1740–1746;

[17e] A. F. M. Kilbinger , R. H. Grubbs , Angew. Chem. Int. Ed. 2002, 41, 1563–1566; 10.1002/1521-3773(20020503)41:9<1563::aid-anie1563>3.0.co;2-7 19750666

[18a] M. Eddaoudi , J. Kim , N. Rosi , D. Vodak , J. Wachter , M. O'Keeffe , O. M. Yaghi , Science 2002, 295, 469–472; 11799235 10.1126/science.1067208

[18b] N. L. Rosi , J. Kim , M. Eddaoudi , B. Chen , M. O'Keeffe , O. M. Yaghi , J. Am. Chem. Soc. 2005, 127, 1504–1518; 15686384 10.1021/ja045123o

[18c] C. A. Williams , A. J. Blake , C. Wilson , P. Hubberstey , M. Schröder , Cryst. Growth Des. 2008, 8, 911–922.

[19a] T. Sick , J. M. Rotter , S. Reuter , S. Kandambeth , N. N. Bach , M. Döblinger , J. Merz , T. Clark , T. B. Marder , T. Bein , D. D. Medina , J. Am. Chem. Soc. 2019, 141, 12570–12581; 31251878 10.1021/jacs.9b02800

[19b] S. Ghosh , Y. Tsutsui , K. Suzuki , H. Kaji , K. Honjo , T. Uemura , S. Seki , Mol. Syst. Des. Eng. 2019, 4, 325–331.

[20] Q. Lu , G. K. Kole , A. Friedrich , K. Müller-Buschbaum , Z. Liu , X. Yu , T. B. Marder , J. Org. Chem. 2020, 85, 4256–4266. 32129624 10.1021/acs.joc.9b03421

[21] J. Merz , J. Fink , A. Friedrich , I. Krummenacher , H. H. Al Mamari , S. Lorenzen , M. Haehnel , A. Eichhorn , M. Moos , M. Holzapfel , H. Braunschweig , C. Lambert , A. Steffen , L. Ji , T. B. Marder , Chem. Eur. J. 2017, 23, 13164–13180. 28718975 10.1002/chem.201702594

[22a] H. Vollmann , H. Becker , M. Corell , H. Streeck , Eur. J. Org. Chem. 1937, 1–159;

[22b] X. Feng , J.-Y. Hu , C. Redshaw , T. Yamato , Chem. Eur. J. 2016, 22, 11898–11916. 27388023 10.1002/chem.201600465

[23a] D. N. Coventry , A. S. Batsanov , A. E. Goeta , J. A. K. Howard , T. B. Marder , R. N. Perutz , Chem. Commun. 2005, 2172–2174; 10.1039/b501778e 15846437

[23b] I. A. I. Mkhalid , J. H. Barnard , T. B. Marder , J. M. Murphy , J. F. Hartwig , Chem. Rev. 2010, 110, 890–931; 20028025 10.1021/cr900206p

[23c] A. G. Crawford , Z. Liu , I. A. I. Mkhalid , M.-H. Thibault , N. Schwarz , G. Alcaraz , A. Steffen , J. C. Collings , A. S. Batsanov , J. A. K. Howard , T. B. Marder , Chem. Eur. J. 2012, 18, 5022–5035. 22415854 10.1002/chem.201103774

[24] A. G. Crawford , A. D. Dwyer , Z. Liu , A. Steffen , A. Beeby , L.-O. Pålsson , D. J. Tozer , T. B. Marder , J. Am. Chem. Soc. 2011, 133, 13349–13362. 21751803 10.1021/ja2006862

[25a] L. Ji , A. Lorbach , R. M. Edkins , T. B. Marder , J. Org. Chem. 2015, 80, 5658–5665; 25927248 10.1021/acs.joc.5b00618

[25b] L. Ji , R. M. Edkins , A. Lorbach , I. Krummenacher , C. Brückner , A. Eichhorn , H. Braunschweig , B. Engels , P. J. Low , T. B. Marder , J. Am. Chem. Soc. 2015, 137, 6750–6753; 25948415 10.1021/jacs.5b03805

[25c] J. Merz , M. Dietz , Y. Vonhausen , F. Wöber , A. Friedrich , D. Sieh , I. Krummenacher , H. Braunschweig , M. Moos , M. Holzapfel , C. Lambert , T. B. Marder , Chem. Eur. J. 2020, 26, 438–453. 31593316 10.1002/chem.201904219 PMC6973242

[26a] R. Kurata , A. Ito , M. Gon , K. Tanaka , Y. Chujo , J. Org. Chem. 2017, 82, 5111–5121; 28481543 10.1021/acs.joc.7b00315

[26b] R. Kurata , K. Tanaka , A. Ito , J. Org. Chem. 2016, 81, 137–145. 26690970 10.1021/acs.joc.5b02425

[27a] R. M. Moriarty , P. Dansette , D. M. Jerina , Tetrahedron Lett. 1975, 16, 2557–2560;

[27b] L. Zöphel , V. Enkelmann , K. Müllen , Org. Lett. 2013, 15, 804–807; 23387523 10.1021/ol303476g

[27c] L. Zöphel , D. Beckmann , V. Enkelmann , D. Chercka , R. Rieger , K. Müllen , Chem. Commun. 2011, 47, 6960–6962. 10.1039/c1cc11827g 21594241

[28] K. Mochida , K. Kawasumi , Y. Segawa , K. Itami , J. Am. Chem. Soc. 2011, 133, 10716–10719. 21699210 10.1021/ja202975w

[29a] Z. Liu , Y. Wang , Y. Chen , J. Liu , Q. Fang , C. Kleeberg , T. B. Marder , J. Org. Chem. 2012, 77, 7124–7128; 22816387 10.1021/jo301293w

[29b] L. Ji , I. Krummenacher , A. Friedrich , A. Lorbach , M. Haehnel , K. Edkins , H. Braunschweig , T. B. Marder , J. Org. Chem. 2018, 83, 3599–3606. 29480011 10.1021/acs.joc.7b03227

[30a] J.-Y. Hu , M. Era , M. R. J. Elsegood , T. Yamato , Eur. J. Org. Chem. 2010, 72–79;

[30b] S. Sasaki , S. Suzuki , K. Igawa , K. Morokuma , G.-i. Konishi , J. Org. Chem. 2017, 82, 6865–6873. 28656764 10.1021/acs.joc.7b00996

[31a] H. Weidel , M. Russo , Monatsh. Chem. 1882, 3, 850–885;

[31b] L. Michaelis , E. S. Hill , J. Gen. Physiol. 1933, 16, 859–873; 19872744 10.1085/jgp.16.6.859 PMC2141252

[31c] C. L. Bird , A. T. Kuhn , Chem. Soc. Rev. 1981, 10, 49–82;

[31d] T. M. Bockman , J. K. Kochi , J. Org. Chem. 1990, 55, 4127–4135;

[31e] K. Takahashi , T. Nihira , K. Akiyama , Y. Ikegami , E. Fukuyoc , J. Chem. Soc. Chem. Commun. 1992, 620–622;

[31f] J. W. Happ , J. A. Ferguson , D. G. Whitten , J. Org. Chem. 1972, 37, 1485–1491.

[32a] L. Striepe , T. Baumgartner , Chem. Eur. J. 2017, 23, 16924–16940; 28815887 10.1002/chem.201703348

[32b] J. Ding , C. Zheng , L. Wang , C. Lu , B. Zhang , Y. Chen , M. Li , G. Zhai , X. Zhuang , J. Mater. Chem. A 2019, 7, 23337–23360;

[32c] K. Madasamy , D. Velayutham , V. Suryanarayanan , M. Kathiresan , K.-C. Ho , J. Mater. Chem. C 2019, 7, 4622–4637;

[32d] A. N. Woodward , J. M. Kolesar , S. R. Hall , N.-A. Saleh , D. S. Jones , M. G. Walter , J. Am. Chem. Soc. 2017, 139, 8467–8473; 28481091 10.1021/jacs.7b01005

[32e] J.-W. Kim , J.-M. Myoung , Adv. Funct. Mater. 2019, 29, 1808911;

[32f] T. Kakibe , H. Ohno , J. Mater. Chem. 2009, 19, 4960–4964;

[32g] S.-Y. Kao , Y. Kawahara , S.i. Nakatsuji , K.-C. Ho , J. Mater. Chem. C 2015, 3, 3266–3272.

[33a] D. Corr , S. N. Rao , N. Stobie , M. Kinsella , US Patent, 2009, US 7,567,371 B2;

[33b] S. Archambeau , C. Biver , F. Berit-Debat , S. Aiken , C. D. Gabbutt , B. M. Heron , T. D. Roadbent , European Patent Application, 2017, EP 3 115 433 A1;

[33c] W. M. Kline , R. G. Lorenzini , G. A. Sotzing , Color. Technol. 2014, 130, 73–80;

[33d] C. J. Schoot , J. J. Ponjee , H. T. van Dam , R. A. van Doorn , P. T. Bolwijn , Appl. Phys. Lett. 1973, 23, 64–65;

[33e] R. J. Mortimer , D. R. Rosseinsky , P. M. S. Monk , Electrochromic Materials and Devices, Wiley-VCH, Weinheim, 2015.

[34a] M. Nanasawa , M. Miwa , M. Hirai , T. Kuwabara , J. Org. Chem. 2000, 65, 593–595; 10813978 10.1021/jo990911v

[34b] X. Zhang , E. L. Clennan , N. Arulsamy , R. Weber , J. Weber , J. Org. Chem. 2016, 81, 5474–5486; 27284956 10.1021/acs.joc.6b00835

[34c] W.-Q. Kan , S.-Z. Wen , Y.-C. He , C.-Y. Xu , Inorg. Chem. 2017, 56, 14926–14935. 29200269 10.1021/acs.inorgchem.7b02206

[35a] P. L. Anelli , N. Spencer , J. F. Stoddart , J. Am. Chem. Soc. 1991, 113, 5131–5133; 27715028 10.1021/ja00013a096

[35b] A. C. Benniston , A. Harriman , Angew. Chem. Int. Ed. Engl. 1993, 32, 1459–1461;

[35c] R. A. Bissell , E. Cordova , A. E. Kaifer , J. F. Stoddart , Nature 1994, 369, 133–137;

[35d] C. Cheng , P. R. McGonigal , S. T. Schneebeli , H. Li , N. A. Vermeulen , C. Ke , J. F. Stoddart , Nat. Nanotechnol. 2015, 10, 547–553; 25984834 10.1038/nnano.2015.96

[35e] C. Kahlfuss , E. Métay , M.-C. Duclos , M. Lemaire , A. Milet , E. Saint-Aman , C. Bucher , Chem. Eur. J. 2015, 21, 2090–2106. 25450858 10.1002/chem.201405157

[36a] T. Janoschka , N. Martin , U. Martin , C. Friebe , S. Morgenstern , H. Hiller , M. D. Hager , U. S. Schubert , Nature 2015, 527, 78–83; 26503039 10.1038/nature15746

[36b] T. Liu , X. Wie , Z. Nie , V. Sprenkle , W. Wang , Adv. Energy Mater. 2016, 6, 1501449;

[36c] C. Debruler , B. Hu , J. Moss , J. Luo , T. L. Liu , ACS Energy Lett. 2018, 3, 663–668;

[36d] J. Luo , B. Hu , C. Debruler , T. L. Liu , Angew. Chem. Int. Ed. 2018, 57, 231–235; 10.1002/anie.201710517 29181865

[37a] L. A. Summers , The Bipyridinium Herbicides, 1980, Academic Press, London;

[37b] H. Fischer , L. A. Summers , Tetrahedron 1976, 32, 615–618;

[37c] J. E. Rockley , L. A. Summers , Aust. J. Chem. 1980, 33, 1397–1400.

[38a] A. C. Revkin , Sci. Dig. 1983, 91, 36–38;10298869

[38b] F. Kamel , Science 2013, 341, 722–723; 23950519 10.1126/science.1243619

[38c] W.-T. Tsai , Toxicol. Environ. Chem. 2013, 95, 197–206;

[38d] R. J. Dinis-Oliveira , J. A. Duarte , A. Sánchez-Navarro , F. Remião , M. L. Bastos , F. Carvalho , Crit. Rev. Toxicol. 2008, 38, 13–71. 18161502 10.1080/10408440701669959

[39a] P. M. S. Monk , The viologens: physicochemical properties, synthesis, and applications of the salts of 4,4′-bipyridine, Wiley, Chichester, 1998;

[39b] W. W. Porter III , T. P. Vaid , J. Org. Chem. 2005, 70, 5028–5035; 15960502 10.1021/jo050328g

[39c] J. Petersson , L. Hammarström , J. Phys. Chem. B 2015, 119, 7531–7540; 25766332 10.1021/jp5113119

[39d] N. Li , Y. Y. Liu , Y. Li , J. B. Zhuang , R. R. Cui , Q. Gong , N. Zhao , B. Z. Tang , ACS Appl. Mater. Interfaces 2018, 10, 24249–24257; 29939714 10.1021/acsami.8b04113

[39e] R.-T. Wang , G.-H. Lee , C. K. Lai , J. Mater. Chem. C 2018, 6, 9430–9444.

[40] A. C. Benniston , J. Hagon , X. He , S. Yang , R. W. Harrington , Org. Lett. 2012, 14, 506–509. 22220836 10.1021/ol203099h

[41a] S. Durben , T. Baumgartner , Angew. Chem. Int. Ed. 2011, 50, 7948–7952; 10.1002/anie.201102453 21732507

[41b] M. Stolar , J. Borau-Garcia , M. Toonen , T. Baumgartner , J. Am. Chem. Soc. 2015, 137, 3366–3371; 25730100 10.1021/ja513258j

[41c] T. W. Greulich , E. Yamaguchi , C. Doerenkamp , M. Lebbesmeyer , C. G. Daniliuc , A. Fukazawa , H. Eckert , S. Yamaguchi , A. Studer , Chem. Eur. J. 2017, 23, 6029–6033. 28074501 10.1002/chem.201605272

[42] K. Murakami , J. Ohshita , S. Inagi , I. Tomita , Electrochemistry 2015, 83, 605–608.

[43a] M. E. Alberto , B. C. De Simone , S. Cospito , D. Imbardelli , L. Veltri , G. Chidichimo , N. Russo , Chem. Phys. Lett. 2012, 552, 141–145;

[43b] A. Beneduci , S. Cospito , A. Crispini , B. Gabriele , F. P. Nicoletta , L. Veltria , G. Chidichimo , J. Mater. Chem. C 2013, 1, 2233–2240;

[43c] S. Hünig , A. Langels , M. Schmittel , H. Wenner , I. F. Perepichka , K. Peters , Eur. J. Org. Chem. 2001, 1393–1399.

[44a] W. W. Porter III , T. P. Vaid , A. L. Rheingold , J. Am. Chem. Soc. 2005, 127, 16559–16566; 16305245 10.1021/ja053084q

[44b] T. S. Arrhenius , M. Blanchard-Desce , M. Dvolaitzky , J.-M. Lehn , J. Malthete , Proc. Natl. Acad. Sci. USA 1986, 83, 5355–5359; 16593731 10.1073/pnas.83.15.5355 PMC386284

[44c] Y.-S. Su , C.-F. Chen , Org. Lett. 2010, 12, 1888–1891. 20337447 10.1021/ol100589u

[45a] U. Giovanella , E. Cariati , E. Lucenti , M. Pasini , F. Galeotti , C. Botta , ChemPhysChem 2017, 18, 2157–2161; 28240411 10.1002/cphc.201700185

[45b] E. Cariati , C. Botta , S. G. Danelli , A. Forni , A. Giaretta , U. Giovanella , E. Lucenti , D. Marinotto , S. Righetto , R. Ugo , Chem. Commun. 2014, 50, 14225–14228. 10.1039/c4cc05891g 25283160

[46a] A. P. Monkman , L.-O. Pålsson , R. W. T. Higgins , C. Wang , M. R. Bryce , A. S. Batsanov , J. A. K. Howard , J. Am. Chem. Soc. 2002, 124, 6049–6055; 12022839 10.1021/ja012409+

[46b] T. M. Fasina , J. C. Collings , D. P. Lydon , D. Albesa-Jove , A. S. Batsanov , J. A. K. Howard , P. Nguyen , M. Bruce , A. J. Scott , W. Clegg , S. W. Watt , C. Viney , T. B. Marder , J. Mater. Chem. 2004, 14, 2395–2404;

[46c] X. Chen , X. Y. Shen , E. Guan , Y. Liu , A. Qin , J. Z. Sun , B. Z. Tang , Chem. Commun. 2013, 49, 1503–1505; 10.1039/c2cc38246f 23325316

[46d] S. Chen , M. Zhao , J. Su , Q. Zhang , X. Tian , S. Li , H. Zhou , J. Wu , Y. Tian , Dyes Pigm. 2017, 136, 807–816.

[47a] J. Kawamata , Y. Suzuki , H. Moritomo , S. Fujiki , *PCT Int. Appl*. 2016, WO 2016143335 A1 20160915 and

[47b] A. D. Shukla , D. Strawser , A. C. B. Lucassen , D. Freeman , H. Cohen , D. A. Jose , A. Das , G. Evmenenko , P. Dutta , M. E. van der Boom , J. Phys. Chem. B 2004, 108, 17505–17511;

[47c] Z. Ding , M. Tian , L. Guo , Z.-q. Liu , X. Yu , Sens. Actuators B 2018, 276, 331–339;

[47d] T. Hirsch , H. Port , H. C. Wolf , B. Miehlich , F. Effenberger , J. Phys. Chem. B 1997, 101, 4525–4535;

[47e] Y. Shi , J. Liu , M. Li , J. Zheng , C. Xu , Electrochim. Acta 2018, 285, 415–423;

[47f] B.-D. Ge , Q. Wei , A.-H. Sun , C.-Y. Lin , X.-F. Duan , J.-H. Li , G.-M. Wang , Chem. Asian J. 2019, 14, 2086–2090; 30968564 10.1002/asia.201900392

[47g] W. Fudickar , T. Linker , Angew. Chem. Int. Ed. 2018, 57, 12971–12975; 10.1002/anie.201806881 30070421

[47h] R. Zhang , G. Niu , X. Li , L. Guo , H. Zhang , R. Yang , Y. Chen , X. Yu , B. Z. Tang , Chem. Sci. 2019, 10, 1994–2000; 30881628 10.1039/c8sc05119d PMC6383331

[47i] D. Li , W. Hu , J. Wang , Q. Zhang , X.-M. Cao , X. Ma , H. Tian , Chem. Sci. 2018, 9, 5709–5715. 30079179 10.1039/c8sc01915k PMC6050594

[48a] Y. Niko , H. Moritomo , H. Sugihara , Y. Suzuki , J. Kawamata , G.-i. Konishi , J. Mater. Chem. B 2015, 3, 184–190; 32261938 10.1039/c4tb01404a

[48b] C. S. Abeywickrama , K. J. Wijesinghe , R. V. Stahelin , Y. Pang , Chem. Commun. 2017, 53, 5886–5889. 10.1039/c7cc03417b 28509921

[49a] S. Hagiwara , Y. Ishida , D. Miasui , T. Shimada , S. Takagi , Clay Sci. 2013, 17, 7–10;

[49b] S. Hagiwara , Y. Ishida , D. Masui , T. Shimada , S. Takagi , Tetrahedron Lett. 2012, 53, 5800–5802;

[49c] K. Sato , K. Matsubara , S. Hagiwara , K. Saito , M. Yagi , S. Takagi , T. Yui , Langmuir 2015, 31, 27–31. 25540843 10.1021/la504597t

[50] J. Michl , E. W. Thulstrup , Spectroscopy with Polarized Light, VCH, Weinheim, 1986, p 368.

[51a] R. Englman , J. Jortner , Mol. Phys. 1970, 18, 145–164;

[51b] K. F. Freed , J. Jortner , J. Chem. Phys. 1970, 52, 6272–6291;

[51c] M. Bixon , J. Jortner , J. Cortes , H. Heitele , M. E. Michel-Beyerle , J. Phys. Chem. 1994, 98, 7289–7299.

[52] J. C. Bachman , R. Kavian , D. J. Graham , D. Y. Kim , S. Noda , D. G. Nocera , Y. Shao-Horn , S. W. Lee , Nat. Commun. 2015, 6, 7040. 25943905 10.1038/ncomms8040 PMC4432658

[53a] A. Heckmann , C. Lambert , Angew. Chem. Int. Ed. 2012, 51, 326–392; 10.1002/anie.201100944 22095940

[53b] C. Lambert , G. Nöll , Synth. Met. 2003, 139, 57–62.

[54a] N. G. Connelly , W. E. Geiger , Chem. Rev. 1996, 96, 877–910; 11848774 10.1021/cr940053x

[54b] V. V. Pavlishchuka , A. W. Addison , Inorg. Chim. Acta 2000, 298, 97–102.

[55] J.-L. Mergny , L. Lacroix , Oligonucleotides 2003, 13, 515–537. 15025917 10.1089/154545703322860825

[56] M. Radić-Stojković , P. Piotrowski , C. Schmuck , I. Piantanida , Org. Biomol. Chem. 2015, 13, 1629–1633. 25502619 10.1039/c4ob02169j

[57a] G. Scatchard , Ann. NY Acad. Sci. 1949, 51, 660–672;

[57b] J. D. McGhee , P. H. V. Hippel , J. Mol. Biol. 1974, 86, 469–489. 4416620 10.1016/0022-2836(74)90031-x

[58a] M. Eriksson , B. Nordén in Methods in Enzymology, Vol. 340 (Eds.: J. B. Chaires , M. J. Waring ), Academic Press, San Diego 2001, pp. 68–98; 10.1016/s0076-6879(01)40418-6 11494876

[58b] T. Šmidlehner , I. Piantanida , G. Pescitelli , Beilstein J. Org. Chem. 2018, 14, 84–105. 29441133 10.3762/bjoc.14.5 PMC5789433

[59] R. Uson , L. A. Oro , J. A. Cabeza , H. E. Bryndza , M. P. Stepro in Inorg. Synth., Vol. 23, (Ed.: S. Kirschner ), New York, 1985, pp. 126–130.

[60a] G. M. Sheldrick , Acta Crystallogr. Sect. A 2008, 64, 112–122; 18156677 10.1107/S0108767307043930

[60b] G. M. Sheldrick , Acta Crystallogr. Sect. A 2015, 71, 3–8; 10.1107/S2053273314026370 PMC4283466 25537383

[60c] C. B. Hübschle , G. M. Sheldrick , B. Dittrich , J. Appl. Crystallogr. 2011, 44, 1281–1284. 22477785 10.1107/S0021889811043202 PMC3246833

[61] M. J. Turner, J. J. McKinnon, S. K. Wolff, D. J. Grimwood, P. R. Spackman, D. Jayatilaka, M. A. Spackman, CrystalExplorer17, University of Western Australia, **2017**.

[62] C. F. Macrae , I. J. Bruno , J. A. Chisholm , P. R. Edgington , P. McCabe , E. Pidcock , L. Rodriguez-Monge , R. Taylor , J. van de Streek , P. A. Wood , J. Appl. Crystallogr. 2008, 41, 466–470.

[63] Gaussian 09, Revision D. 01, M. J. Frisch, G. W. Trucks, H. B. Schlegel, G. E. Scuseria, M. A. Robb, J. R. Cheeseman, G. Scalmani, V. Barone, B. Mennucci, G. A. Petersson, H. Nakatsuji, M. Caricato, X. Li, H. P. Hratchian, A. F. Izmaylov, J. Bloino, G. Zheng, J. L. Sonnenberg, M. Hada, M. Ehara, K. Toyota, R. Fukuda, J. Hasegawa, M. Ishida, T. Nakajima, Y. Honda, O. Kitao, H. Nakai, T. Vreven, J. A. Montgomery, Jr., J. E. Peralta, F. Ogliaro, M. Bearpark, J. J. Heyd, E. Brothers, K. N. Kudin, V. N. Staroverov, R. Kobayashi, J. Normand, K. Raghavachari, A. Rendell, J. C. Burant, S. S. Iyengar, J. Tomasi, M. Cossi, N. Rega, J. M. Millam, M. Klene, J. E. Knox, J. B. Cross, V. Bakken, C. Adamo, J. Jaramillo, R. Gomperts, R. E. Stratmann, O. Yazyev, A. J. Austin, R. Cammi, C. Pomelli, J. W. Ochterski, R. L. Martin, K. Morokuma, V. G. Zakrzewski, G. A. Voth, P. Salvador, J. J. Dannenberg, S. Dapprich, A. D. Daniels, Ö. Farkas, J. B. Foresman, J. V. Ortiz, J. Cioslowski, D. J. Fox, Gaussian Inc., Pittsburgh, PA, **2009**.

[64a] J. P. Perdew , K. Burke , M. Ernzerhof , Phys. Rev. Lett. 1996, 77, 3865–3868; 10062328 10.1103/PhysRevLett.77.3865

[64b] J. P. Perdew , M. Ernzerhof , K. J. Burke , Chem. Phys. 1996, 105, 9982–9985.

[65] C. Adamo , V. Barone , J. Chem. Phys. 1999, 110, 6158–6169.

[66] S. Grimme , J. Antony , S. Ehrlich , H. J. Krieg , Chem. Phys. 2010, 132, 154104–154123. 10.1063/1.3382344 20423165

[67] S. Grimme , S. Ehrlich , L. J. Goerigk , Comput. Chem. 2011, 32, 1456–1465. 10.1002/jcc.21759 21370243

[68] T. Yanai , D. P. Tew , N. C. Handy , Chem. Phys. Lett. 2004, 393, 51–57.

[69] J. Tomasi , B. Mennucci , R. Cammi , Chem. Rev. 2005, 105, 2999–3093. 16092826 10.1021/cr9904009

[70] R. Dennington, T. Keith, J. Millam, GaussView, Version5; Semichem Inc.: Shawnee Mission, KS, **2009**.

[71] G. Kresse , J. Furthmüller , Phys. Rev. B 1996, 54, 11169–11186. http://www.vasp.at. 10.1103/physrevb.54.11169 9984901

[72] P. E. Blöchl , Phys. Rev. B 1994, 50, 17953–17979. 10.1103/physrevb.50.17953 9976227

[73] H. J. Monkhorst , J. D. Pack , Phys. Rev. B 1976, 13, 5188–5192.

[74] J. B. Chaires , N. Dattagupta , D. M. Crothers , Biochemistry 1982, 21, 3933–3940. 7126524 10.1021/bi00260a005

[75] I. Stolić , K. Mišković , A. Magdaleno , A. M. Silber , I. Piantanida , M. Bajić , L. Glavaš-Obrovac , Bioorg. Med. Chem. 2009, 17, 2544–2554. 19231203 10.1016/j.bmc.2009.01.071

