# Supplementary material for: 2‐ and 2,7‐Substituted para‐N‐Methylpyridinium Pyrenes: Syntheses, Molecular and Electronic Structures, Photophysical, Electrochemical, and Spectroelectrochemical Properties and Binding to Double‐Stranded (ds) DNA
Source: Chemistry. 2021 Jan 7;27(8):2837–53. doi: 10.1002/chem.202004748 (PMC7898908; doi:10.1002/chem.202004748)
Supplement: Supplementary file 1 — Supplementary [file CHEM-27-2837-s001.pdf]

Chemistry–A European Journal

Supporting Information

2- and 2,7-Substituted *para*-*N*-Methylpyridinium Pyrenes: Syntheses, Molecular and Electronic Structures, Photophysical, Electrochemical, and Spectroelectrochemical Properties and Binding to Double-Stranded (ds) DNA

Goutam Kumar Kole,[a, b] Julia Merz,[a] Anissa Amar,[c] Bruno Fontaine,[d] Abdou Boucekkine,[d] Jörn Nitsch,[a] Sabine Lorenzen,[a] Alexandra Friedrich,[a] Ivo Krummenacher,[a] Marta Košćak,[e] Holger Braunschweig,[a] Ivo Piantanida,*[e] Jean-François Halet,*[d] Klaus Müller-Buschbaum,*[f] and Todd B. Marder*[a]

Table of Contents

NMR spectra	S2 – S7
Crystallographic table	S8
Additional diagrams for single crystal X-ray structures	S9 – S15
Powder X-ray diffraction studies	S16
Additional absorption, excitation and emission spectra	S17 – S18
Spectroelectrochemical measurements	S19 – S20
Theoretically optimized and experimentally determined structural parameters	S21
Coordinates of the optimized geometries	S21 – S24
Calculated electronic transitions	S25
Calculated and experimentally observed absorption and emission wavelengths	S26 – S27
Molecular orbital energy diagrams	S28
Study of interactions with ds-DNA	S29 – S35
Reference	S36

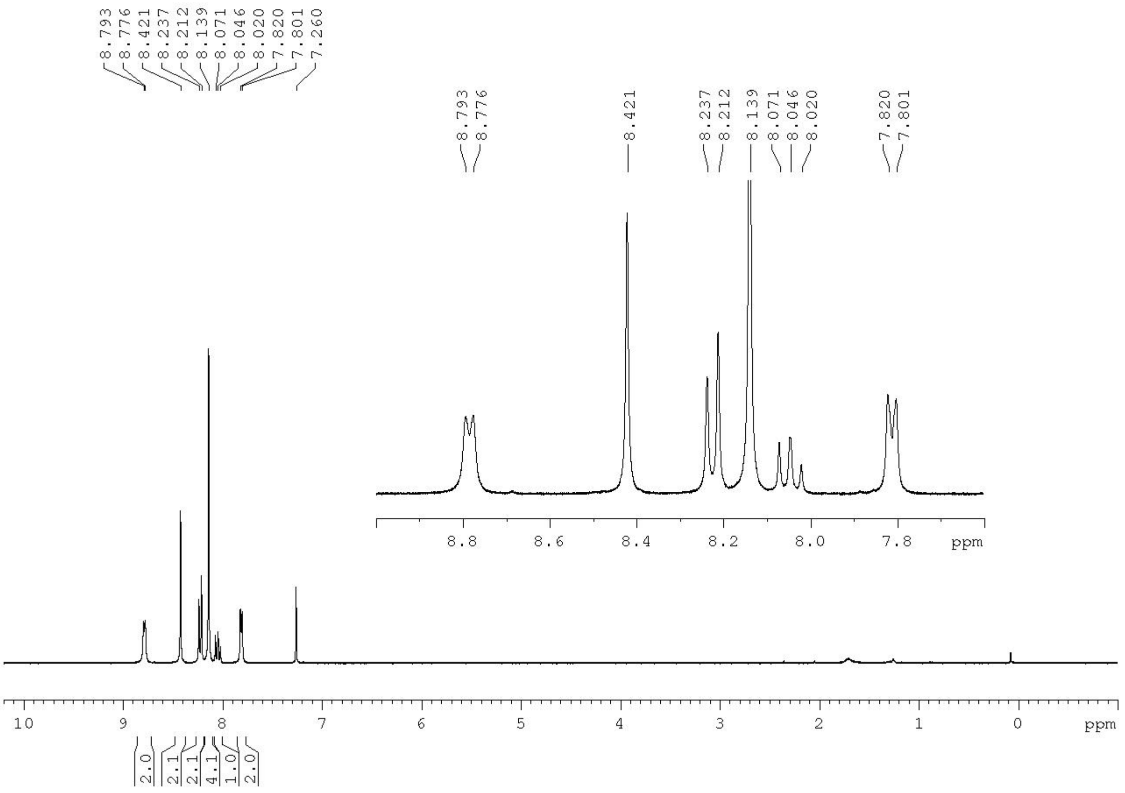

Figure S1: ^{1}H NMR (300 MHz, $CDCl_3$) spectrum of **1**.

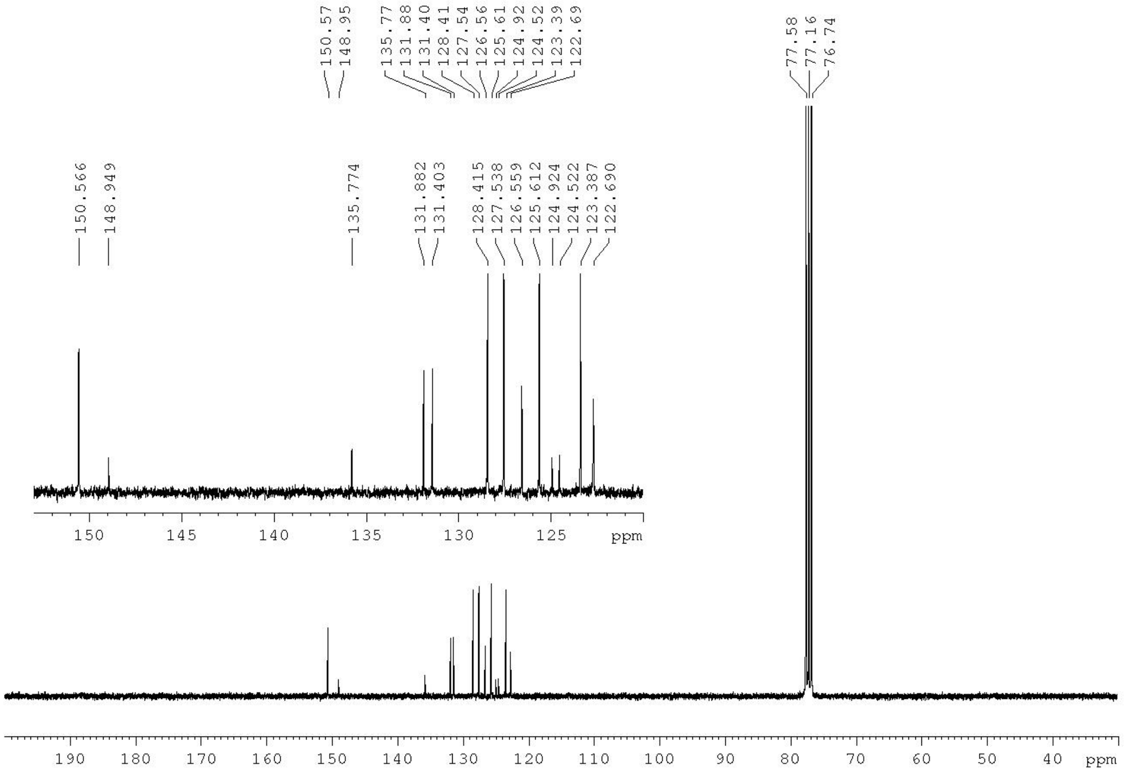

Figure S2: $^{13}C\{^{1}H\}$ NMR (75 MHz, $CDCl_3$) spectrum of **1**.

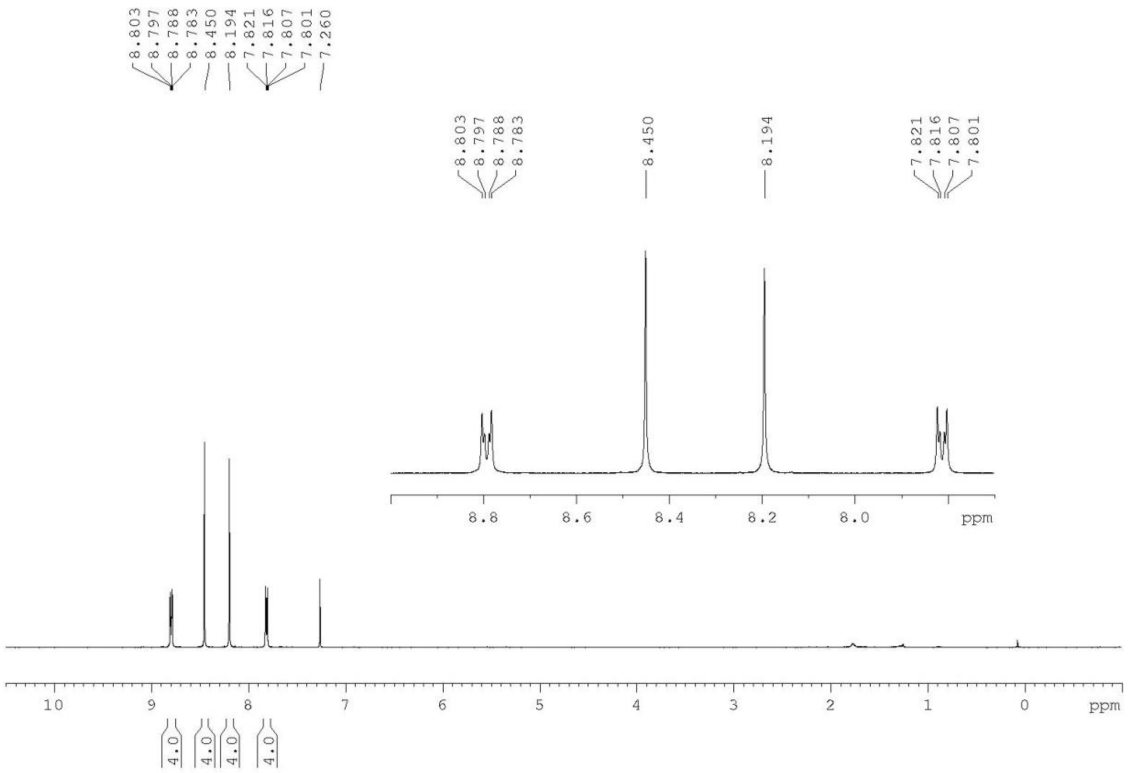

Figure S3: ^{1}H NMR (300 MHz, $CDCl_3$) spectrum of **2**.

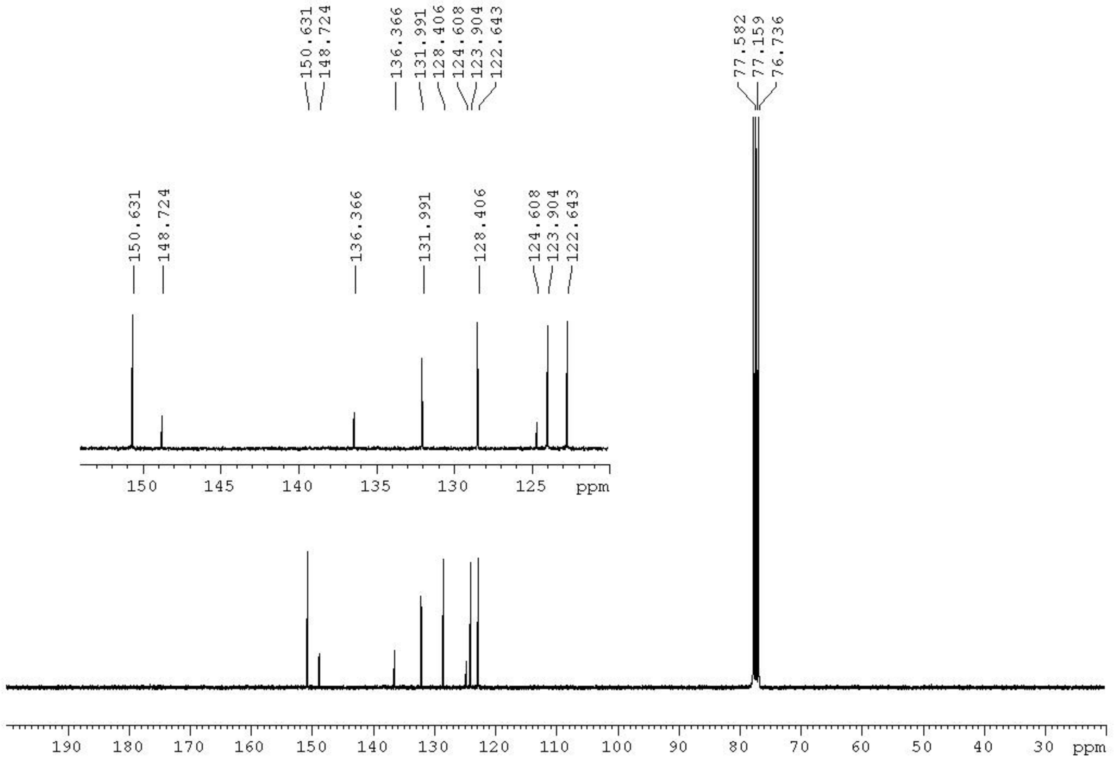

Figure S4: $^{13}C\{^{1}H\}$ NMR (75 MHz, $CDCl_3$) spectrum of **2**.

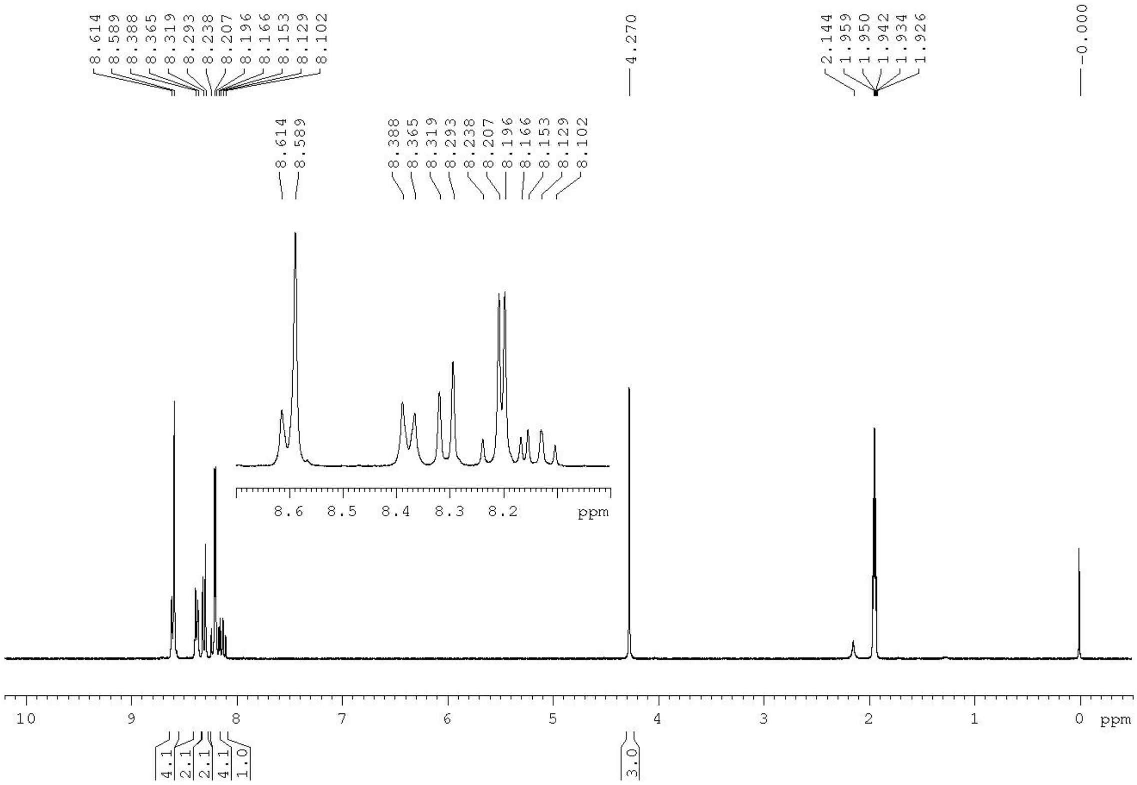

Figure S5: ^{1}H NMR (300 MHz, CD_3CN with 0.3 % TMS) spectrum of **1M**.

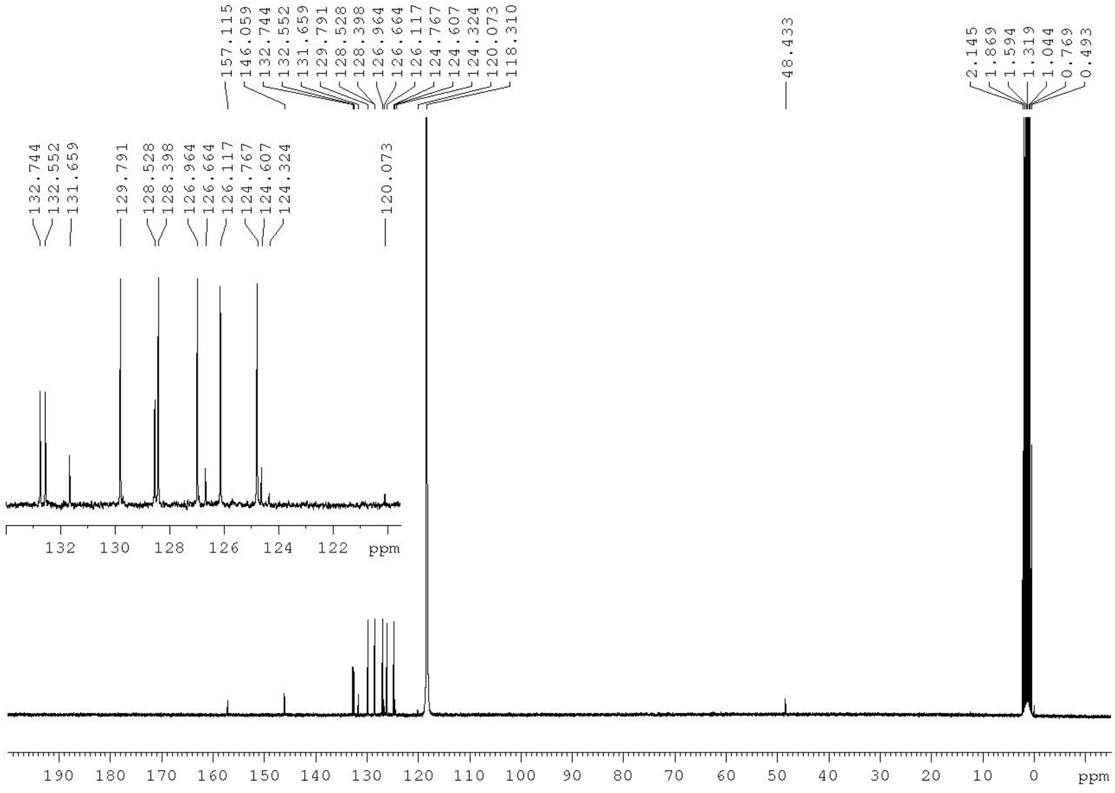

Figure S6: $^{13}C\{^{1}H\}$ NMR (75 MHz, CD_3CN with 0.3 % TMS) spectrum of **1M**.

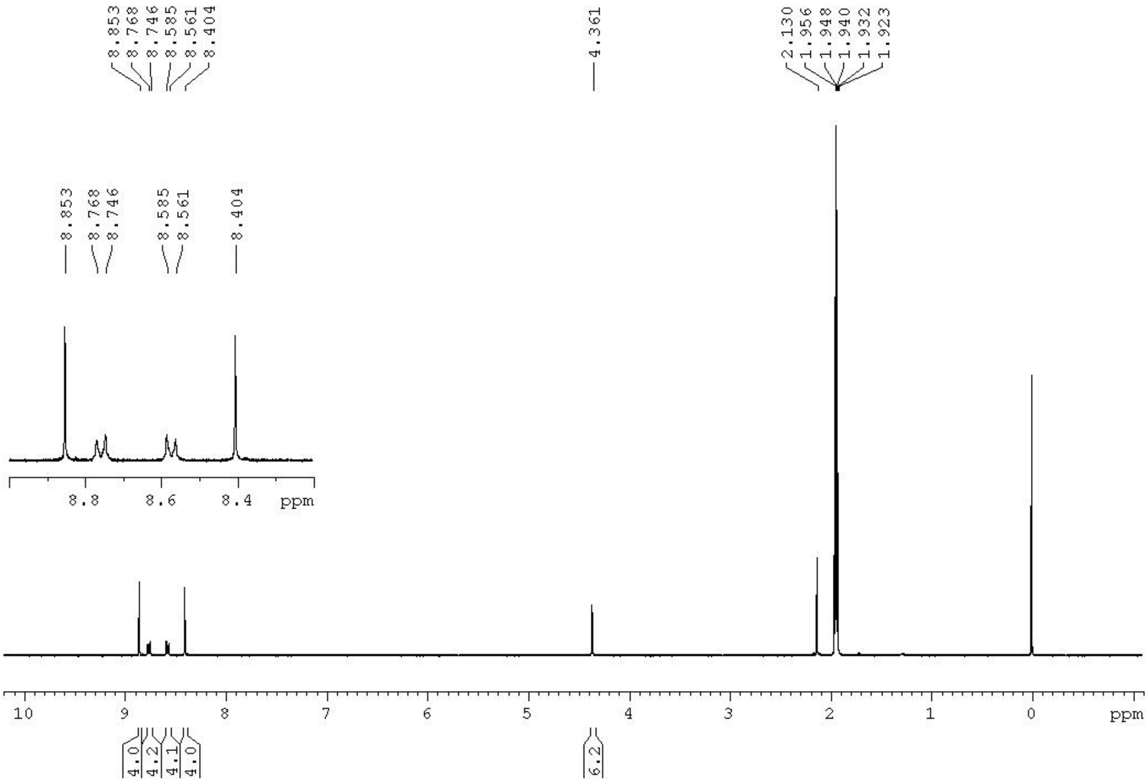

Figure S7: ^{1}H NMR (300 MHz, CD_3CN with 0.3 % TMS) spectrum of **2M**.

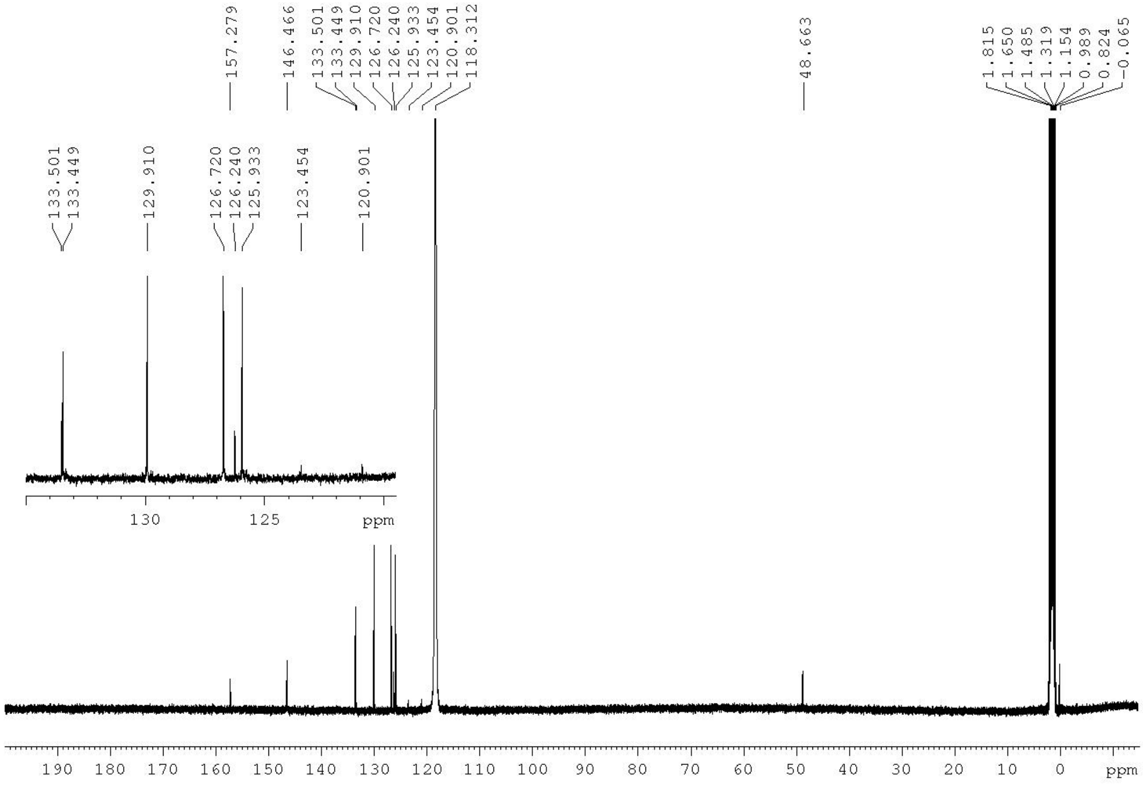

Figure S8: $^{13}C\{^1H\}$ NMR (75 MHz, CD_3CN with 0.3 % TMS) spectrum of **2M**.

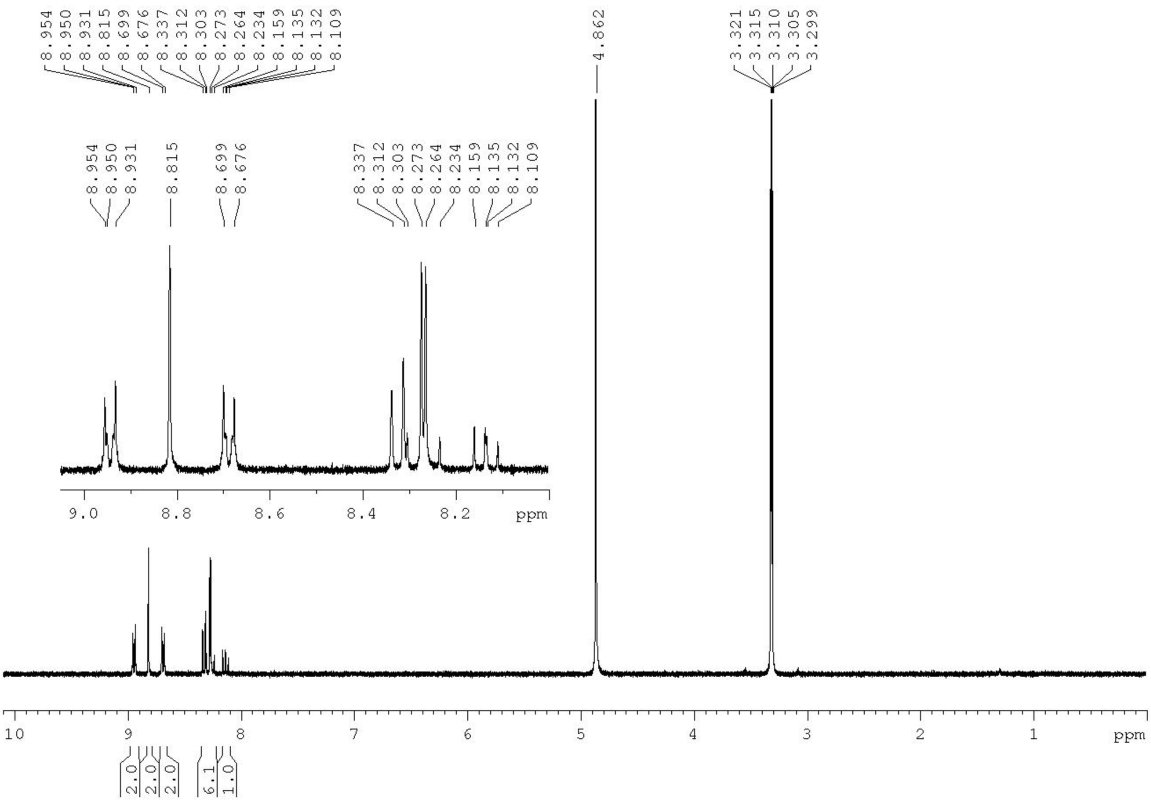

Figure S9: ^{1}H NMR (300 MHz, d_4-MeOD) spectrum of **1H**.

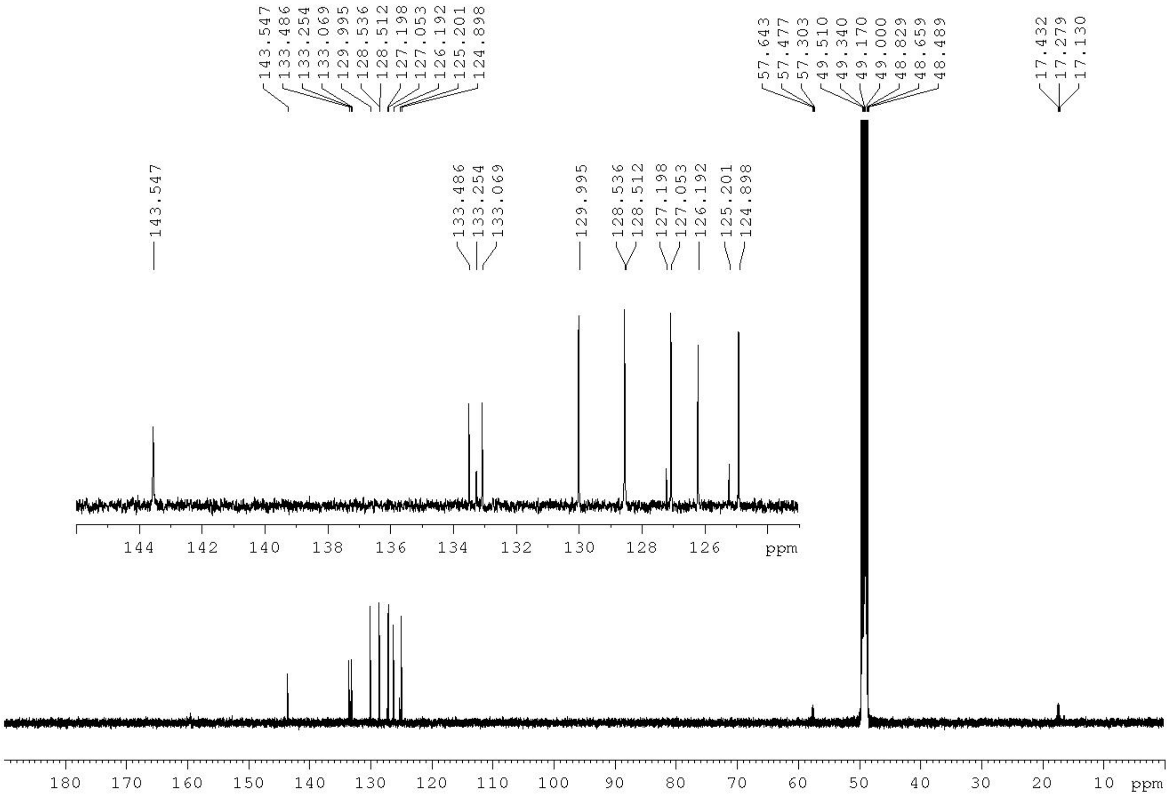

Figure S10: ^{13}C NMR (75 MHz, d_4-MeOD) spectrum of **1H**. The impurity peak at 17.3 and 57.5 may be due to ethanol.

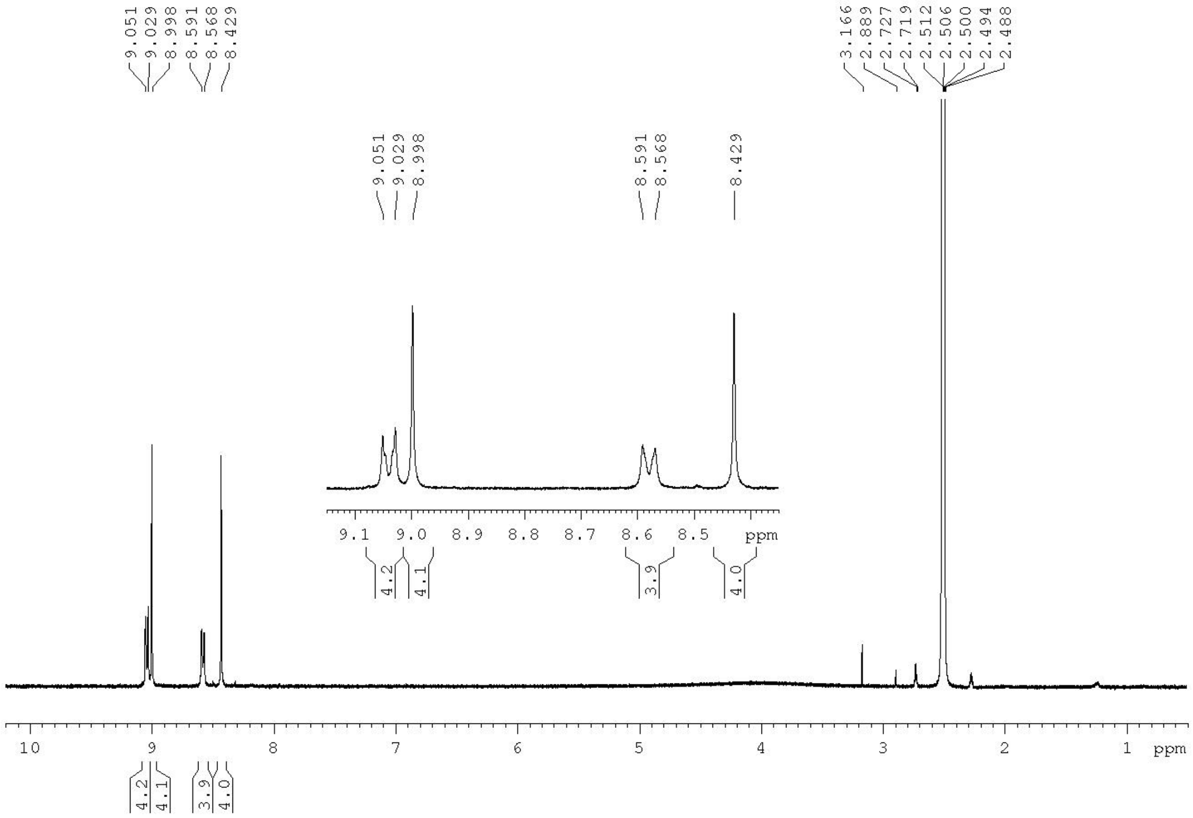

Figure S11: ^{1}H NMR (300 MHz, DMSO-d_6) spectrum of **2H**.

Table S1. Crystallographic data for **1M**, **2M**, **1H** and **2H**.

Data	**1M**	**2M**	**1H**	**2H**
CCDC number	2016794	2016796	2016793	2016795
Empirical formula	$C_{22}H_{16}N{\cdot}CF_3SO_3$	$C_{28}H_{22}N_2{\cdot}2(CF_3SO_3)$	$C_{21}H_{14}N_2O_3$	$C_{26}H_{18}N_4O_6$
Formula weight (g·mol^{-1})	443.43	684.61	342.34	482.44
Temperature (K)	100(2)	100(2)	100(2)	100(2)
Radiation, λ (Å)	MoK$_{\alpha}$ 0.71073	MoK$_{\alpha}$ 0.71073	Mo$_{K\alpha}$ 0.71073	Mo$_{K\alpha}$ 0.71073
Crystal colour, habit	yellow, needle	yellow, needle	yellow, needle	orange, needle
Crystal size (mm^3)	0.328 × 0.179 × 0.176	0.563 × 0.299 × 0.286	0.373 × 0.138 × 0.073	0.427 × 0.123 × × 0.0.054
Crystal system	Monoclinic	Monoclinic	Monoclinic	Monoclinic
Space group	$P2_1$	$C2/m$	$P2_1/c$	$P2_1/c$
Unit cell dimensions				
a (Å)	7.2780(11)	24.927(6)	6.735(2)	6.6546(14)
b (Å)	21.936(3)	8.910(2)	15.441(6)	8.9410(19)
c (Å)	12.115(2)	6.5960(17)	15.144(6)	17.965(4)
α (°)	90	90	90	90
β (°)	95.766(15)	96.199(7)	101.21(2)	97.259(5)
γ (°)	90	90	90	90
Volume (Å^3)	1924.4(5)	1456.4(6)	1544.7(10)	1060.4(4)
Z	4	2	4	2
Calculated density (Mg·m^{-3})	1.531	1.561	1.472	1.511
μ (mm^{-1})	0.224	0.270	0.100	0.110
F(000)	912	700	712	500
θ range (°)	1.689 – 26.372	2.429 – 26.363	2.638 – 26.372	3.601 – 26.360
Reflections collected	50468	12326	21948	25725
Independent reflections	7855	1592	3152	2159
Minimum/maximum transmission	0.7174/0.7457	0.5801/0.7457	0.6834/0.7244	0.6616/0.7456
Parameters / restraints	562 / 643	142 / 8	236 / 0	163 / 0
GooF on F^2	1.026	1.069	0.962	1.053
R1 [I>2σ(I)]	0.0278	0.0482	0.0494	0.0546
wR^2 (all data)	0.0751	0.1409	0.1266	0.1461
Maximum/minimum residual electron density (e·Å^{-3})	0.261 / –0.238	0.412 / –0.324	0.333 / –0.282	0.393 / –0.244

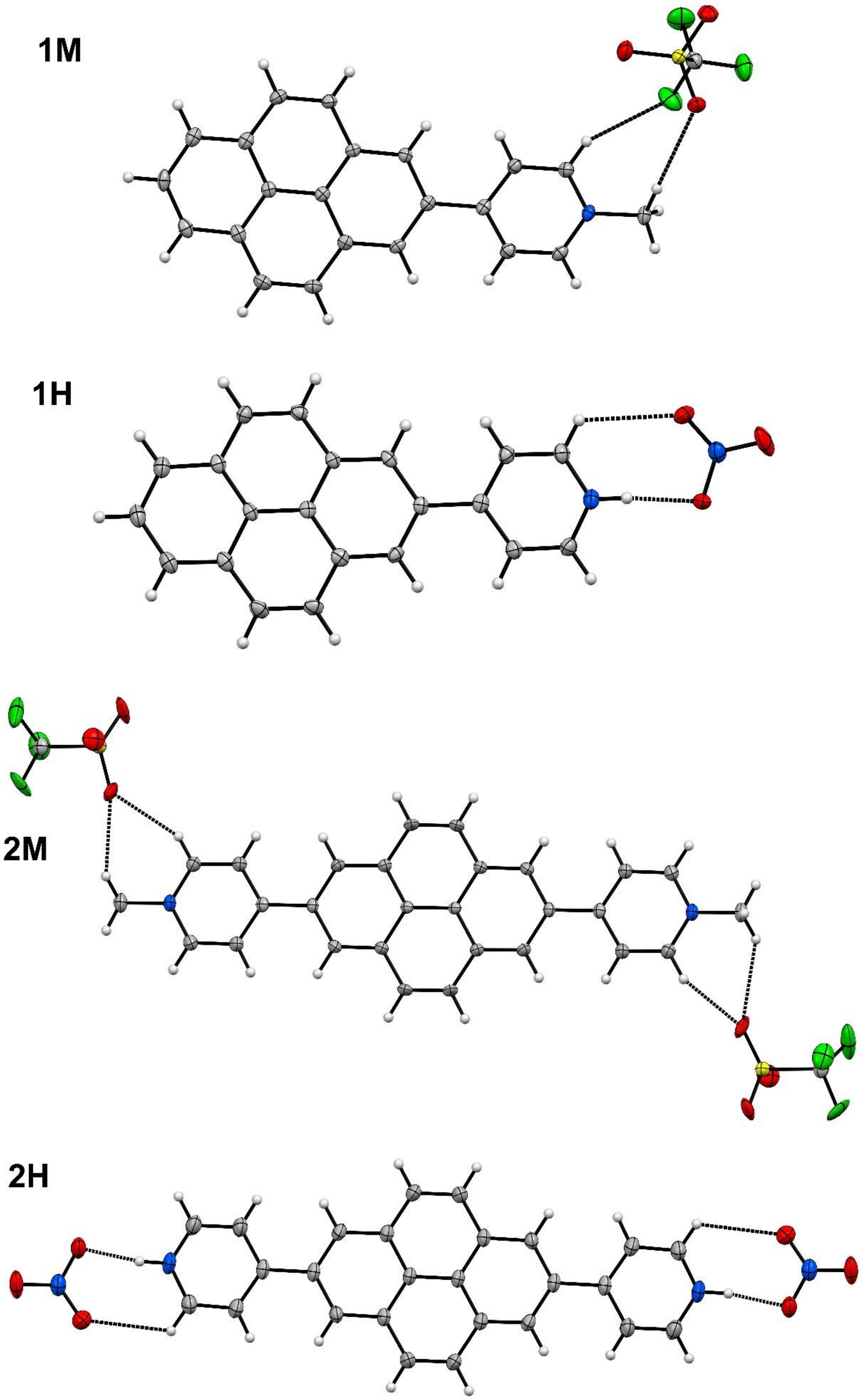

Figure S12: Molecular structures of **1M**, **1H**, **2M**, and **2H** in the solid state at 100 K. The atomic displacement ellipsoids are drawn with 50% probabilities. Colour codes: grey: carbon, blue: nitrogen, red: oxygen, green: fluorine, yellow: sulphur, and white: hydrogen.

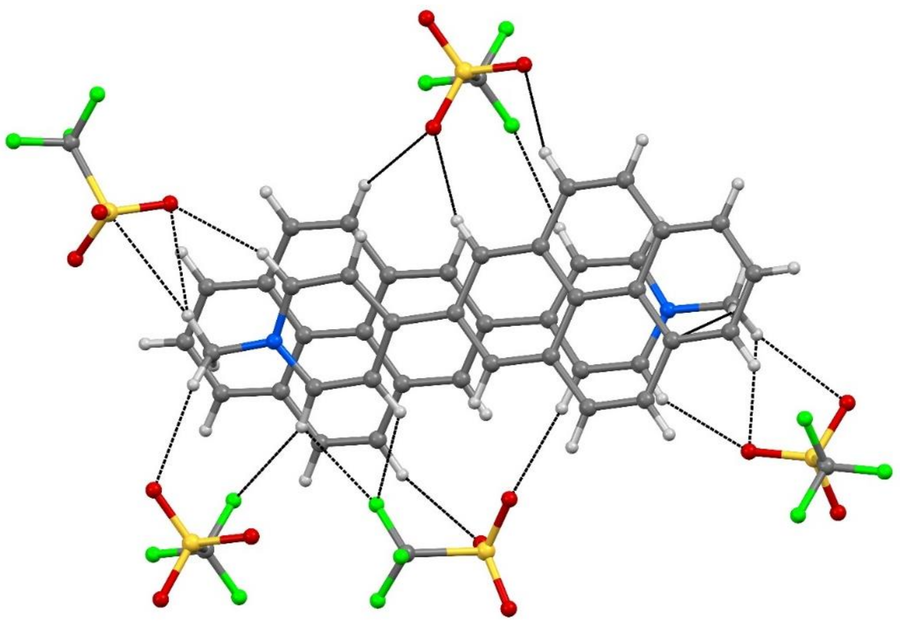

Figure S13: Projection of part of the crystal structure of **1M** along the *a*-axis. The stacking of the cations and various weak interactions with the surrounding triflate anions are shown. Colour codes: grey: carbon, blue: nitrogen, red: oxygen, green: fluorine, yellow: sulphur, and white: hydrogen.

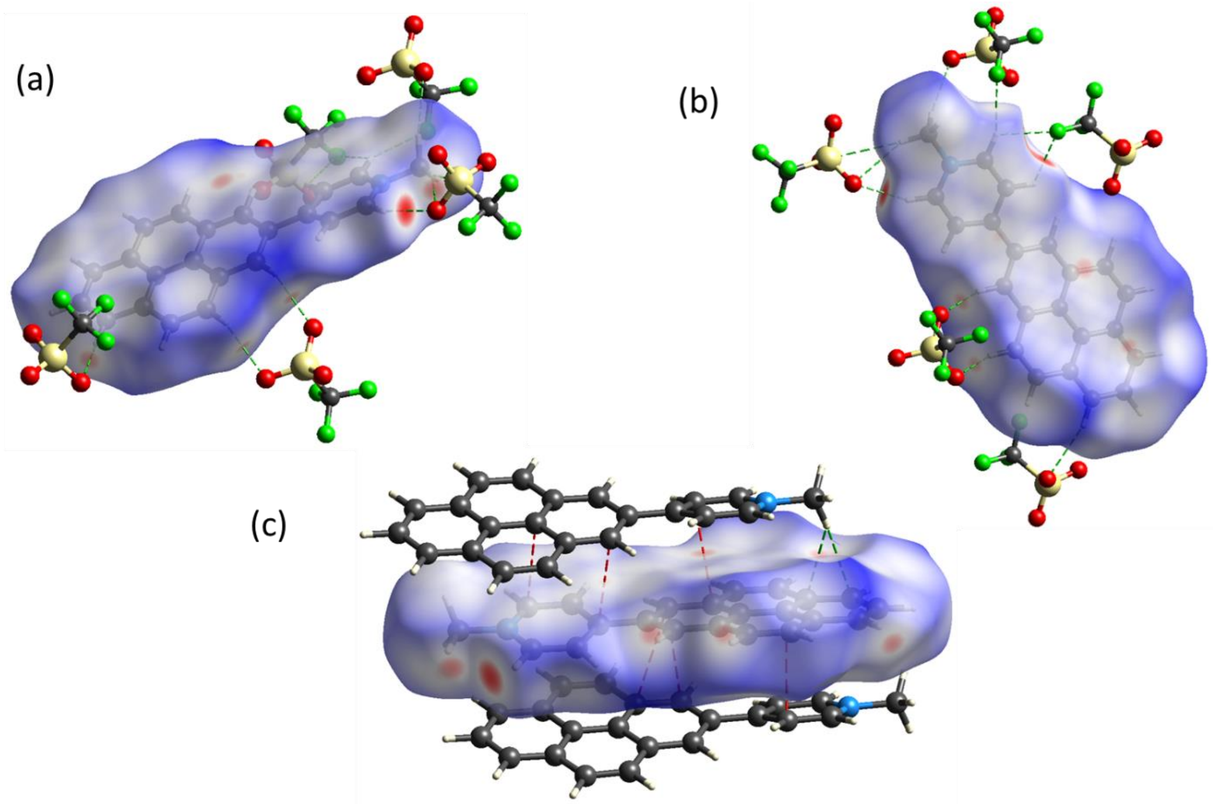

Figure S14: Hirshfeld surface for **1M**. Various interactions (C–F···C, C–H···O and C–H···S) between the cation and triflate anion are shown in (a) and (b), while (c) shows π-stacking and C–H···π interactions. Red areas indicate strong interactions, white areas moderate interactions and blue areas no significant interactions. Atom colour: black: carbon, red: oxygen, blue: nitrogen, green: fluorine, yellow: sulphur, and white: hydrogen.

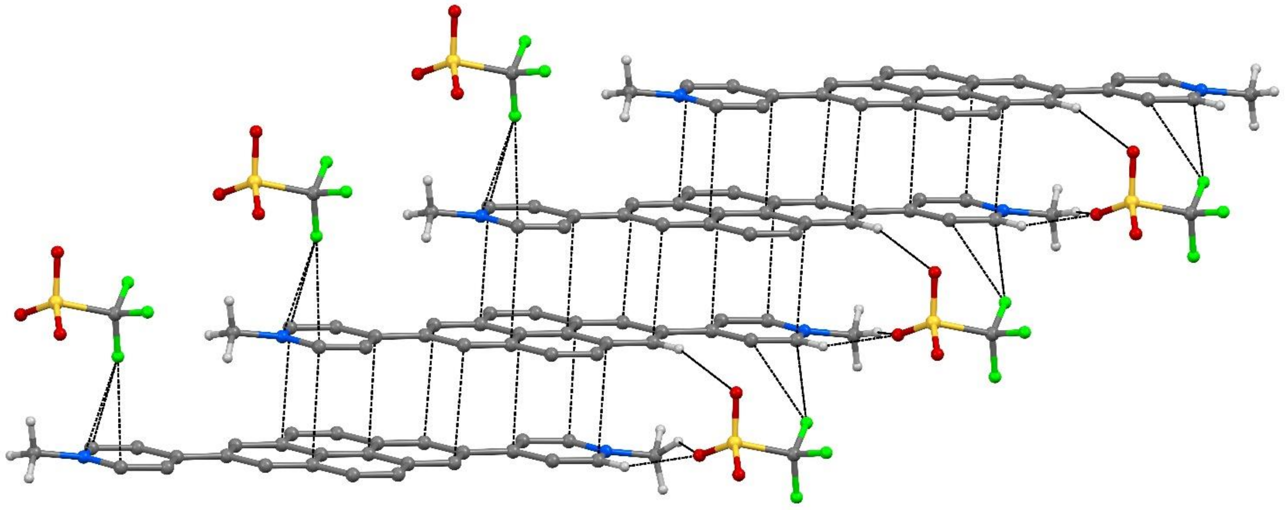

Figure S15. The infinite offset parallel stacking of the dication in **2M** and its various weak C–F···π and C–H···O interactions with the triflate anion are shown. Only selected hydrogen atoms are shown for clarity. Colour codes: grey: carbon, blue: nitrogen, red: oxygen, green: fluorine, yellow: sulphur, and white: hydrogen.

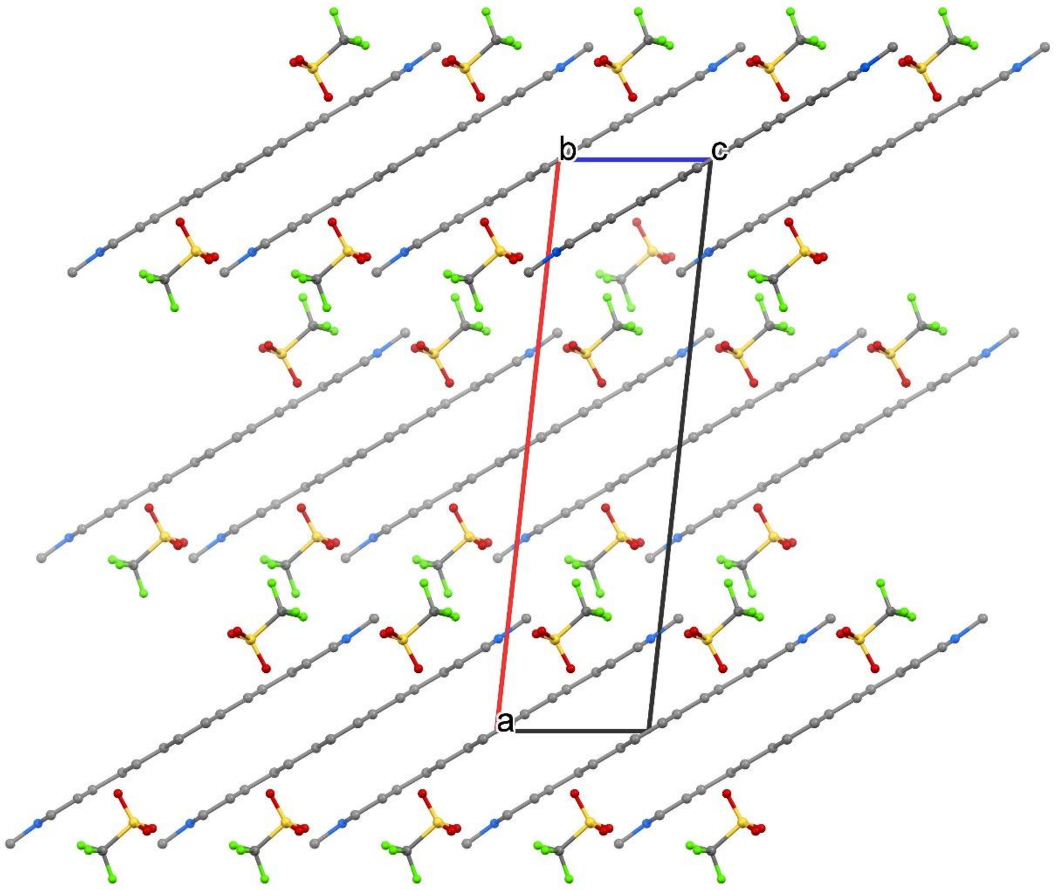

Figure S16: View of the crystal packing of **2M** along the *b*-axis. The side view shows the planarity of the dication and the offset parallel stacking. Hydrogen atoms are omitted for clarity. Colour codes: grey: carbon, blue: nitrogen, red: oxygen, green: fluorine, yellow: sulphur, and white: hydrogen.

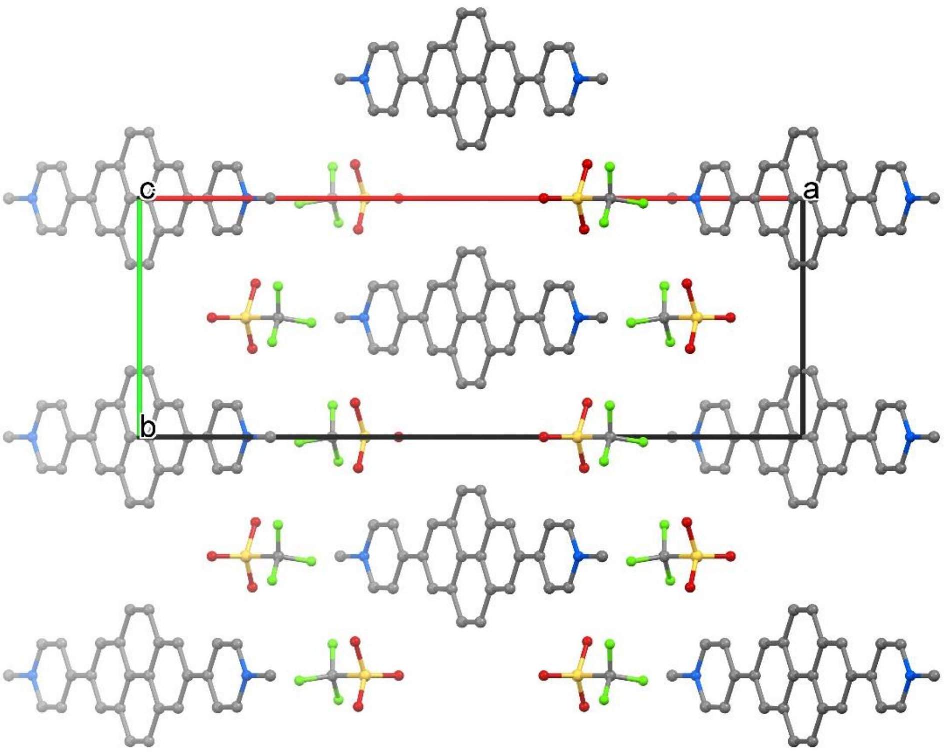

Figure S17: View of the crystal packing of **2M** along the *c*-axis, which is the stacking direction. Hydrogen atoms are omitted for clarity. Colour codes: grey: carbon, blue: nitrogen, red: oxygen, green: fluorine, yellow: sulphur, and white: hydrogen.

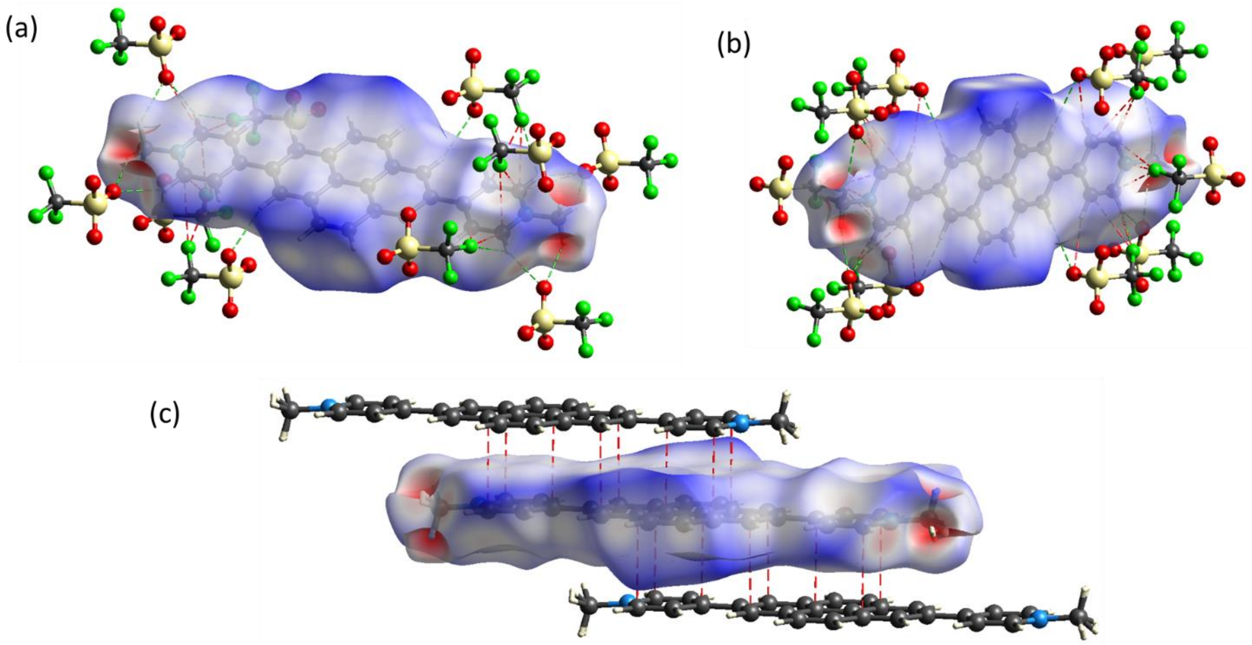

Figure S18: Hirshfeld surface for **2M**. Various interactions (C–F···C and C–H···O) between the dication and triflate anions are shown in (a) and (b), while (c) shows π-stacking interactions. Red areas indicate strong interactions, white areas moderate interactions and blue areas no significant interactions. Atom colour: black: carbon, red: oxygen, blue: nitrogen, green: fluorine, yellow: sulphur, and white: hydrogen.

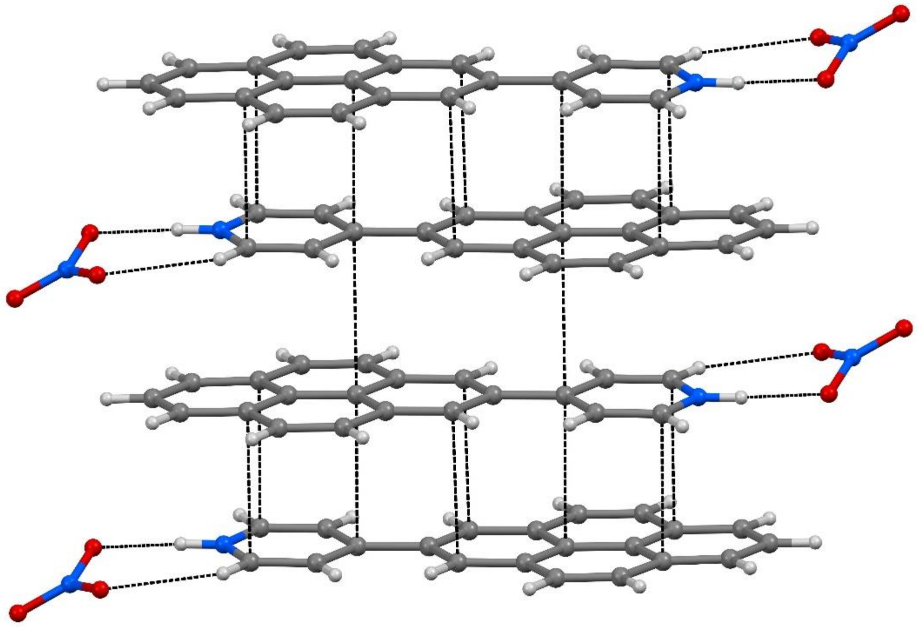

Figure S19: The crystal structure of **1H** shows the infinite parallel π-stacking of the cation in *head-to-tail* (anti) fashion. Various intermolecular interactions (C···C, C–H···O, and N–H···O) are plotted. Colour codes: grey: carbon, blue: nitrogen, red: oxygen, and white: hydrogen.

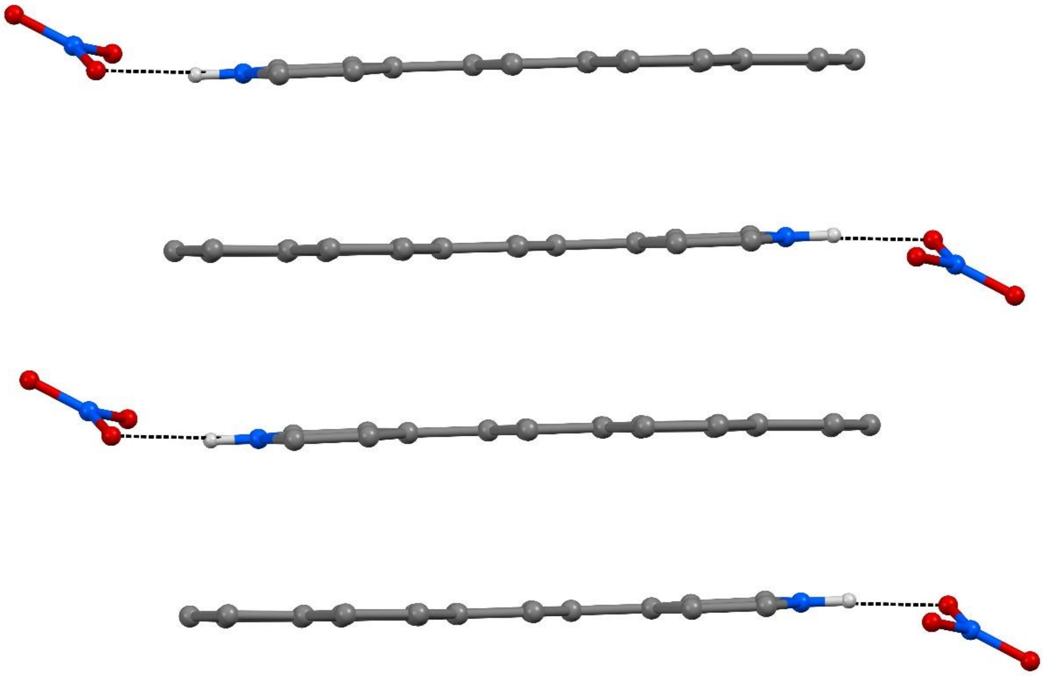

Figure S20: The side view of the cation in **1H** shows its near-planarity and the anti-parallel π-stacking arrangement. Only selected hydrogen atoms are shown for clarity. Colour codes: grey: carbon, blue: nitrogen, red: oxygen, and white: hydrogen.

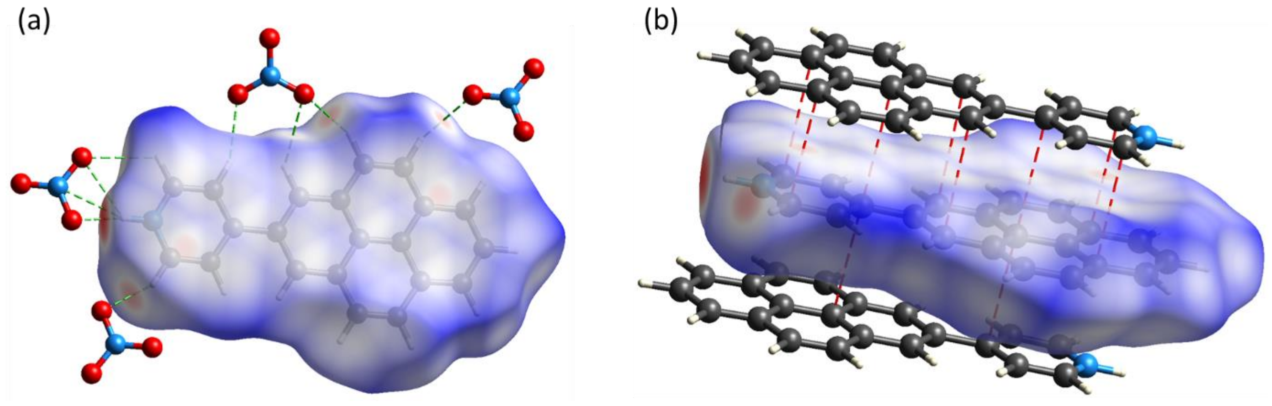

Figure S21: Hirshfeld surface for **1H**. The N–H···O and weak C–H···O hydrogen bonds are shown in (a), while the π-stacking interactions are shown in (b). Red areas indicate strong interactions, white areas moderate interactions and blue areas no significant interactions. Atom colour: black: carbon, red: oxygen, blue: nitrogen, and white: hydrogen.

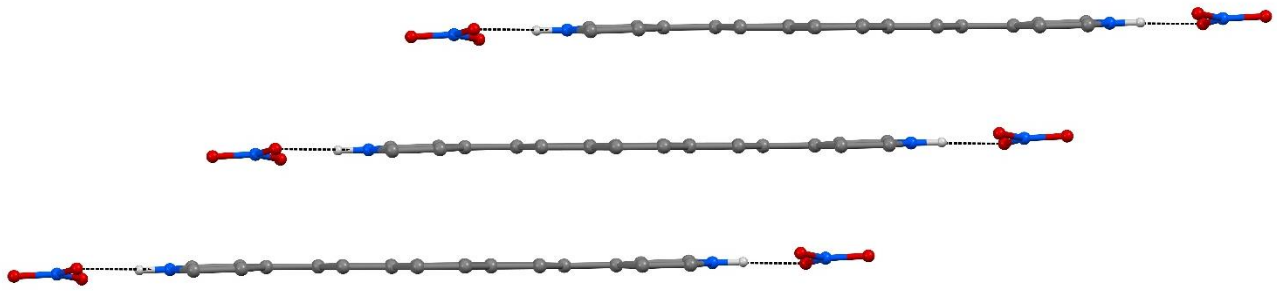

Figure S22: The side view of the cation in **2H** shows the planarity of the dication and the offset parallel π-stacking. Only selected hydrogen atoms are shown for clarity. Colour codes: grey: carbon, blue: nitrogen, red: oxygen, and white: hydrogen.

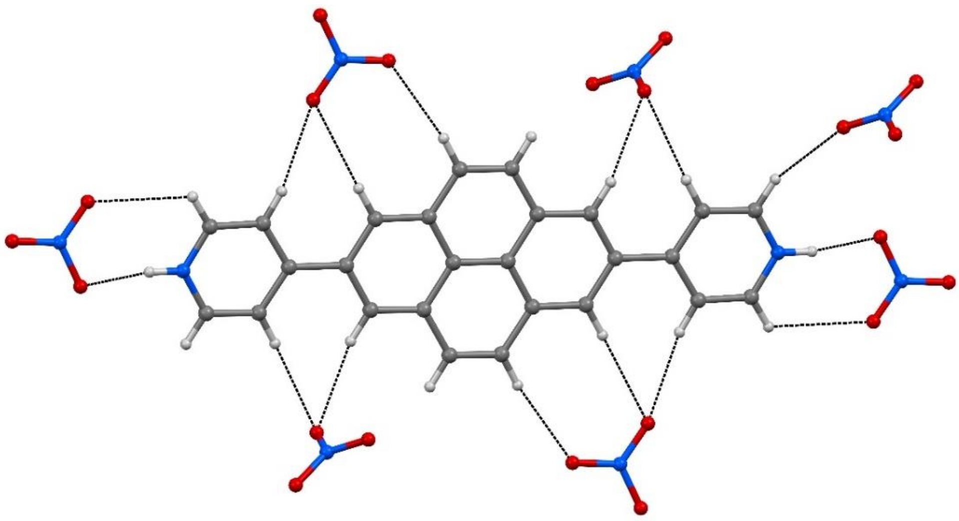

Figure S23: Weak intermolecular N–H···O and C–H···O interactions are shown between the dication in **2H** and the surrounding nitrate ions. Colour codes: grey: carbon, blue: nitrogen, red: oxygen, and white: hydrogen.

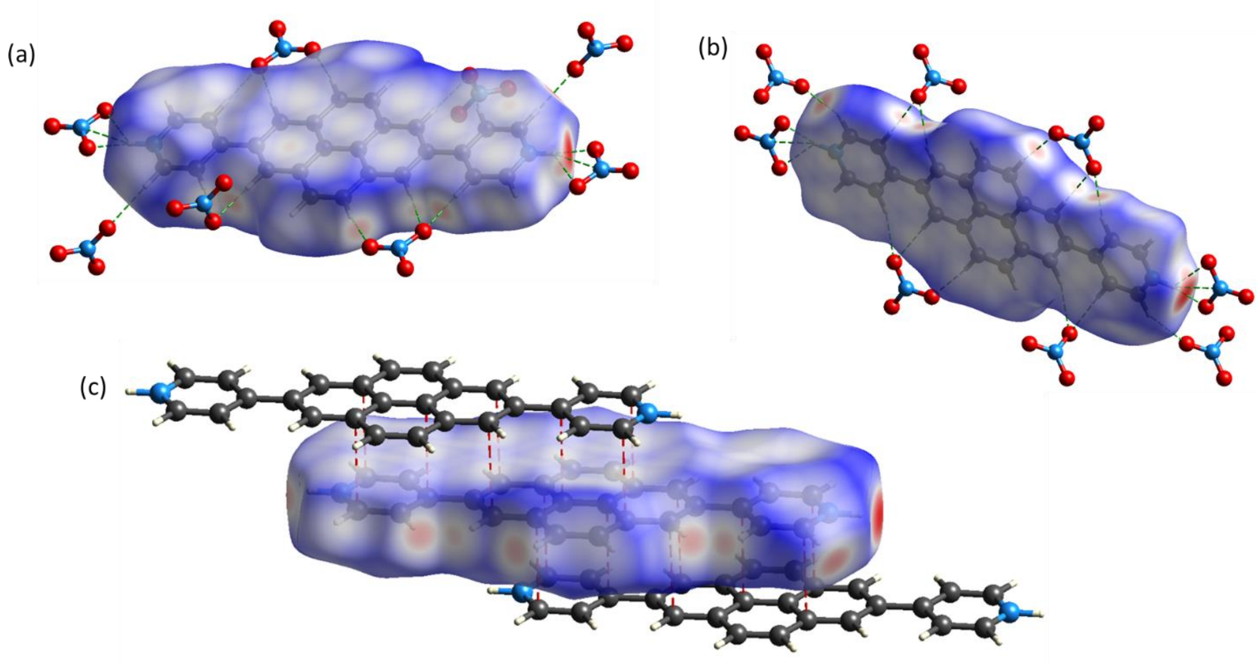

Figure S24: Hirshfeld surface for **2H**. The N-H···O and weak C-H···O hydrogen bonds are shown in (a) and (b), while the π-stacking interactions are shown in (c). Red areas indicate strong interactions, white areas moderate interactions and blue areas no significant interactions. Atom colour: black: carbon, red: oxygen, blue: nitrogen, and white: hydrogen.

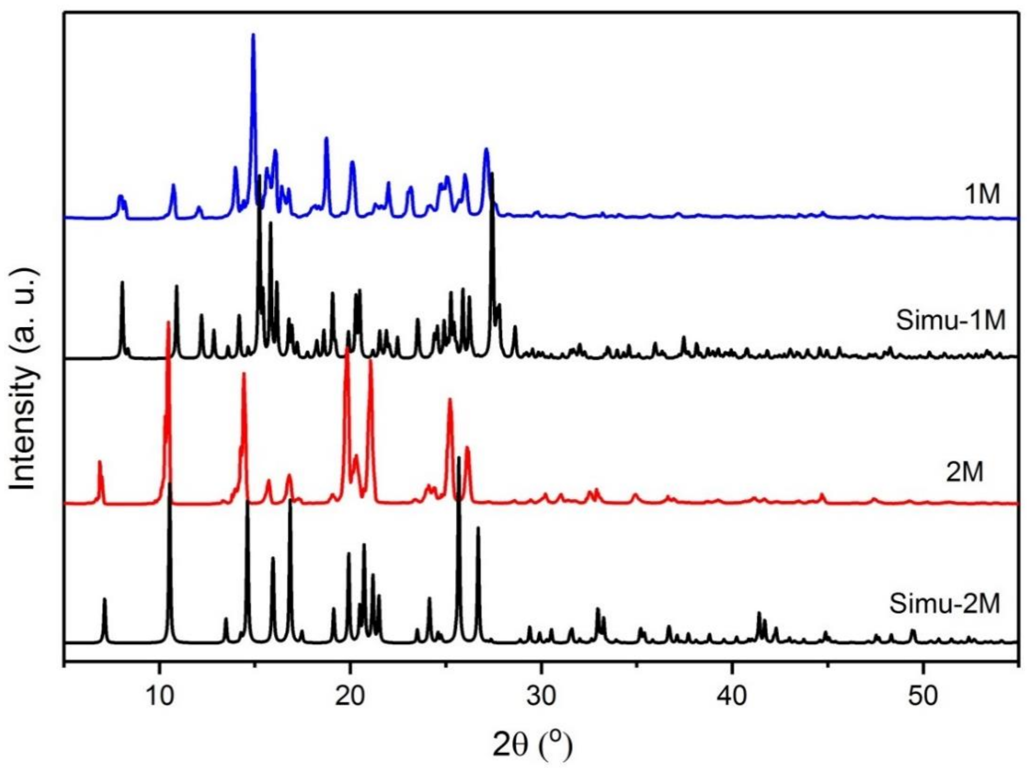

Figure S25: Simulated and experimentally observed powder X-ray diffraction patterns of **1M** and **2M**. Small systematic shifts between observed and calculated reflection positions are attributed to effects of sample preparation.

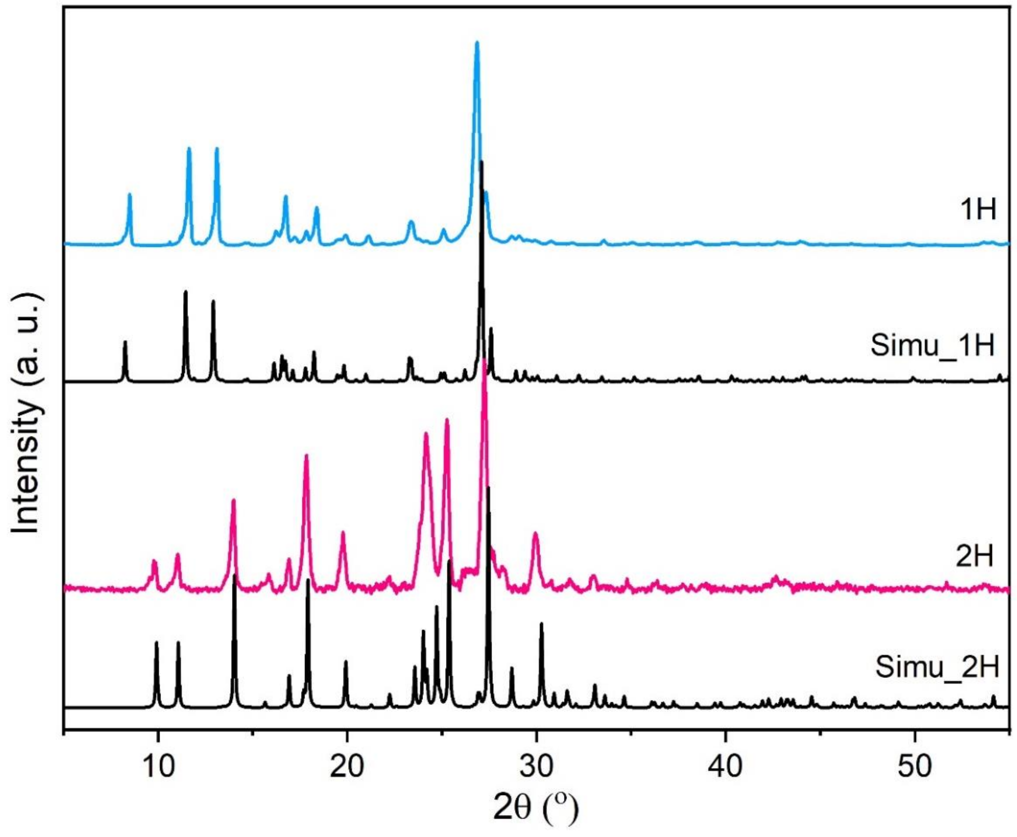

Figure S26: Simulated and experimentally observed powder X-ray diffraction patterns of **1H** and **2H**.

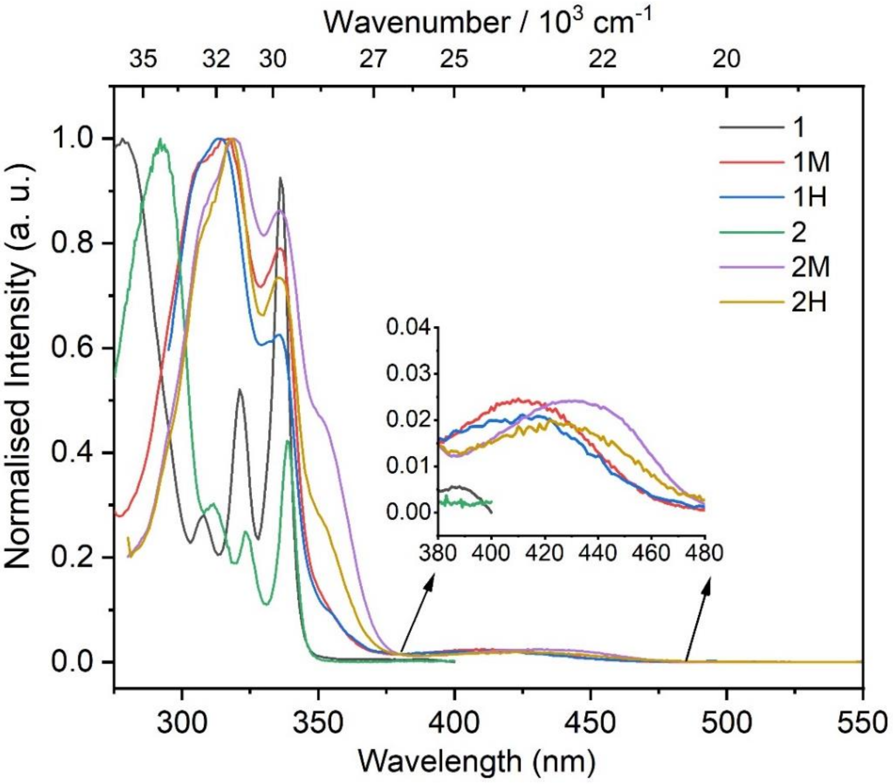

Figure S27: Normalised excitation spectra of **1**, **1M**, **1H**, **2**, **2M**, and **2H**.

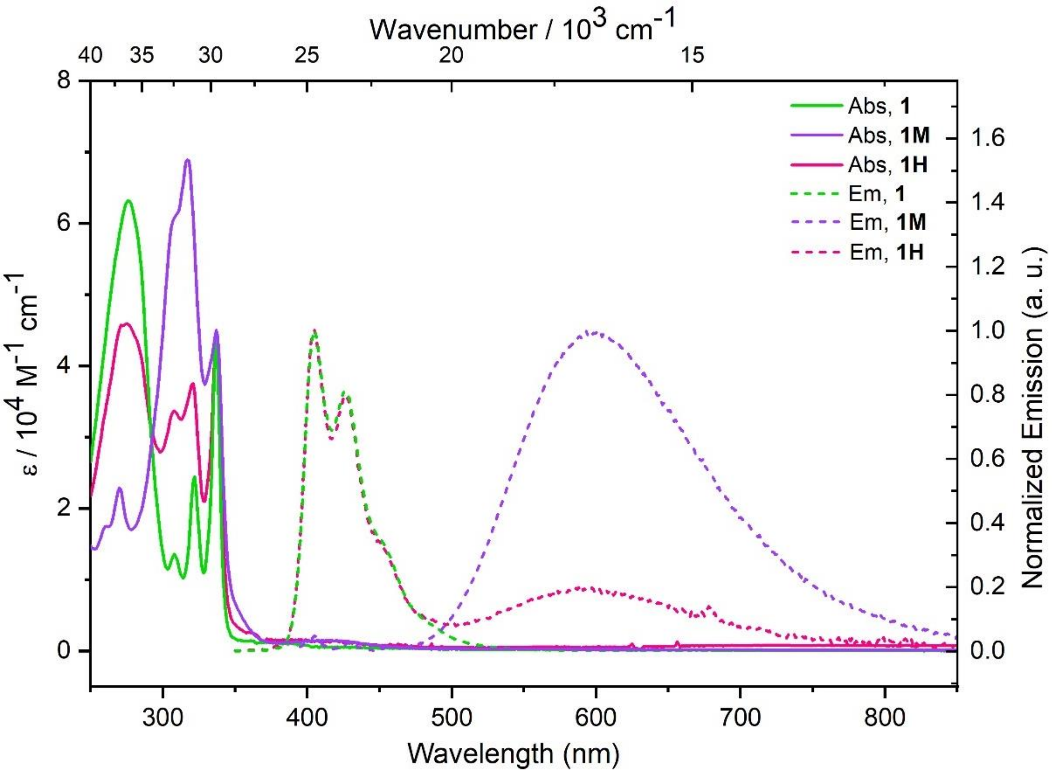

Figure S28: The absorption (solid lines) and emission (dashed lines) of **1H** (in MeCN) are compared with that of **1** and **1M**. This shows that the salt remains in equilibrium between protonated and neutral forms in solution.

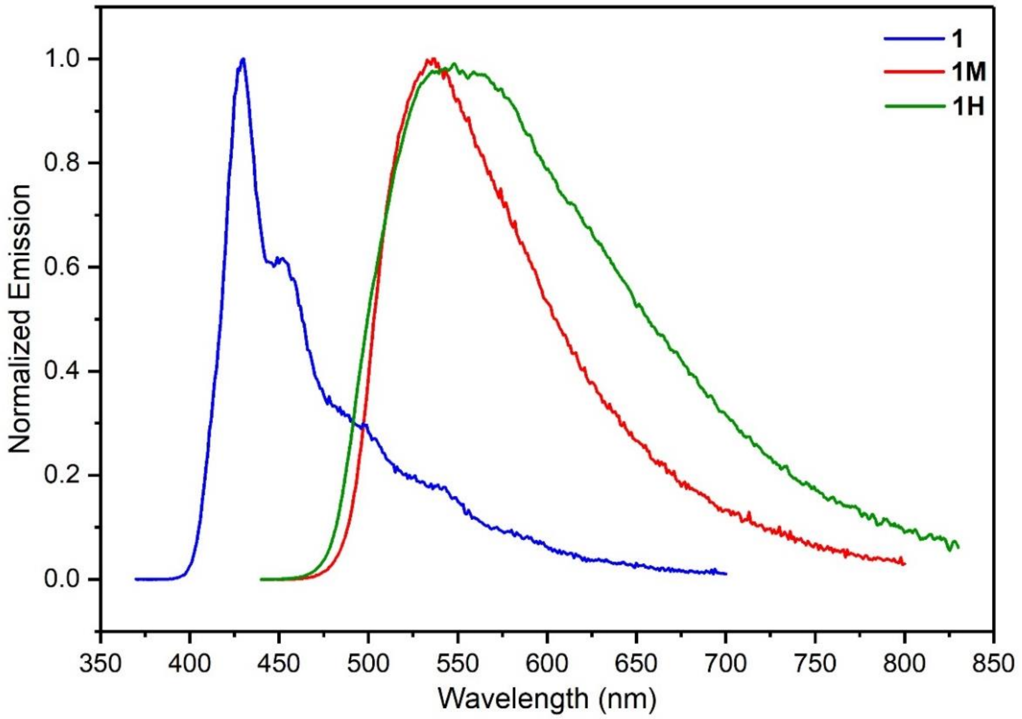

Figure S29: Solid state emission of **1**, **1M**, and **1H**. The emission of both **1M** and **1H** are bathochromically shifted compared to that of **1**.

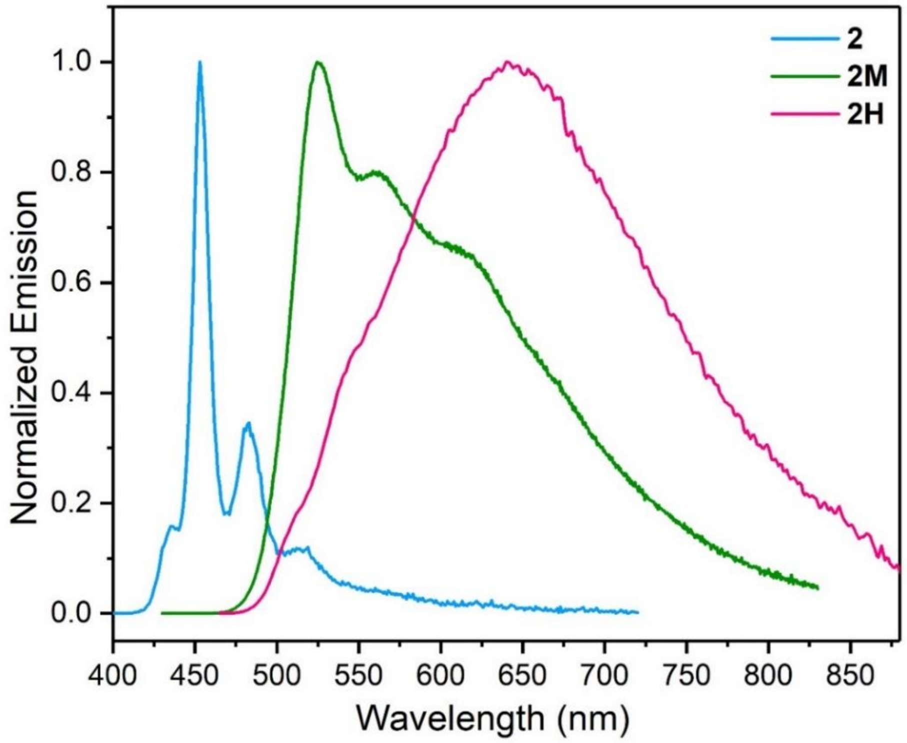

Figure S30: Solid state emission of **2**, **2M**, and **2H**. The emission of both **2M** and **2H** are bathochromically shifted compared to that of **2**.

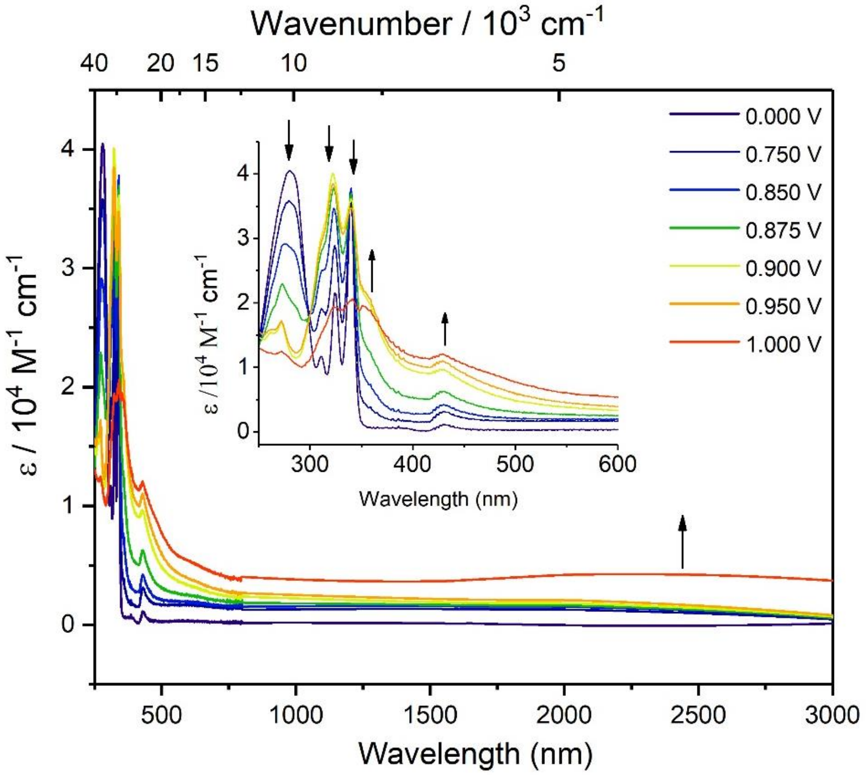

Figure S31: Spectroelectrochemical measurements of the stepwise oxidation process of **1** in DCM/0.1M [n-Bu_4N][PF_6]. The changes of transition intensities are shown with upward and downward arrows. A broad brand in the NIR region can be observed.

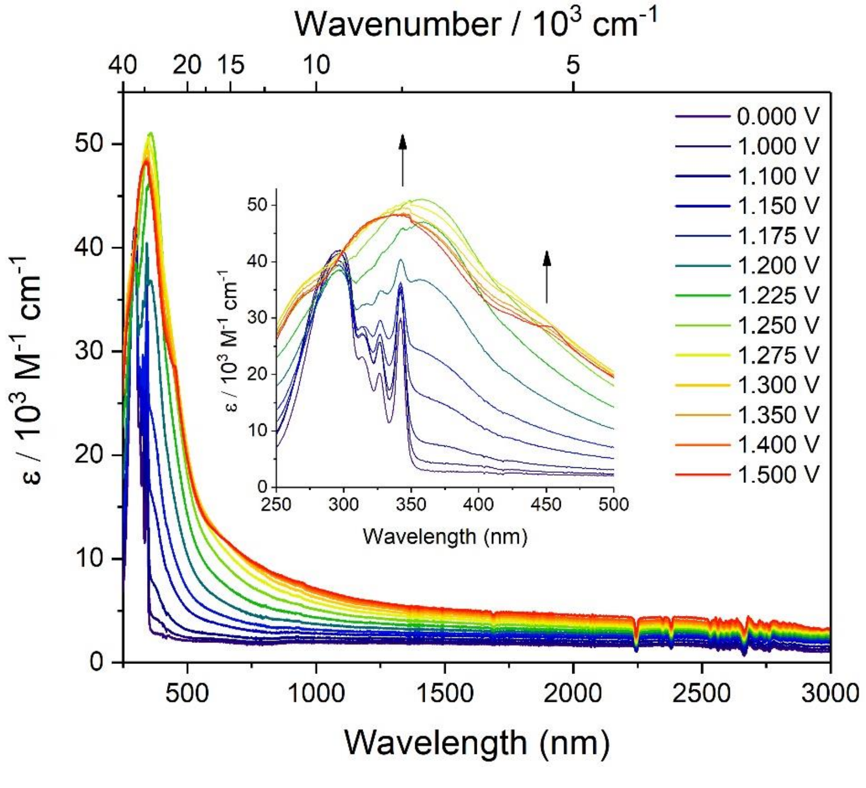

Figure S32: Spectroelectrochemical measurements of the stepwise oxidation process of **2** in DCM/0.1M [n-Bu_4N][PF_6]. A very broad band with tail extended into the visible region can be observed.

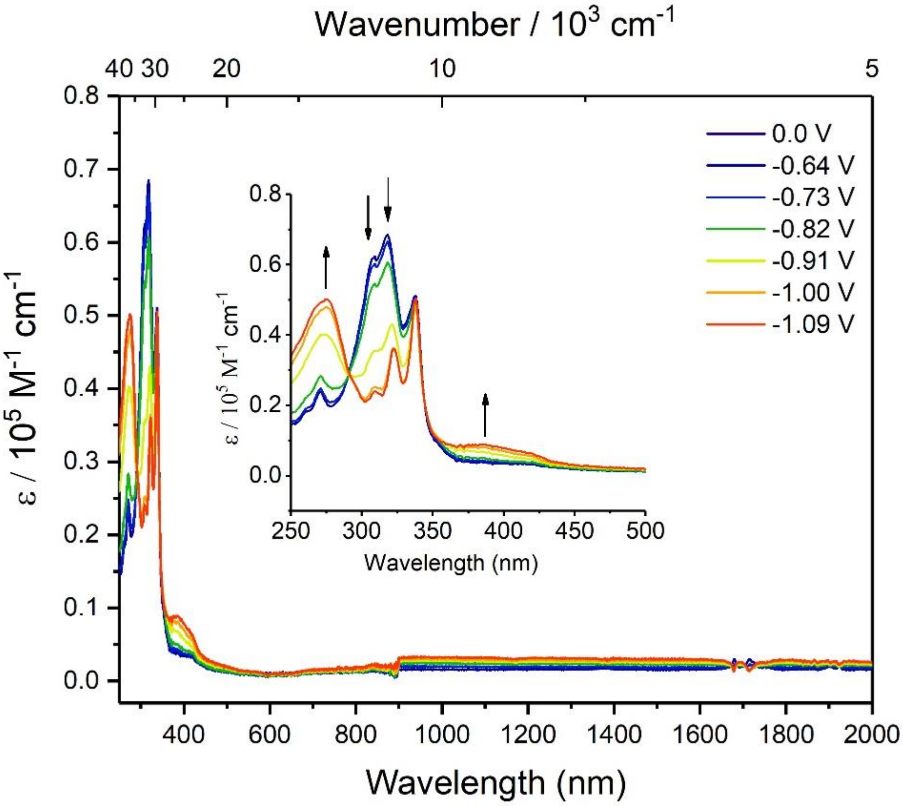

Figure S33: Spectroelectrochemical measurements of the stepwise reduction process of **1M** in MeCN/0.1M $[n\text{-}Bu_4N][PF_6]$. The changes of transition intensities are shown with upward and downward arrows. No absorption in the NIR region was observed.

Table S2. Comparison of selected experimentally determined and theoretically optimized structural parameters (PBE0-GD3BJ/LANL2DZP in THF) for compounds **1**, **1H**, and **1M**.

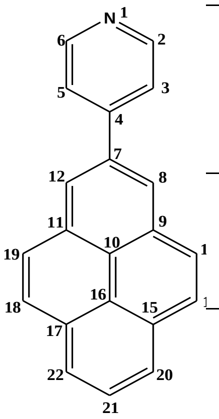

		**1**[1]		**1H**		**1M**		
		Exp.	Opt.	Exp.	Opt.	Exp.[a]	Exp.[a]	Opt.
Bond lengths (Å)	C3-C4	1.397(2)	1.40	1.395(3)	1.41	1.398(3)	1.403(3)	1.41
	C4-C5	1.396(2)	1.40	1.395(3)	1.41	1.399(3)	1.401(3)	1.41
	C4-C7	1.489(2)	1.48	1.482(3)	1.47	1.486(3)	1.485(3)	1.47
	C7-C8	1.398(2)	1.40	1.398(3)	1.40	1.401(3)	1.402(3)	1.40
	C7-C12	1.398(2)	1.40	1.401(3)	1.40	1.404(3)	1.396(3)	1.40
Bond angles (°)	C3-C4-C7	120.89(12)	121.68	121.9(2)	121.36	122.9(2)	121.2(2)	121.70
	C4-C7-C8	121.26(12)	120.37	120.0(2)	120.13	121.0(2)	119.8(2)	120.17
	C5-C4-C7	122.12(12)	121.68	121.6(2)	121.36	120.7(2)	121.7(2)	121.80
	C4-C7-C12	118.95(12)	120.37	121.3(2)	120.13	119.6(2)	120.7(2)	120.17
Torsion angles (°)	C3-C4-C7-C8	35.48	33.73	1.40	29.58	-17.65	-21.06	28.14
	C5-C4-C7-C12	35.23	33.73	1.37	29.58	-17.74	-21.07	28.32

[a] There are two symmetry-independent molecules in the asymmetric unit.

Table S3. Comparison of selected experimentally determined and theoretically optimized structural parameters (PBE0-GD3BJ/LANL2DZP in THF) for compounds **2**, **2H**, **a**nd **2M**.

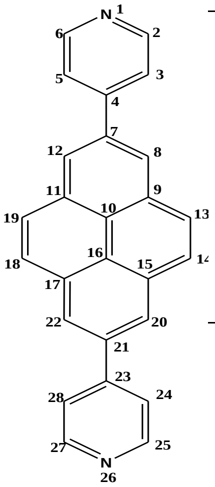

		**2**[1]			**2H**		**2M**	
		Exp.$^{\alpha}$	Exp.$^{\beta}$	Opt.	Exp.	Opt.	Exp.	Opt.
Bond lengths (Å)	C3-C4	1.394(2)	1.391(2)	1.40	1.399(4)	1.41	1.396(2)	1.41
	C4-C5	1.395(2)	1.389(2)	1.40	1.404(3)	1.41	1.396(2)	1.41
	C4-C7	1.491(2)	1.482(2)	1.48	1.491(3)	147	1.489(3)	1.47
	C7-C8	1.401(2)	1.397(2)	1.40	1.401(3)	1.40	1.399(2)	1.40
	C7-C12	1.397(2)	1.396(2)	1.40	1.397(3)	1.40	1.399(2)	1.40
	C20-C21	1.397(2)	1.396(2)	1.40	1.397(3)	1.40	1.399(2)	1.40
	C21-C22	1.401(2)	1.397(2)	1.40	1.401(3)	1.40	1.399(2)	1.40
	C21-C23	1.491(2)	1.482(2)	1.48	1.491(3)	1.47	1.489(3)	1.47
	C23-C24	1.395(2)	1.389(2)	1.40	1.404(3)	1.41	1.396(2)	1.41
	C23-C28	1.394(2)	1.391(2)	1.40	1.399(4)	1.41	1.396(2)	1.41
Bond angles (°)	C3-C4-C7	121.56(13)	121.90(15)	121.63	122.0(2)	121.22	121.59(11)	122.02
	C4-C7-C8	120.39(13)	120.63(15)	120.31	120.6(2)	120.07	120.55(11)	120.49
	C5-C4-C7	122.48(13)	121.52(15)	121.63	121.1(2)	121.22	121.59(11)	122.02
	C4-C7-C12	121.03(13)	120.40(14)	120.31	120.0(2)	120.07	120.55(11)	120.49
	C20-C21-C23	121.03(13)	120.40(14)	120.31	120.0(2)	120.07	120.55(11)	120.49
	C21-C23-C24	122.48(13)	121.52(15)	121.63	121.1(2)	121.22	121.59(11)	122.02
	C22-C21-C23	120.39(13)	120.63(15)	120.31	120.6(2)	120.07	120.55(11)	120.49
	C21-C23-C28	121.56(13)	121.90(15)	121.63	122.0(2)	121.22	121.59(11)	122.02
Torsion angles (°)	C3-C4-C7-C8	-12.49	-37.76	32.67	-0.96	28.83	0.53	0.00
	C5-C4-C7-C12	-12.32	-37.06	32.67	-1.25	28.83	-0.53	0.00
	C20-C21-C23-C24	12.32	37.06	32.67	1.25	-28.83	0.53	0.00
	C22-C21-C23-C28	12.49	37.76	-32.67	0.96	-28.83	-0.53	0.00

There are two polymorphs of **2** – α and β.

Table S4. Coordinates of the optimized geometries of compounds **1**, **2**, **1M**, **2M**, **1H**, and **2H**.

**1**	**2**
1 N1 -6.0672 -0.0000 0.0000 N	1 N1 1.9444 -4.9908 5.7435 N
2 C2 -1.0389 -1.2074 -0.0852 C	2 C2 1.4371 -5.4180 4.5779 C
3 H3 -1.5793 -2.1496 -0.1783 H	3 H3 1.3069 -6.4860 4.4792 H
4 C4 -1.7482 0.0000 -0.0000 C	4 C4 1.0758 -4.5744 3.5262 C
5 C5 -1.0389 1.2074 0.0852 C	5 H5 0.6521 -4.9952 2.6288 H
6 H6 -1.5792 2.1496 0.1783 H	6 C6 1.2419 -3.1876 3.6684 C
7 C7 0.3635 1.2285 0.0911 C	7 C7 1.7725 -2.7366 4.8874 C
8 C8 1.1056 2.4578 0.1914 C	8 H8 1.9517 -1.6882 5.0625 H
9 H9 0.5525 3.3947 0.2649 H	9 C9 2.1018 -3.6656 5.8756 C
10 C10 4.6228 1.2107 0.0951 C	10 H10 2.5163 -3.3275 6.8144 H
11 H11 5.1708 2.1503 0.1690 H	11 C11 0.8757 -2.2476 2.5866 C
12 C12 3.2174 1.2312 0.0965 C	12 C12 0.3932 -0.9665 2.8947 C
13 C13 2.4712 2.4580 0.1931 C	13 H13 0.2644 -0.6738 3.9255 H
14 H14 3.0225 3.3959 0.2684 H	14 C14 0.0376 -0.0623 1.8836 C
15 C15 5.3153 -0.0000 0.0000 C	15 C15 0.1760 -0.4519 0.5200 C
16 H16 6.4051 -0.0000 0.0000 H	16 C16 0.6687 -1.7507 0.2028 C
17 C17 4.6228 -1.2107 -0.0951 C	17 C17 1.0074 -2.6286 1.2425 C
18 H18 5.1708 -2.1503 -0.1690 H	18 H18 1.4016 -3.6025 0.9957 H
19 C19 3.2174 -1.2312 -0.0965 C	19 C19 0.8087 -2.1194 -1.1819 C
20 C20 2.4712 -2.4580 -0.1931 C	20 H20 1.1872 -3.1024 -1.4172 H
21 H21 3.0225 -3.3959 -0.2684 H	21 C21 0.4706 -1.2517 -2.1805 C
22 C22 1.1056 -2.4578 -0.1914 C	22 H22 0.5786 -1.5403 -3.2149 H
23 H23 0.5525 -3.3947 -0.2649 H	23 N23 -1.9444 4.9908 -5.7435 N
24 C24 0.3635 -1.2285 -0.0912 C	24 C24 -1.4371 5.4180 -4.5779 C
25 C25 1.0806 0.0000 -0.0000 C	25 H25 -1.3069 6.4860 -4.4792 H
26 C26 2.5045 0.0000 -0.0000 C	26 C26 -1.0758 4.5744 -3.5262 C
27 C27 -3.2276 0.0000 0.0000 C	27 H27 -0.6521 4.9952 -2.6288 H
28 C28 -3.9651 -1.0378 0.5919 C	28 C28 -1.2419 3.1876 -3.6684 C
29 H29 -3.4673 -1.8666 1.0932 H	29 C29 -1.7725 2.7366 -4.8874 C
30 C30 -5.3600 -0.9892 0.5649 C	30 H30 -1.9517 1.6882 -5.0625 H
31 H31 -5.9390 -1.7900 1.0293 H	31 C31 -2.1018 3.6656 -5.8756 C
32 C32 -5.3600 0.9891 -0.5649 C	32 H32 -2.5163 3.3275 -6.8144 H
33 H33 -5.9390 1.7900 -1.0293 H	33 C33 -0.8757 2.2476 -2.5866 C
34 C34 -3.9651 1.0378 -0.5919 C	34 C34 -0.3932 0.9665 -2.8947 C
35 H35 -3.4673 1.8666 -1.0932 H	35 H35 -0.2644 0.6738 -3.9255 H
	36 C36 -0.0376 0.0623 -1.8836 C
	37 C37 -0.1760 0.4519 -0.5200 C
	38 C38 -0.6687 1.7507 -0.2028 C
	39 C39 -1.0074 2.6286 -1.2425 C
	40 H40 -1.4016 3.6025 -0.9957 H
	41 C41 -0.8087 2.1194 1.1819 C
	42 H42 -1.1872 3.1024 1.4172 H
	43 C43 -0.4706 1.2517 2.1805 C
	44 H44 -0.5786 1.5403 3.2149 H
**1M**	**2M**
1 N1 5.5741 0.0063 -0.0079 N	1 N1 -4.7181 0.0002 6.1969 N
2 C2 0.6017 -1.2120 0.0633 C	2 C2 -3.9760 -1.0926 5.9172 C
3 H3 1.1370 -2.1568 0.1519 H	3 H3 -4.0769 -1.9279 6.5868 H
4 C4 1.3072 0.0007 -0.0099 C	4 C4 -3.1252 -1.1171 4.8288 C
5 C5 0.6004 1.2128 -0.0814 C	5 H5 -2.5355 -2.0017 4.6666 H
6 H6 1.1345 2.1580 -0.1725 H	6 C6 -3.0179 0.0038 3.9849 C
7 C7 -0.8002 1.2314 -0.0879 C	7 C7 -2.1250 0.0042 2.8153 C
8 C8 -1.5414 2.4618 -0.1779 C	8 C8 -1.8513 -1.1972 2.1417 C
9 H9 -0.9896 3.3995 -0.2468 H	9 H9 -2.3124 -2.1183 2.4610 H

10 C10	-2.9065	2.4596	-0.1746	C	10 C10	-1.0166	-1.2163	1.0173	C
11 H11	-3.4591	3.3971	-0.2412	H	11 C11	-0.4276	0.0009	0.5674	C
12 C12	-5.0553	1.2103	-0.0773	C	12 C12	-0.7405	-2.4330	0.2994	C
13 H13	-5.6044	2.1498	-0.1421	H	13 H13	-1.1968	-3.3488	0.6418	H
14 C14	-3.6504	1.2313	-0.0838	C	14 N14	4.7181	-0.0002	-6.1969	N
15 C15	-5.7465	-0.0014	0.0114	C	15 C15	4.6412	-1.0954	-5.4083	C
16 H16	-6.8361	-0.0016	0.0156	H	16 H16	5.2665	-1.9277	-5.6793	H
17 C17	-5.0540	-1.2128	0.0948	C	17 C17	3.8071	-1.1245	-4.3091	C
18 H18	-5.6022	-2.1525	0.1638	H	18 H18	3.8016	-2.0108	-3.6999	H
19 C19	-3.6492	-1.2331	0.0909	C	19 C19	3.0179	-0.0038	-3.9849	C
20 C20	-2.9039	-2.4610	0.1761	C	20 C20	2.1250	-0.0042	-2.8153	C
21 H21	-3.4554	-3.3989	0.2464	H	21 C21	1.5493	-1.2042	-2.3671	C
22 C22	-1.5388	-2.4625	0.1704	C	22 H22	1.7274	-2.1267	-2.8970	H
23 H23	-0.9861	-3.3998	0.2355	H	23 C23	0.6963	-1.2195	-1.2560	C
24 C24	-0.7988	-1.2316	0.0762	C	24 C24	0.4276	-0.0009	-0.5674	C
25 C25	-1.5148	-0.0003	-0.0037	C	25 C25	0.0809	-2.4346	-0.7900	C
26 C26	-2.9375	-0.0007	0.0010	C	26 H26	0.2836	-3.3517	-1.3207	H
27 C27	2.7765	0.0017	-0.0087	C	27 C27	3.9760	1.0926	-5.9172	C
28 C28	3.5199	-1.0850	-0.5112	C	28 H28	4.0769	1.9279	-6.5868	H
29 H29	3.0333	-1.9538	-0.9464	H	29 C29	3.1252	1.1171	-4.8288	C
30 C30	4.9002	-1.0574	-0.4983	C	30 H30	2.5355	2.0017	-4.6666	H
31 H31	5.5007	-1.8758	-0.8860	H	31 C31	1.8513	1.1972	-2.1417	C
32 C32	4.8961	1.0665	0.4840	C	32 H32	2.3124	2.1183	-2.4610	H
33 H33	5.4944	1.8848	0.8749	H	33 C33	1.0166	1.2163	-1.0173	C
34 C34	3.5158	1.0906	0.4948	C	34 C34	0.7405	2.4330	-0.2994	C
35 H35	3.0263	1.9600	0.9253	H	35 H35	1.1968	3.3488	-0.6418	H
36 C36	7.0429	-0.0108	0.0398	C	36 C36	-4.6412	1.0954	5.4083	C
37 H37	7.4170	1.0123	-0.0421	H	37 H37	-5.2665	1.9277	5.6793	H
38 H38	7.3685	-0.4523	0.9873	H	38 C38	-3.8071	1.1245	4.3091	C
39 H39	7.4235	-0.6027	-0.7958	H	39 H39	-3.8016	2.0108	3.6999	H
					40 C40	-1.5493	1.2042	2.3671	C
					41 H41	-1.7274	2.1267	2.8970	H
					42 C42	-0.6963	1.2195	1.2560	C
					43 C43	-0.0809	2.4346	0.7900	C
					44 H44	-0.2836	3.3517	1.3207	H
					45 C45	-5.6478	-0.0058	7.3360	C
					46 H46	-5.5818	0.9378	7.8582	H
					47 H47	-6.6562	-0.1565	6.9736	H
					48 H48	-5.3803	-0.8046	8.0107	H
					49 C49	5.6478	0.0058	-7.3360	C
					50 H50	6.6562	0.1565	-6.9736	H
					51 H51	5.5818	-0.9378	-7.8582	H
					52 H52	5.3803	0.8046	-8.0107	H
**1H**					**2H**				
1 N1	5.9389	-0.0000	-0.0000	N	1 N1	-7.7602	-0.0001	0.0000	N
2 C2	0.9978	1.2130	-0.0725	C	2 H2	-8.7682	-0.0002	0.0001	H
3 H3	1.5330	2.1577	-0.1631	H	3 C3	-2.8233	1.2066	-0.1466	C
4 C4	1.7033	0.0000	0.0000	C	4 H4	-3.3546	2.1338	-0.2910	H
5 C5	0.9979	-1.2129	0.0725	C	5 C5	-3.5271	0.0001	-0.0000	C
6 H6	1.5330	-2.1577	0.1631	H	6 C6	-2.8233	-1.2065	0.1465	C
7 C7	-0.4026	-1.2315	0.0850	C	7 H7	-3.3547	-2.1336	0.2909	H
8 C8	-1.1432	-2.4618	0.1828	C	8 C8	-1.4230	-1.2244	0.1564	C
9 H9	-0.5908	-3.3990	0.2532	H	9 C9	-0.7104	0.0000	-0.0000	C
10 C10	-2.5082	-2.4597	0.1858	C	10 C10	-1.4230	1.2245	-0.1564	C
11 H11	-3.0604	-3.3969	0.2593	H	11 C11	-4.9984	0.0001	-0.0000	C
12 C12	-4.6574	-1.2112	0.0919	C	12 C12	0.6822	2.4476	-0.3225	C

13 H13	-5.2061	-2.1504	0.1635 H	
14 C14	-3.2526	-1.2318	0.0928 C	
15 C15	-5.3493	-0.0000	0.0000 C	
16 H16	-6.4389	-0.0000	0.0000 H	
17 C17	-4.6575	1.2112	-0.0919 C	
18 H18	-5.2061	2.1503	-0.1635 H	
19 C19	-3.2526	1.2318	-0.0928 C	
20 C20	-2.5082	2.4596	-0.1858 C	
21 H21	-3.0604	3.3969	-0.2593 H	
22 C22	-1.1432	2.4618	-0.1828 C	
23 H23	-0.5909	3.3990	-0.2532 H	
24 C24	-0.4026	1.2315	-0.0850 C	
25 C25	-1.1177	0.0000	0.0000 C	
26 C26	-2.5404	-0.0000	0.0000 C	
27 C27	3.1722	0.0000	-0.0000 C	
28 C28	3.9067	1.0817	0.5314 C	
29 H29	3.4099	1.9359	0.9829 H	
30 C30	5.2869	1.0596	0.5222 C	
31 H31	5.8998	1.8595	0.9280 H	
32 C32	5.2869	-1.0596	-0.5222 C	
33 H33	5.8998	-1.8595	-0.9280 H	
34 C34	3.9067	-1.0817	-0.5314 C	
35 H35	3.4098	-1.9358	-0.9828 H	
36 H36	6.9569	-0.0000	0.0000 H	

13 H13	1.2286	3.3697	-0.4454 H
14 C14	-0.6821	2.4476	-0.3225 C
15 H15	-1.2285	3.3697	-0.4454 H
16 C16	-5.7294	1.1188	0.4500 C
17 H17	-5.2355	1.9943	0.8312 H
18 C18	-7.1096	1.0965	0.4405 C
19 H19	-7.7169	1.9163	0.7785 H
20 C20	-7.1096	-1.0966	-0.4404 C
21 H21	-7.7167	-1.9165	-0.7785 H
22 C22	-5.7293	-1.1187	-0.4500 C
23 H23	-5.2354	-1.9942	-0.8313 H
24 N24	7.7602	0.0001	-0.0000 N
25 H25	8.7682	0.0001	-0.0001 H
26 C26	2.8233	-1.2066	0.1466 C
27 H27	3.3546	-2.1338	0.2910 H
28 C28	3.5271	-0.0001	0.0000 C
29 C29	2.8233	1.2065	-0.1465 C
30 H30	3.3547	2.1336	-0.2909 H
31 C31	1.4230	1.2245	-0.1564 C
32 C32	0.7104	-0.0000	0.0000 C
33 C33	1.4230	-1.2245	0.1564 C
34 C34	4.9984	-0.0001	0.0000 C
35 C35	-0.6822	-2.4476	0.3225 C
36 H36	-1.2286	-3.3697	0.4454 H
37 C37	0.6821	-2.4476	0.3225 C
38 H38	1.2285	-3.3697	0.4454 H
39 C39	5.7294	-1.1188	-0.4500 C
40 H40	5.2355	-1.9943	-0.8312 H
41 C41	7.1096	-1.0965	-0.4405 C
42 H42	7.7169	-1.9163	-0.7785 H
43 C43	7.1096	1.0966	0.4405 C
44 H44	7.7168	1.9164	0.7785 H
45 C45	5.7293	1.1187	0.4500 C
46 H46	5.2354	1.9942	0.8313 H

Table S5. Absorption wavelengths (λ_{cal}, nm), oscillator strengths (*f*), and main electronic transitions calculated at the PBE0-GD3BJ and CAM-B3LYP-GD3BJ level in THF for compounds **1**, **2**, **1M**, **2M**, **1H**, and **2H**. Corresponding excited states (S_n) are given in brackets. Experimental wavelengths (λ_{exp}, nm) and their extinction coefficients (ε, $M^{-1}cm^{-1}$) measured in MeCN are given for comparison.

Compound	λ_{exp} (MeCN)	ε / M^{-1} cm^{-1}		λ_{cal}	*f* (S_n)	Main electronic transitions (weight %)[a]
**1**	362	1100	PBE0	354	0.013 (S_1)	H→L+1 (76%); H-1 →L (22%)
	337	37000		339	0.36 (S_2)	H →L (90%)
	322	21000		277	1.60 (S_5)	H-1 →L+1 (91%)
	308		CAM-B3LYP	330	0.007 (S_1)	H→L+1 (58%); H-1 →L (33%)
	276			321	0.43 (S_2)	H →L (90%)
				259	1.81 (S_5)	H-1 →L+1 (86%)
**1M**	418	2900	PBE0	488	0.016 (S_1)	H →L (98%)
	337	45000		363	0.14 (S_2)	H-1→L (72%); H→L+1 (26%)
	317	68500		338	1.01 (S_3)	H→L+1 (58%); H-1→L (24%);
	269					H→L+2 (16%)
				260	0.21 (S_{11})	H→L+3 (29%); H-3→L (+21%);
						H-1→L+2 (16%); H-1→L+1 (15%); H-2→L+1 (14%)
			CAM-B3LYP	390	0.03 (S_1)	H →L (+87%)
				324	0.005 (S_2)	H→L+1 (58%); H-1 →L (35%)
				306	1.94 (S_3)	H-1→L (54%); H→L+1 (35%)
				248	0.22 (S_7)	H→L+3 (30%); H-1→L+1 (23%);
						H-2→L+1 (17%)
**1H**	421		PBE0	498	0.015 (S_1)	H →L (90%)
	337			365	0.14 (S_2)	H-1 →L (75%); H →L+1 (23%)
	319			338	0.83 (S_3)	H →L+1 (58%); H →L+2 (20%);
						H-1 →L (20%)
				261	0.23 (S_{11})	H→L+3 (30%); H-1 →L+2 (18%);
						H-2 →L+1 (18%); H-1 →L+1 (16%); H-3 →L(12%)
			CAM-B3LYP	396	0.03 (S_1)	H →L (89%)
				329	0.002 (S_2)	H→L+1 (54%); H-1 →L (39%)
				306	1.81 (S_3)	H-1→L (51%); H→L+1 (39%)
				248	0.30 (S_8)	H→L+3 (39%); H-1→L+1 (28%);
						H-2→L+1 (11%)
**2**	363	1400	PBE0	370	0.016 (S_1)	H→L+1 (76%); H-1 →L (21%)
	339	35000		342	0.22 (S_2)	H →L (85%); H-1 →L+1 (14%)
	324			296	2.57 (S_4)	H-1 →L+1 (84%); H →L (13%)
	312		CAM-B3LYP	342	0.01 (S_1)	H →L+1 (63%); H-1 →L (30%)
	291			322	0.32 (S_2)	H →L (86%); H-1 →L+1 (11%)
				276	2.08 (S_3)	H-1 →L+1 (81%); H →L (12%)
**2M**	437	4300	PBE0	480	0.027 (S_1)	H →L (97%)
	352			410	0.00 (S_2)	H→L+1 (99%)
	337	80000		366	0.57 (S_3)	H-1→L (82%); H→L+2 (17%)
	320	120000		335	1.96 (S_4)	H→L+2 (78%); H-1→L (16%)
				236	0.20 (S_{20})	H-5→L (62%); H-1→L+4 (12%);
						H-5→L+1 (12%)
			CAM-B3LYP	397	0.04 (S_1)	H →L (97%)
				332	0.17 (S_2)	H-1→L (56%); H→L+2 (37%)
				310	2.89 (S_4)	H→L+2 (54%); H-1→L (+34%)
				215	0.26 (S_{18})	H-1→L+4 (58%); H-5→L (18%);
						H-5→L+1 (12%)
**2H**	428	1530	PBE0	490	0.028 (S_1)	H →L (98%)
	341	30500		416	0.00 (S2)	H→L+1 (99%)
	321	50000		368	0.51 (S_3)	H-1 → L (81%); H→ L+2 (17%)
				335	1.85 (S_4)	H → L+2 (78%); H-1→ L (16%)
				235	0.23 (S_{20})	H-5→L (69%); H-6→L+1 (11%); H-1→L+4 (11%)
			CAM-B3LYP	403	0.04 (S_1)	H →L (89%)
				333	0.18 (S_2)	H-1→L (59%); H→L+2 (34%)
				310	2.67 (S_4)	H→L+2 (60%); H-1→L (31%)
				215	0.30 (S_{19})	H-1→L+4 (61%); H-5→L (18%);
						H-6→L+1 (11%)

[a] H = HOMO and L = LUMO.

Table S6. Experimentally observed absorption and emission wavelengths (λ_{exp}, nm) and calculated absorption (PBE0-GD3BJ and CAM-B3LYP-GD3BJ) and emission (PBE0-GD3BJ/LANL2DZP) wavelengths for compounds **1**, **2**, **1M**, and **2M** in THF and MeCN.

		Absorption				Emission			
Compound	Method	λ_{calc}		λ_{exp}		λ_{calc}		λ_{exp}	
		THF	MeCN	THF	MeCN	THF	MeCN	THF	MeCN
**1**	PBE0	339	338		337	397	400		427
		277	276		322				405
	CAM-B3LYP	321	320		308				
		259	260		276				
**1M**	PBE0	363	353	339	337	563	535	586	599
		336	329	318	317				
		260	262	271	269				
	CAM-B3LYP	390	325						
		306	299						
		248	245						
**2**	PBE0	342	342		339	410	415		469
		296	296		324				442
					312				418
	CAM-B3LYP	322	322		291				
		276	275						
**2M**	PBE0	366	362		352	536	528		538
		340	334		337				
		240	240		320				
	CAM-B3LYP	332	329						
		310	306						
		220	220						

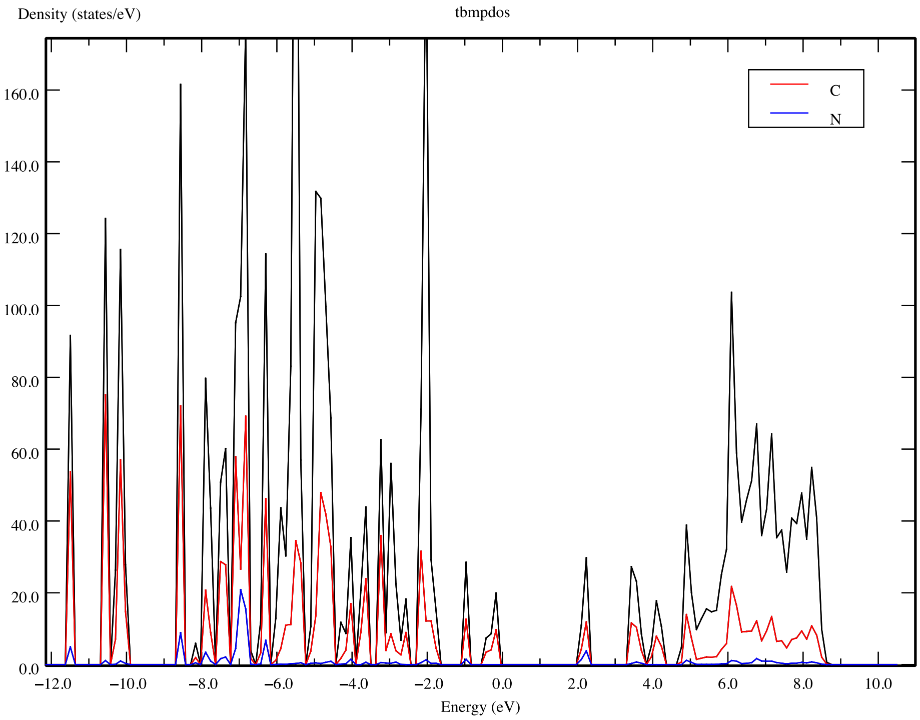

Figure S34: Density of states (DOS) of compound **2M**. Fermi level at 0 eV.

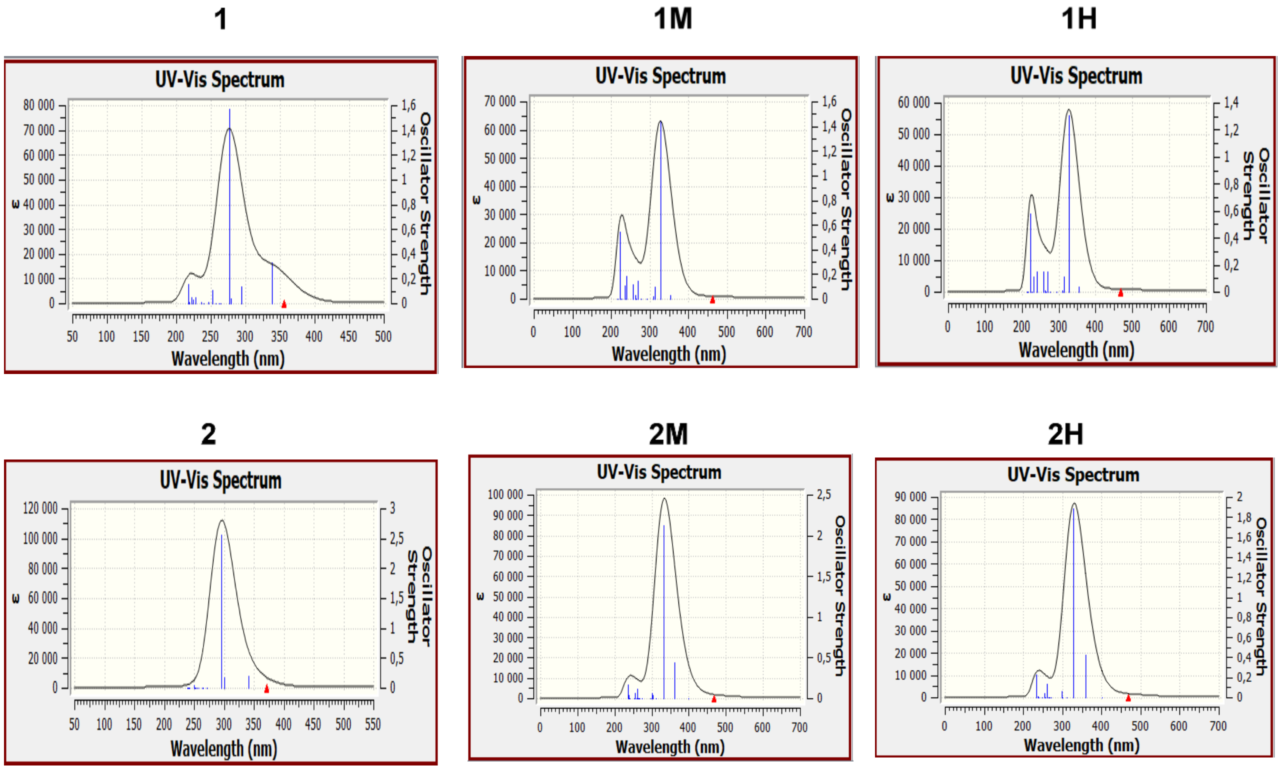

Figure S35: Simulated UV-Vis absorption spectra (PBE0-GD3BJ/LANL2DZP) in MeCN for **1**, **1H**, **1M**, **2**, **2H**, and **2M**.

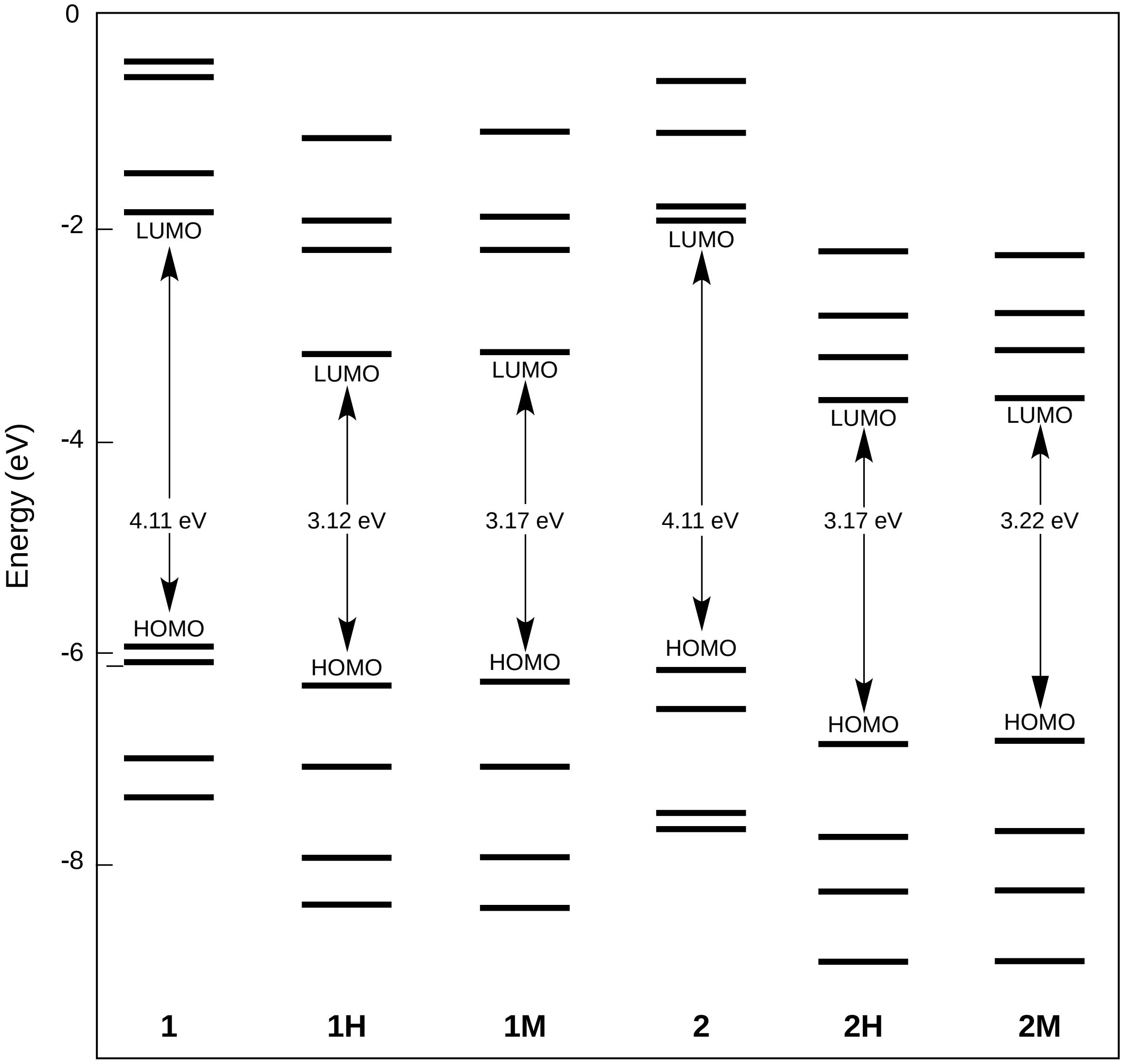

Figure S36: Molecular orbital energy diagrams of **1**, **1H**, **1M**, **2**, **2H**, and **2M**, calculated at the PBE0-GD3BJ/LANL2DZP level in THF.

Table S7. Calculated HOMO and LUMO energies (eV), and HOMO-LUMO gaps (ΔE, eV) at the PBE0-GD3BJ/LANL2DZP level in THF.

Compound	E_{HOMO}	E_{LUMO}	ΔE
**1**	-5.94	-1.83	4.11
**1-H**	-6.35	-3.23	3.12
**1-M**	-6.34	-3.17	3.17
**2**	-6.06	-1.94	4.11
**2-H**	-6.85	-3.68	3.17
**2-M**	-6.83	-3.61	3.22

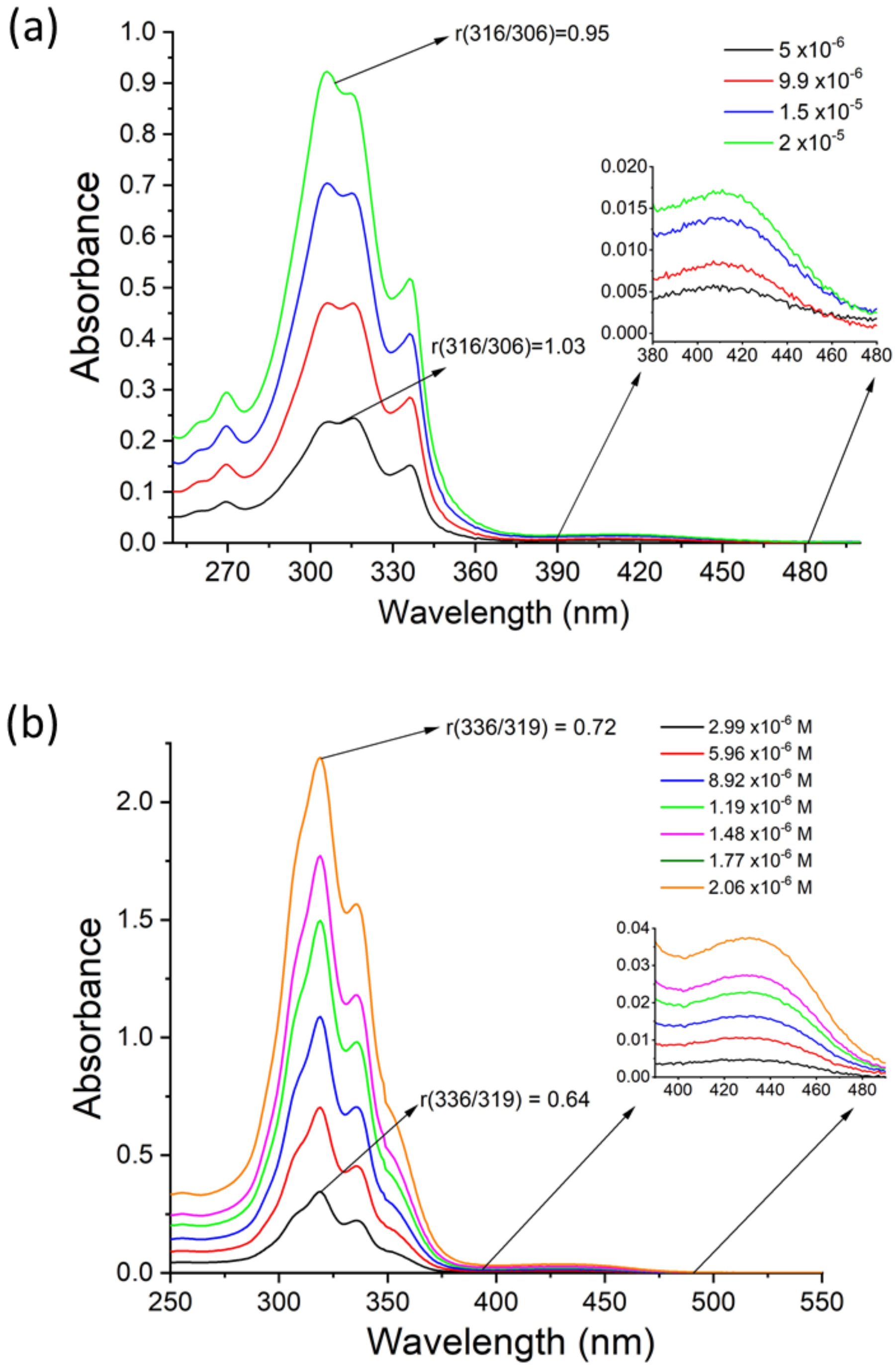

Figure S37: Concentration dependence of UV-Vis absorption of **1M** (a) and **2M** (b) at pH 7.0 with sodium cacodylate buffer, I = 50 mM.

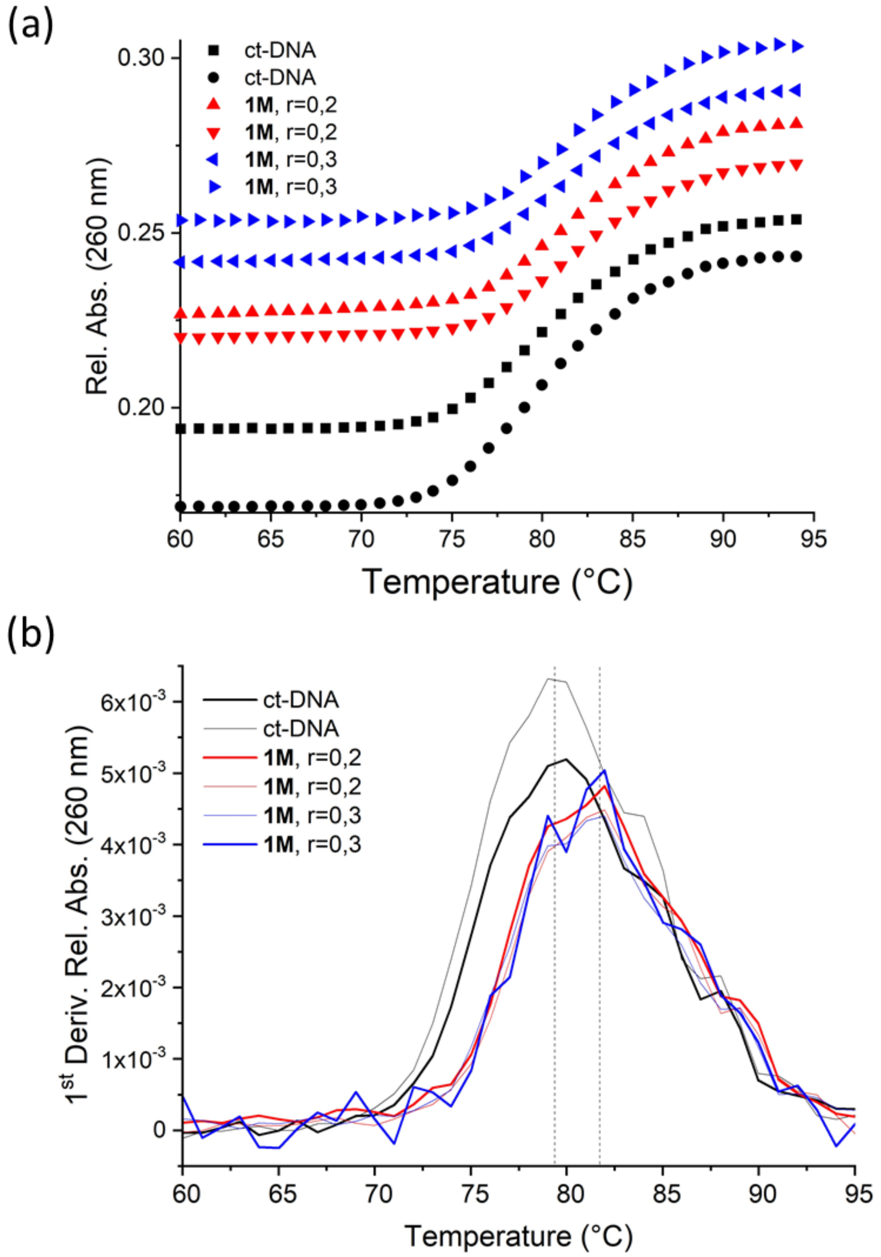

Figure S38: (a) Thermal denaturation curve of ct-DNA upon addition $r_{[\mathbf{1M}]/[ctDNA]}$ = 0.2 and r = 0.3 of **1M** at pH 7.0 (sodium cacodylate buffer, *I* = 0.05 M), and (b) first derivative of absorbance *vs.* temperature.

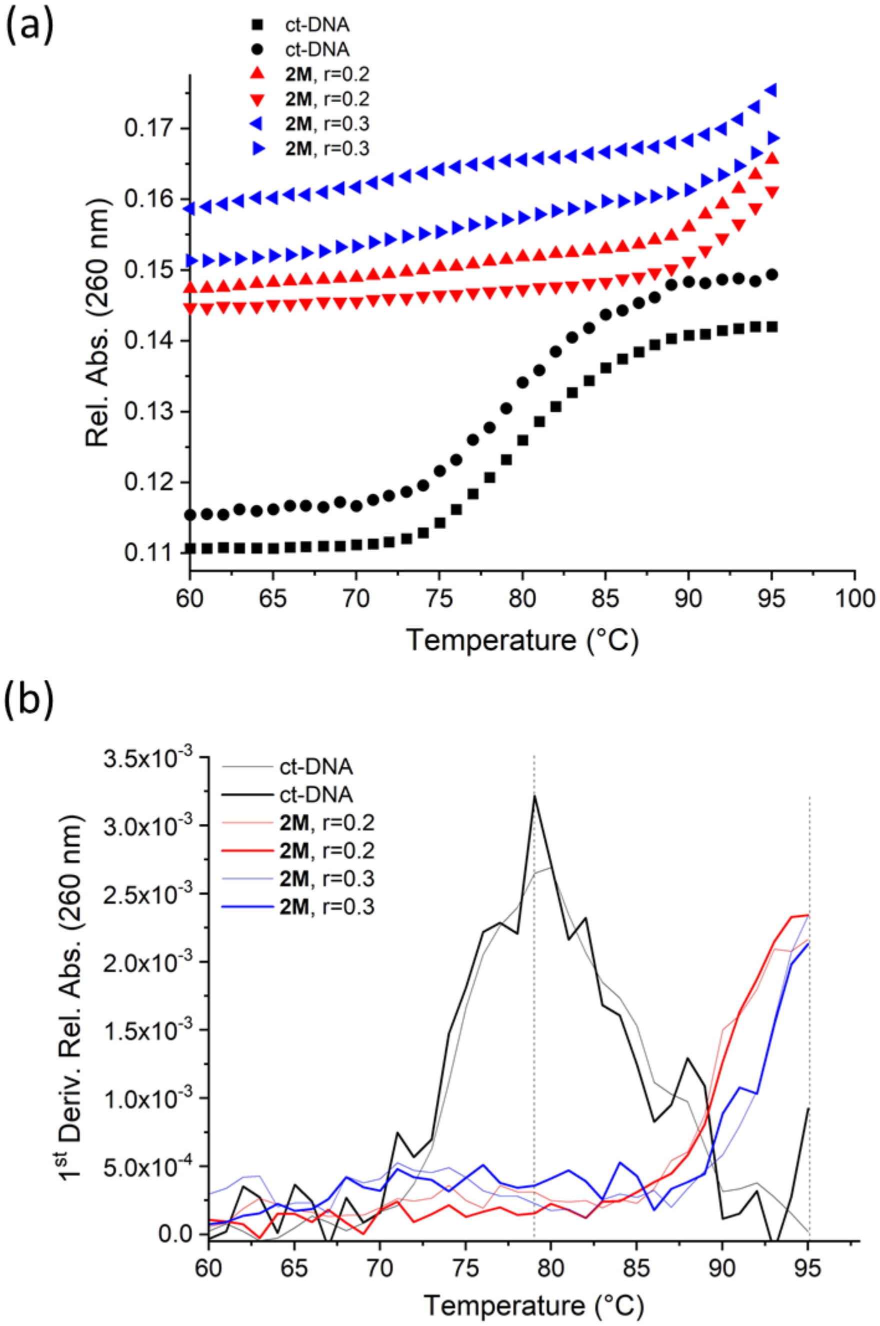

Figure S39: (a) Thermal denaturation curve of ct-DNA upon addition $r_{[\mathbf{2M}]/[ctDNA]}$ = 0.2 and r = 0.3 of **2M** at pH 7.0 (buffer sodium cacodylate, *I* = 0.05 M), and (b) first derivative of absorbance *vs*. temperature.

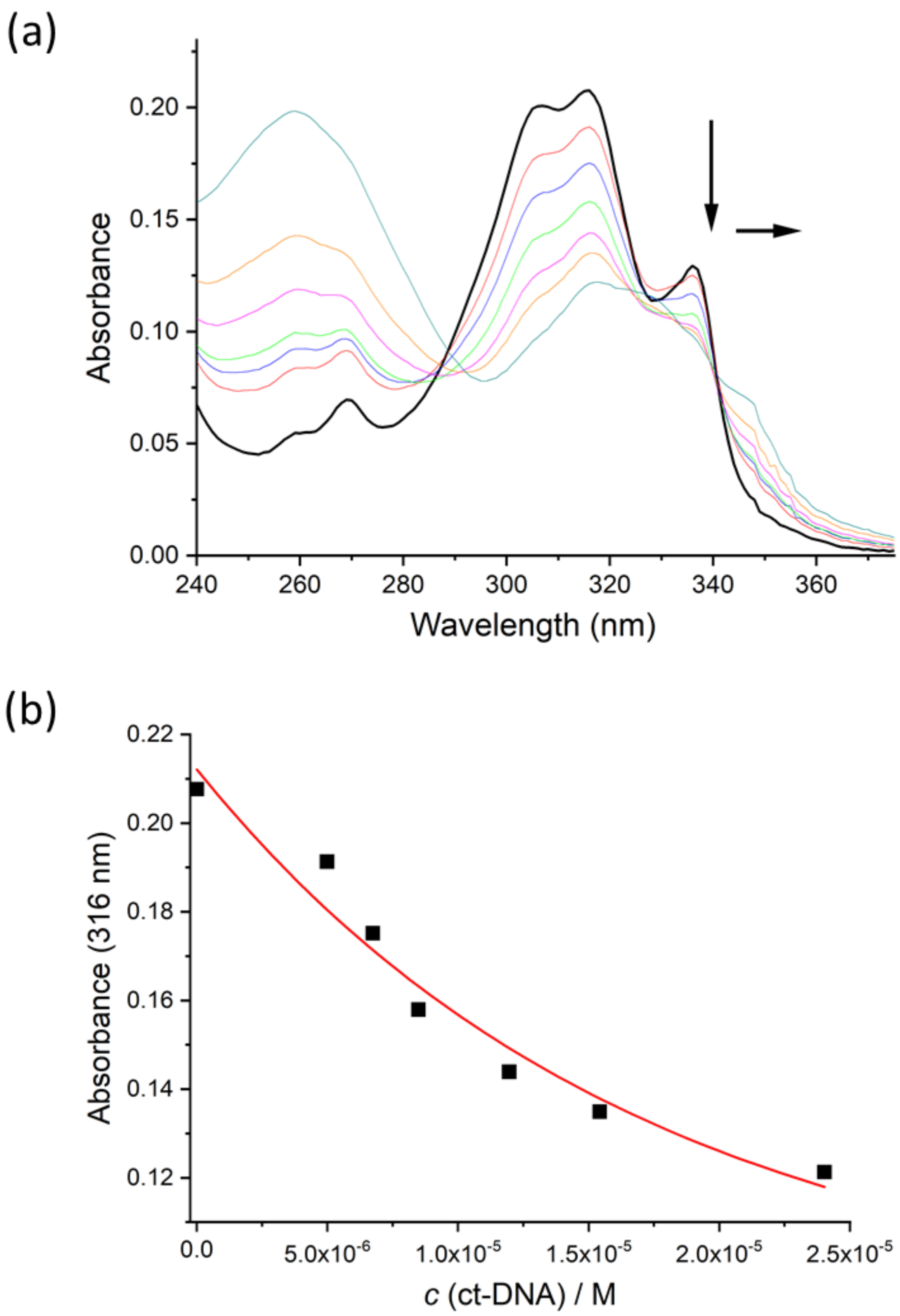

Figure S40: (a) Changes in UV-Vis absorption of **1M** ($c = 5 \times 10^{-6}$ M) upon titration with ct-DNA, and (b) dependence of absorbance of **1M** at λ_{max} = 316 nm on c(ct-DNA) at pH 7.0 with sodium cacodylate buffer, I = 0.05 M.

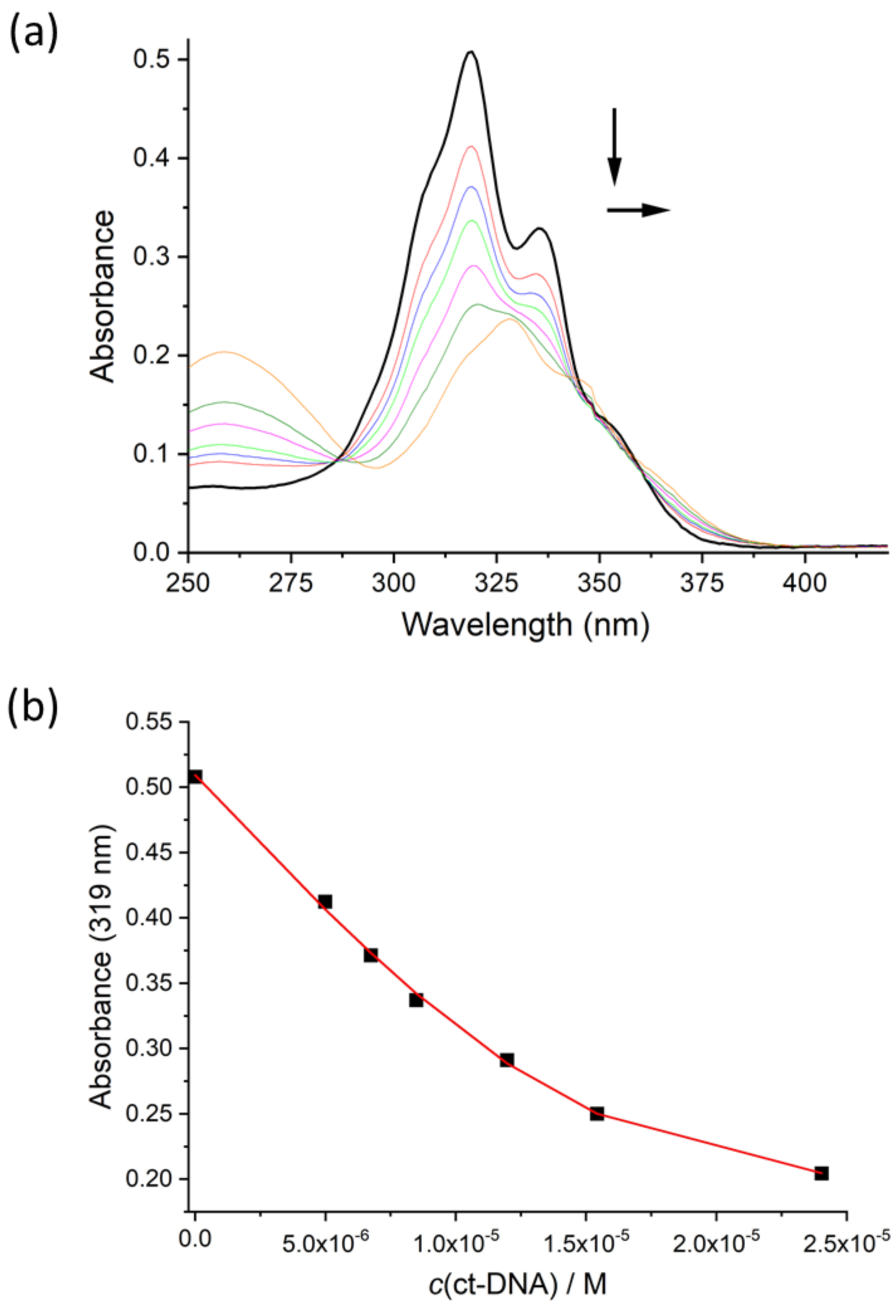

Figure S41: (a) Changes in UV-Vis absorption of **2M** (c = 5 × 10^{-6} M) upon titration with ct-DNA, and (b) dependence of absorbance of **2M** at λ_{max} = 319 nm on c(ct-DNA) at pH 7.0 with sodium cacodylate buffer, I = 0.05 M.

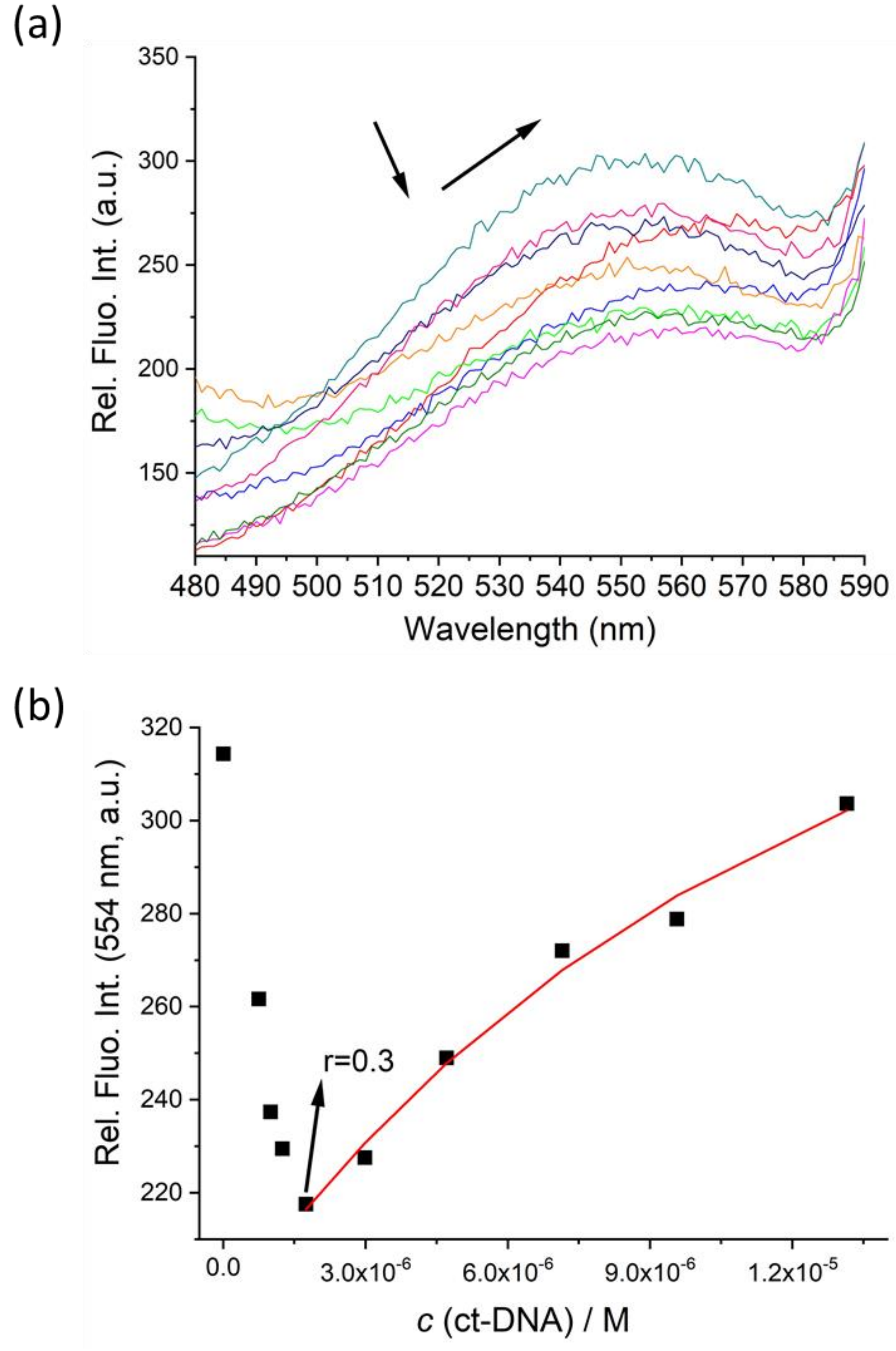

Figure S42: (a) Changes in fluorescence of **1M** (c = 5× 10^{-7} M) upon titration with ct-DNA, and (b) dependence of emission intensity of **1M** at λ_{max} = 554 nm on c(ct-DNA) at pH 7.0 with sodium cacodylate buffer, I = 0.05 M. The Scatchard fit (—) was applied only for a part of r << 0.3.

(a)

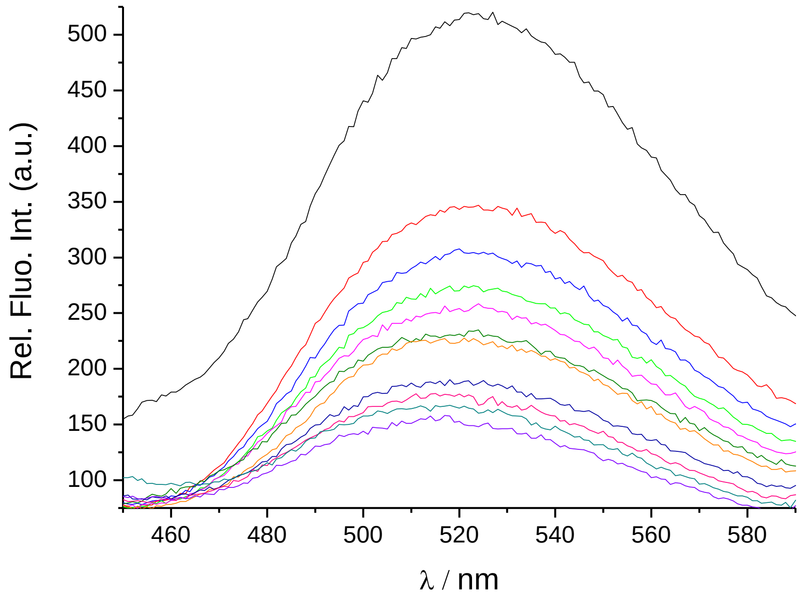

(b)

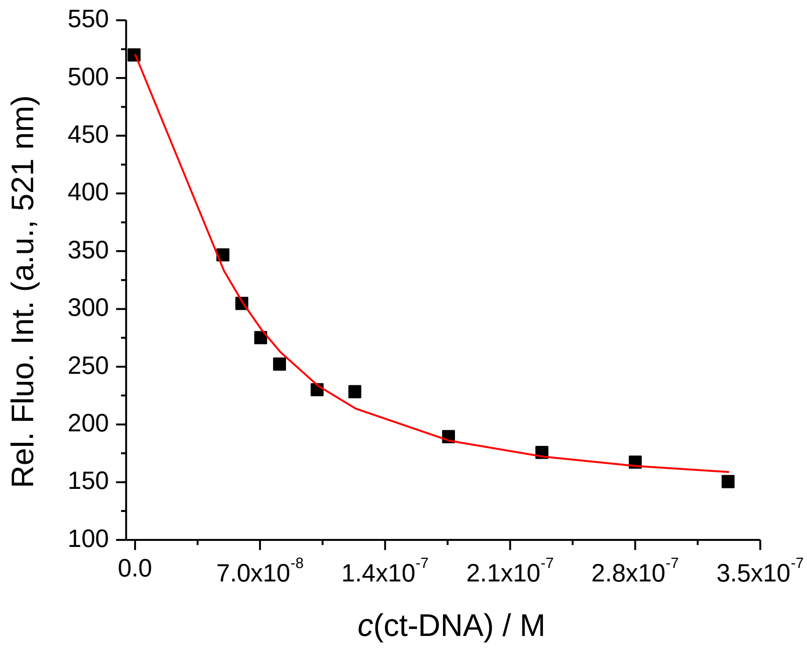

Figure S43. (a) Changes in the fluorescence spectrum of **2M** ($c = 3 \times 10^{-8}$ M) upon titration with ctDNA; (b) Dependence of **2M** intensity at λ_{max} = 521 nm on c(ctDNA), at pH 7.0, sodium cacodylate buffer, I = 0.05 M.

Reference

1. Q. Lu, G. K. Kole, A. Friedrich, K. Müller-Buschbaum, Z. Liu, X. Yu, T. B. Marder, *J. Org. Chem.* **2020**, *85*, 4256-4266.
